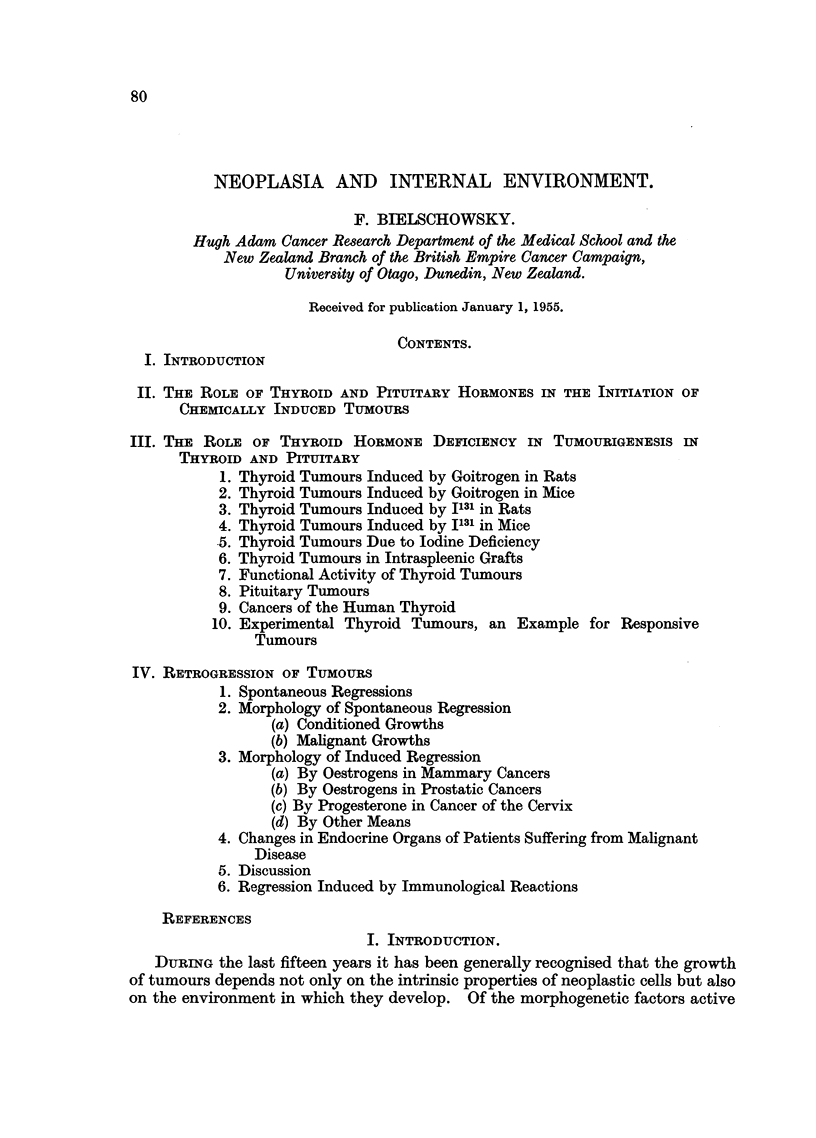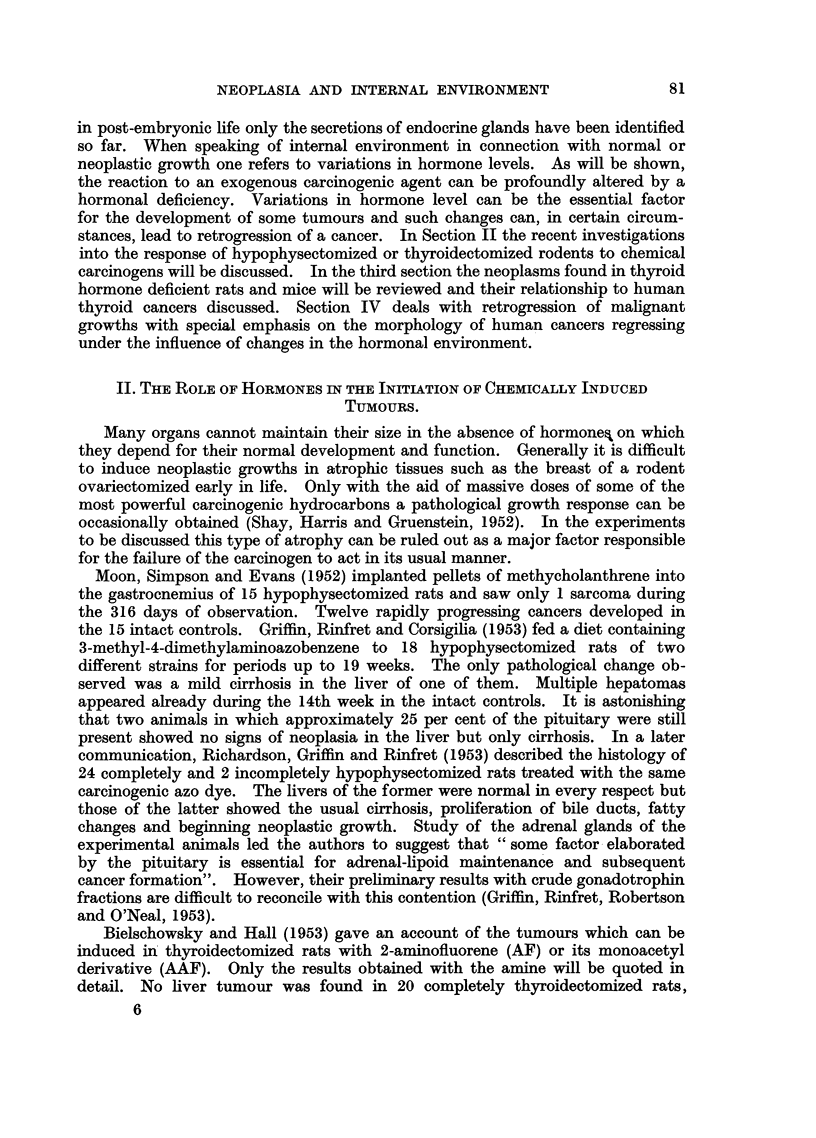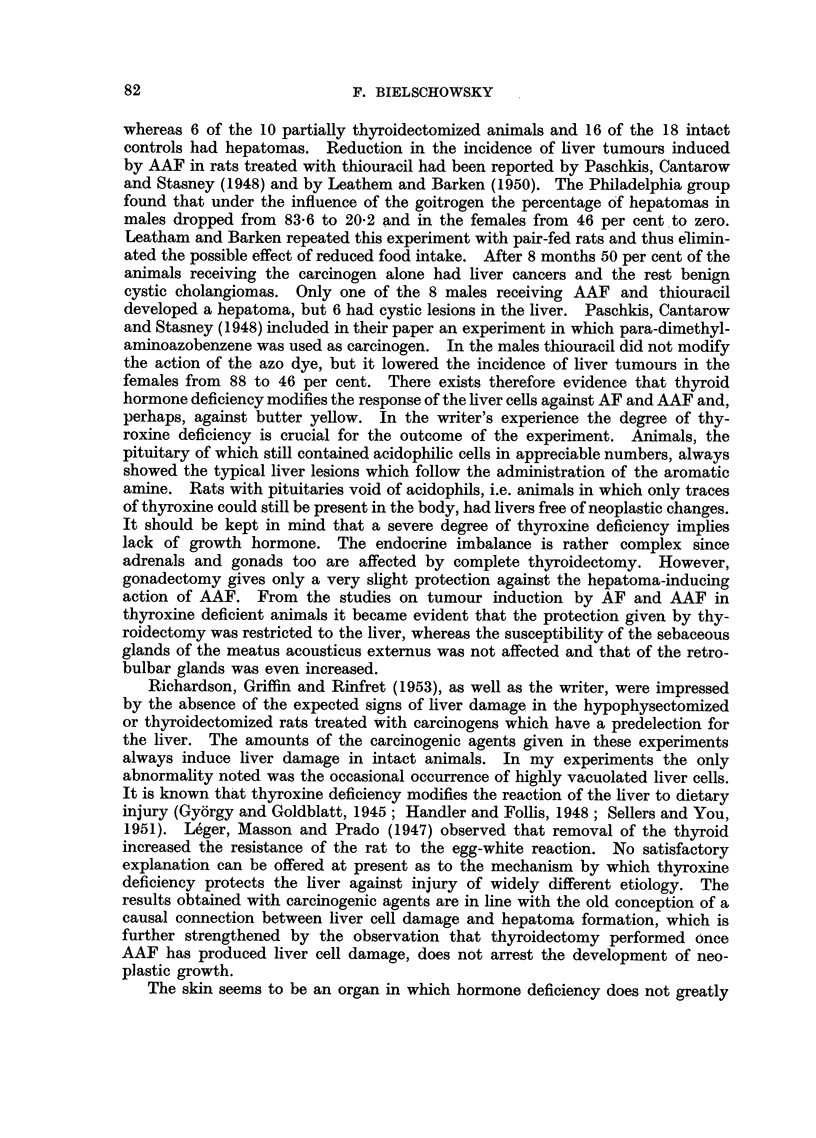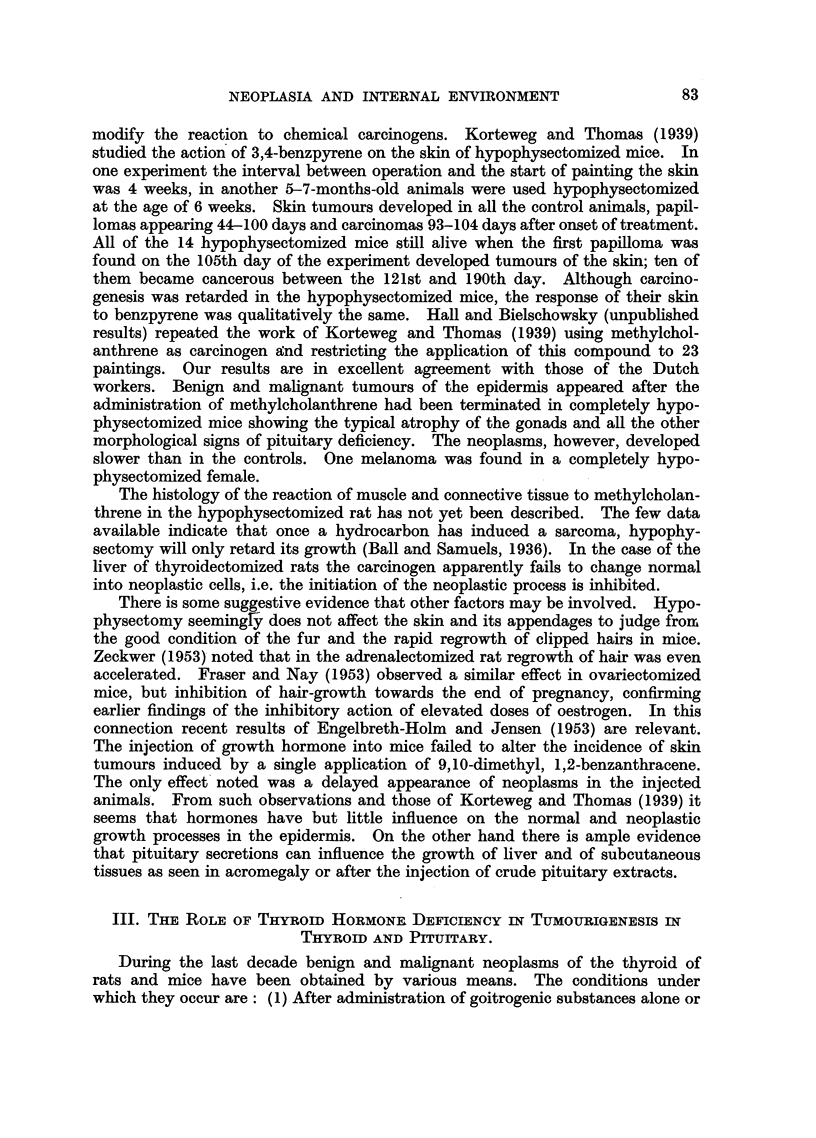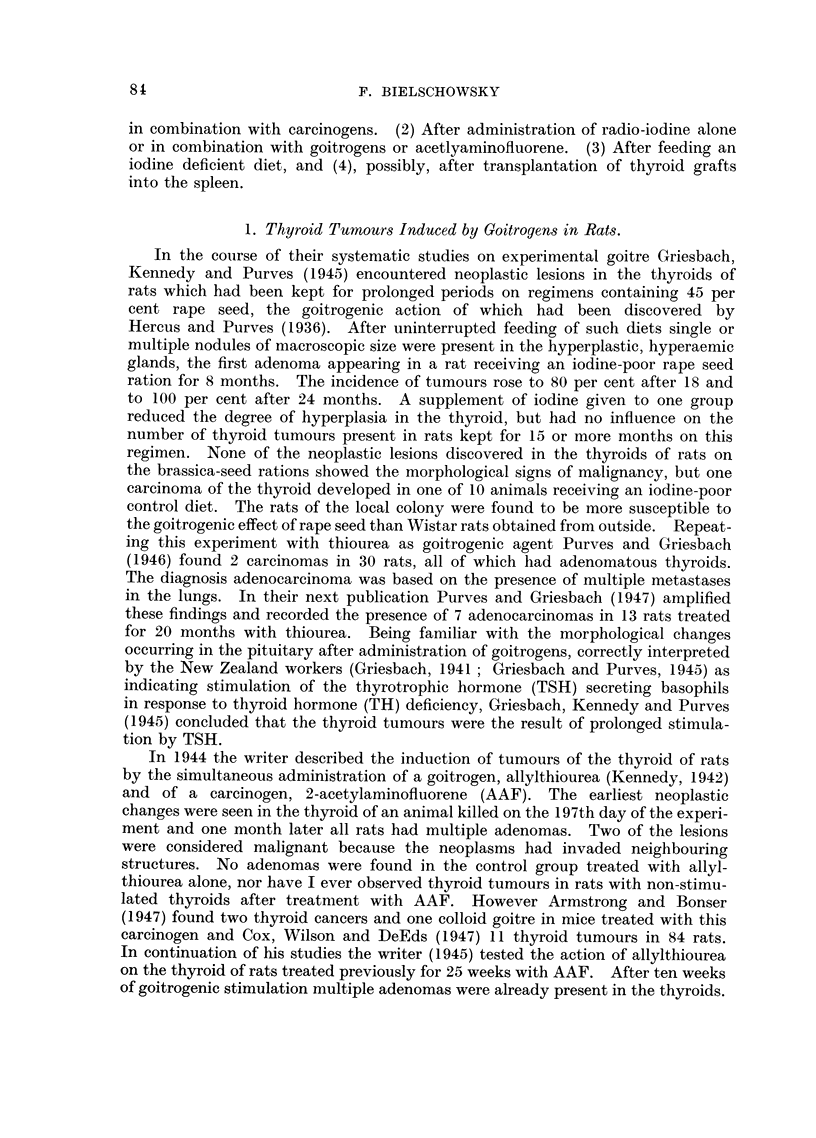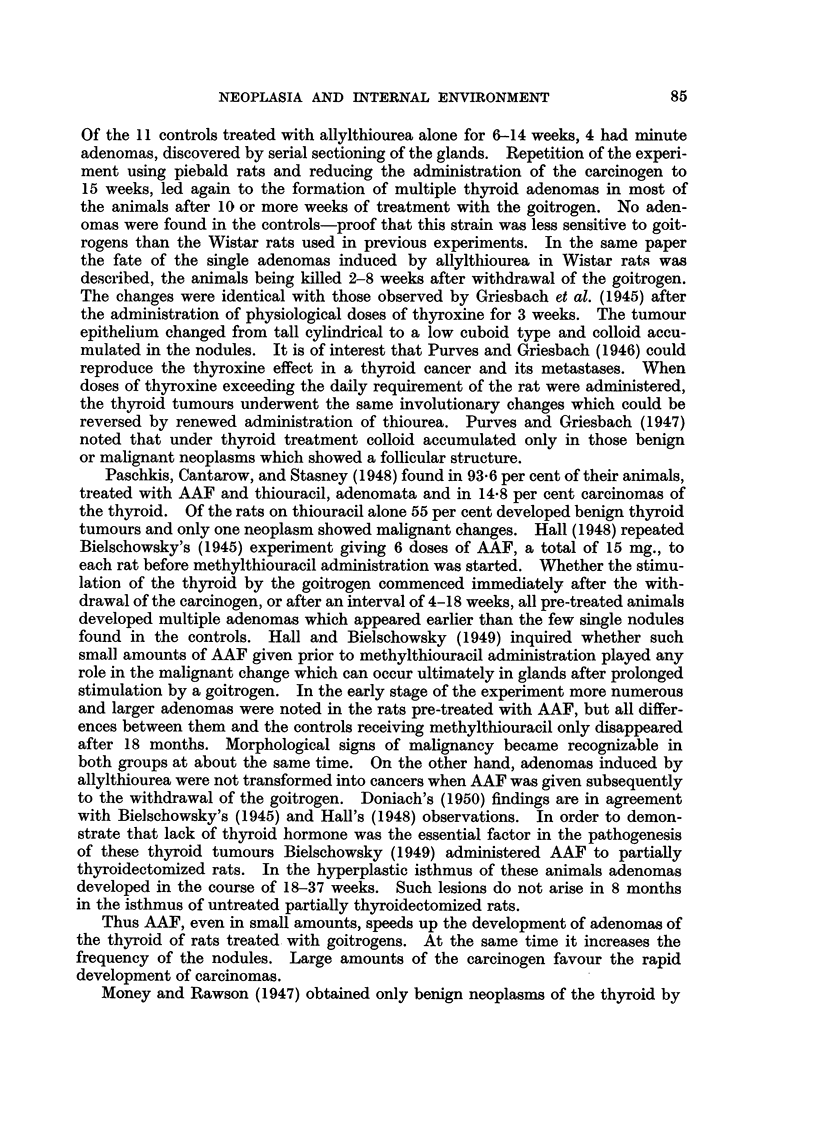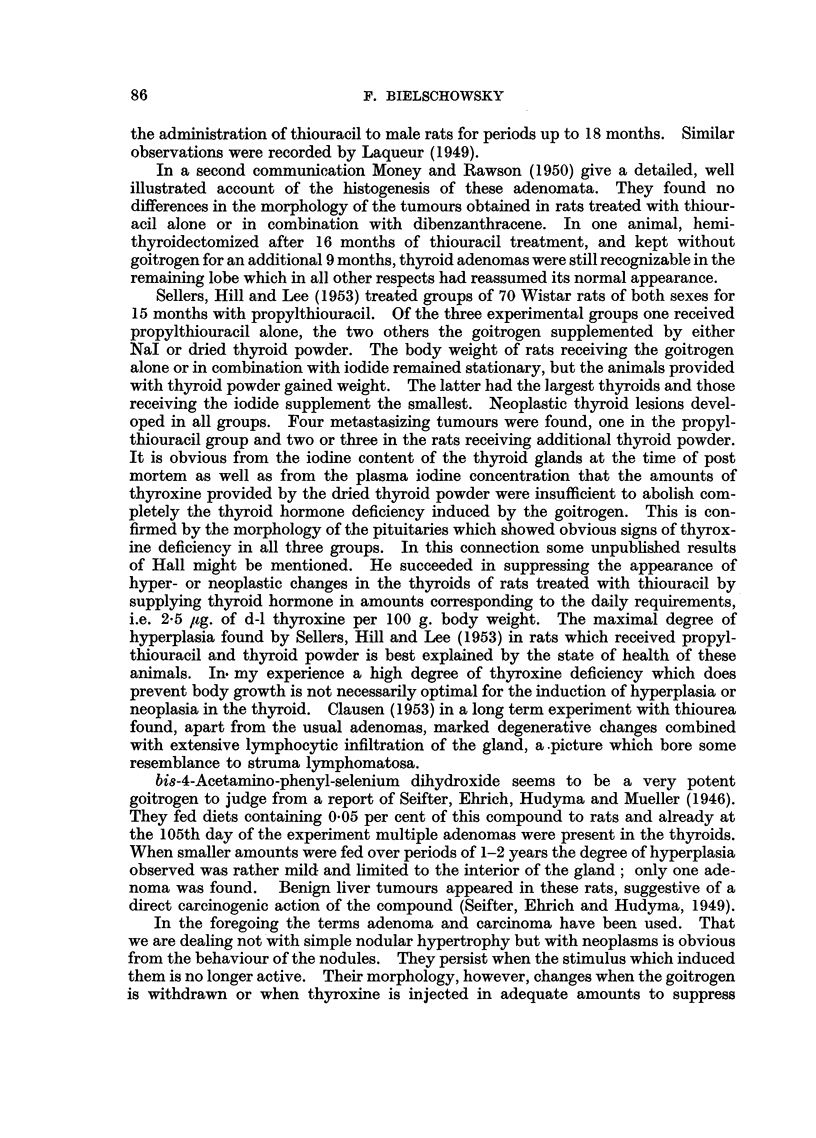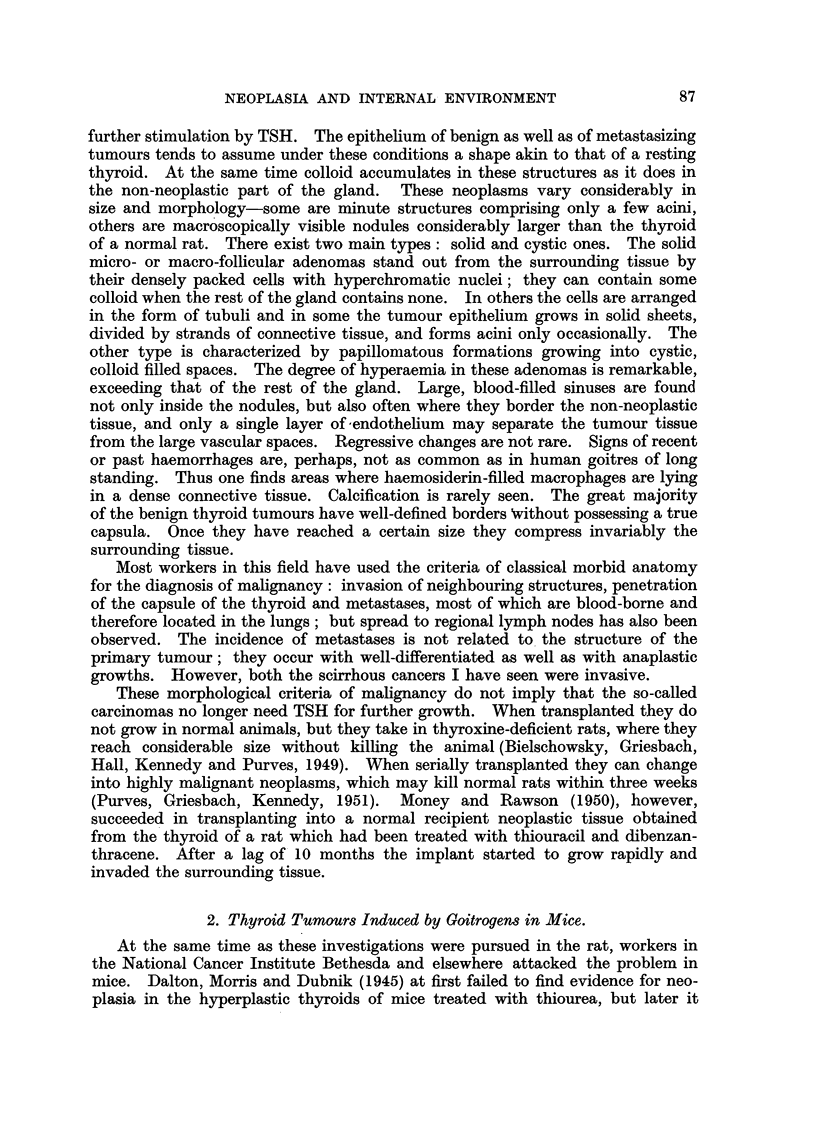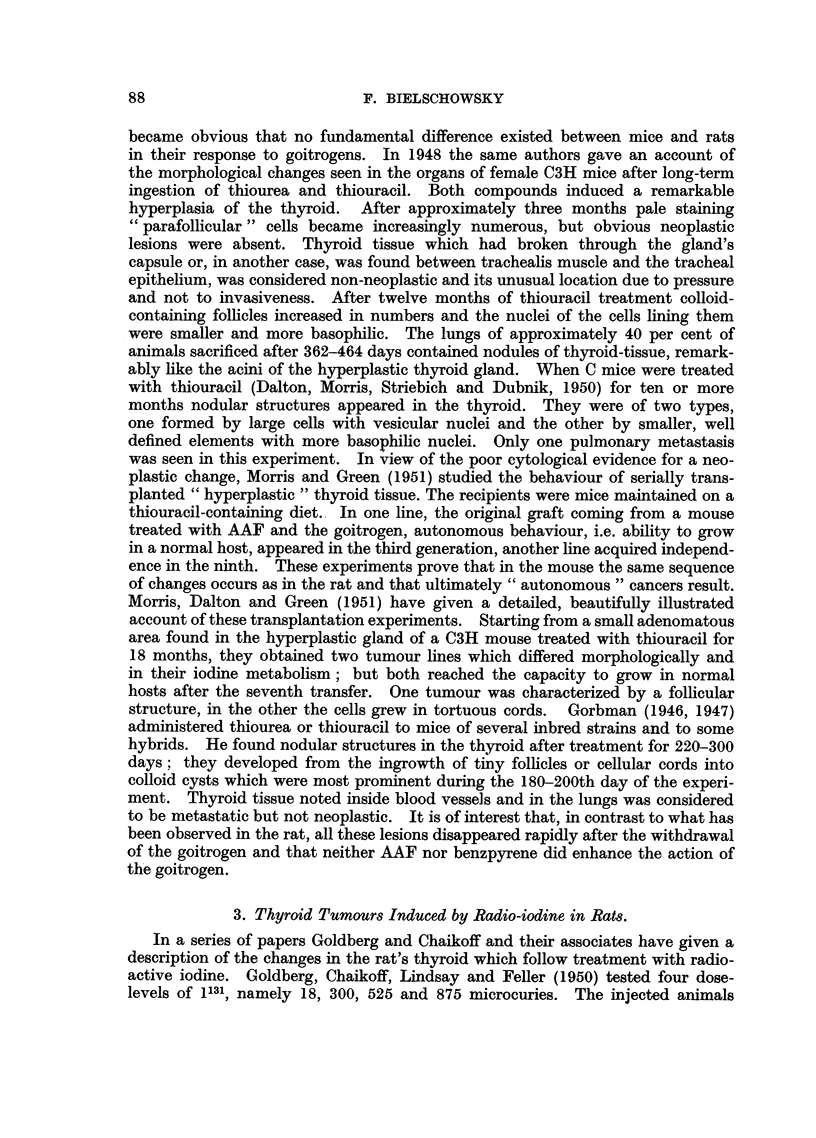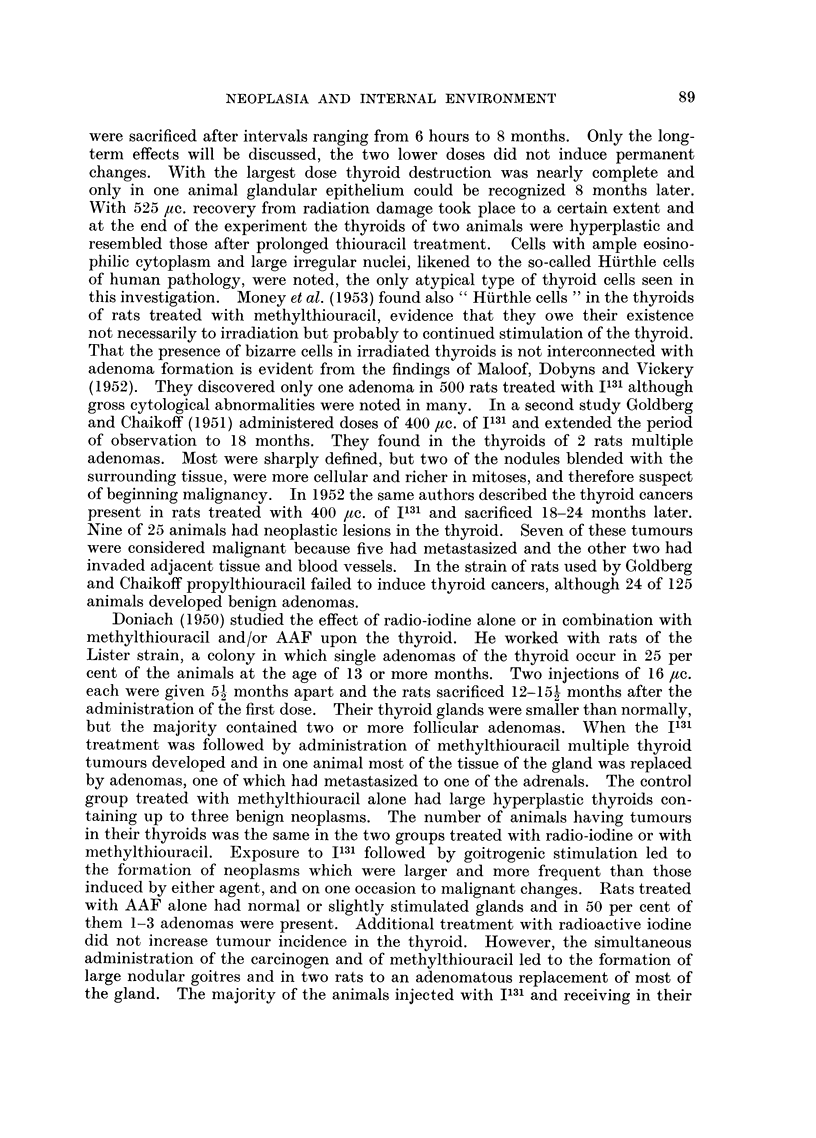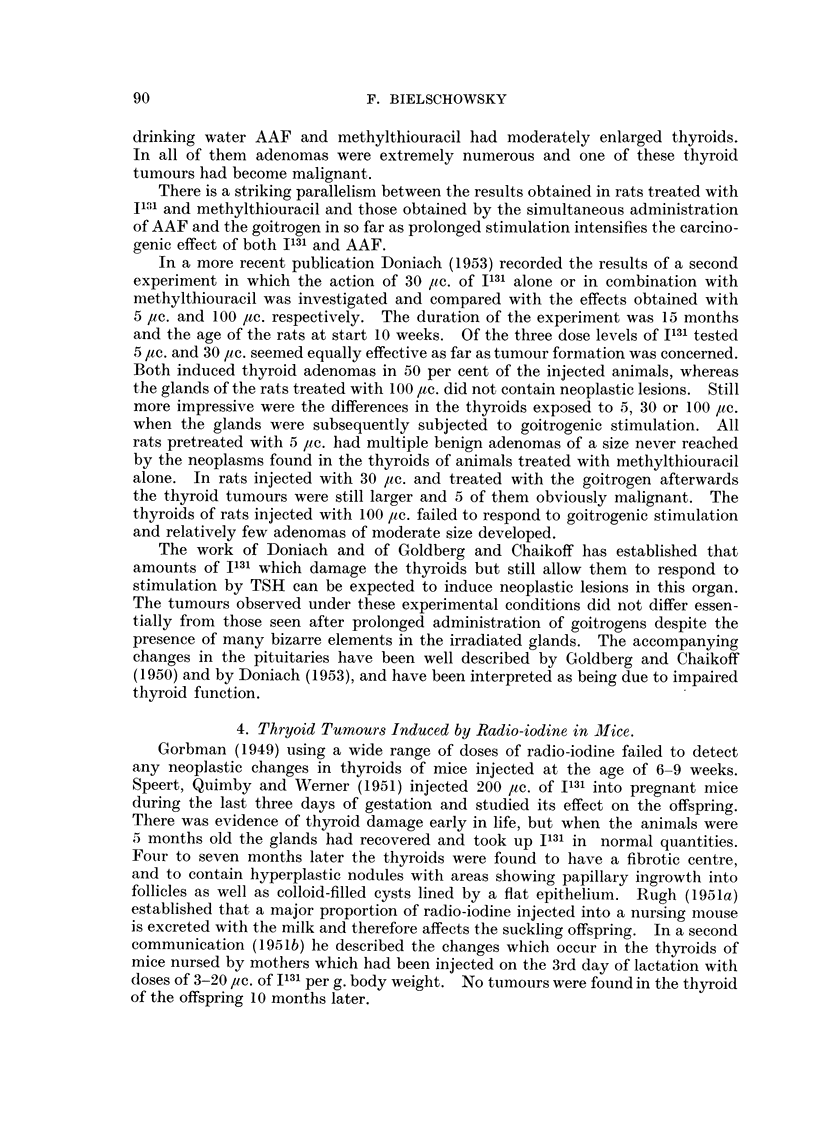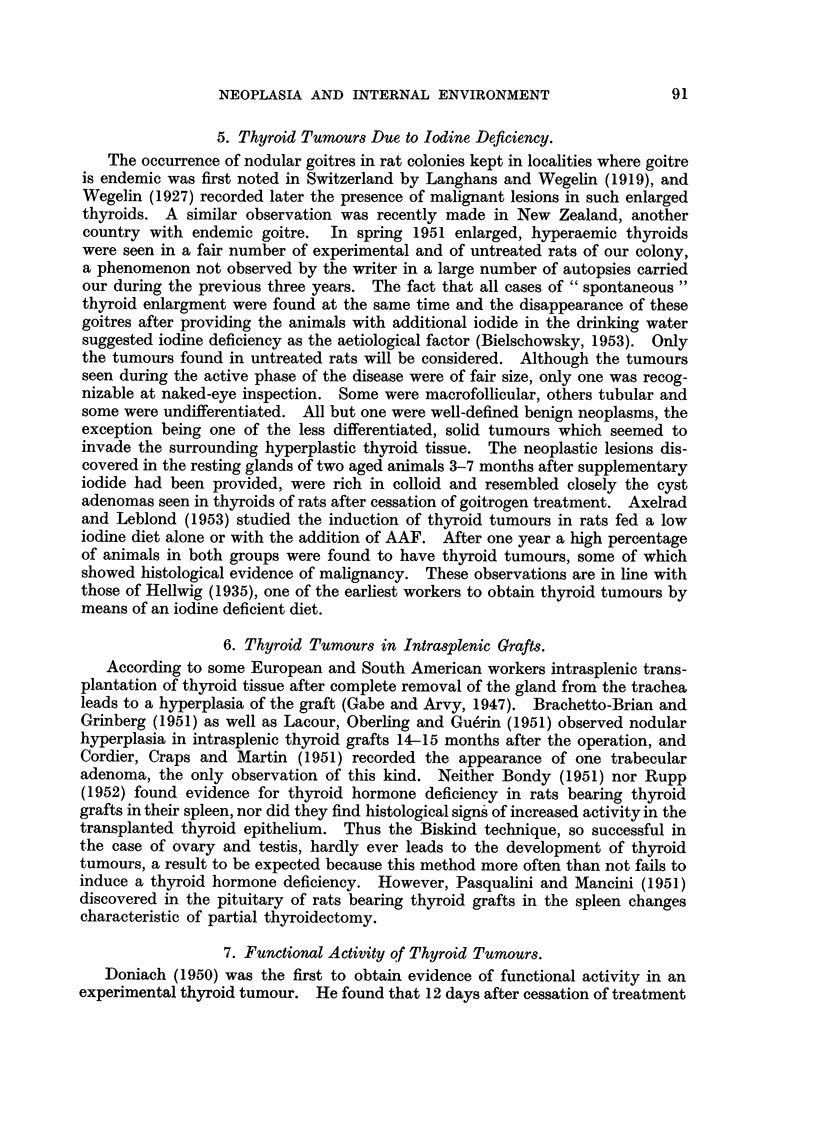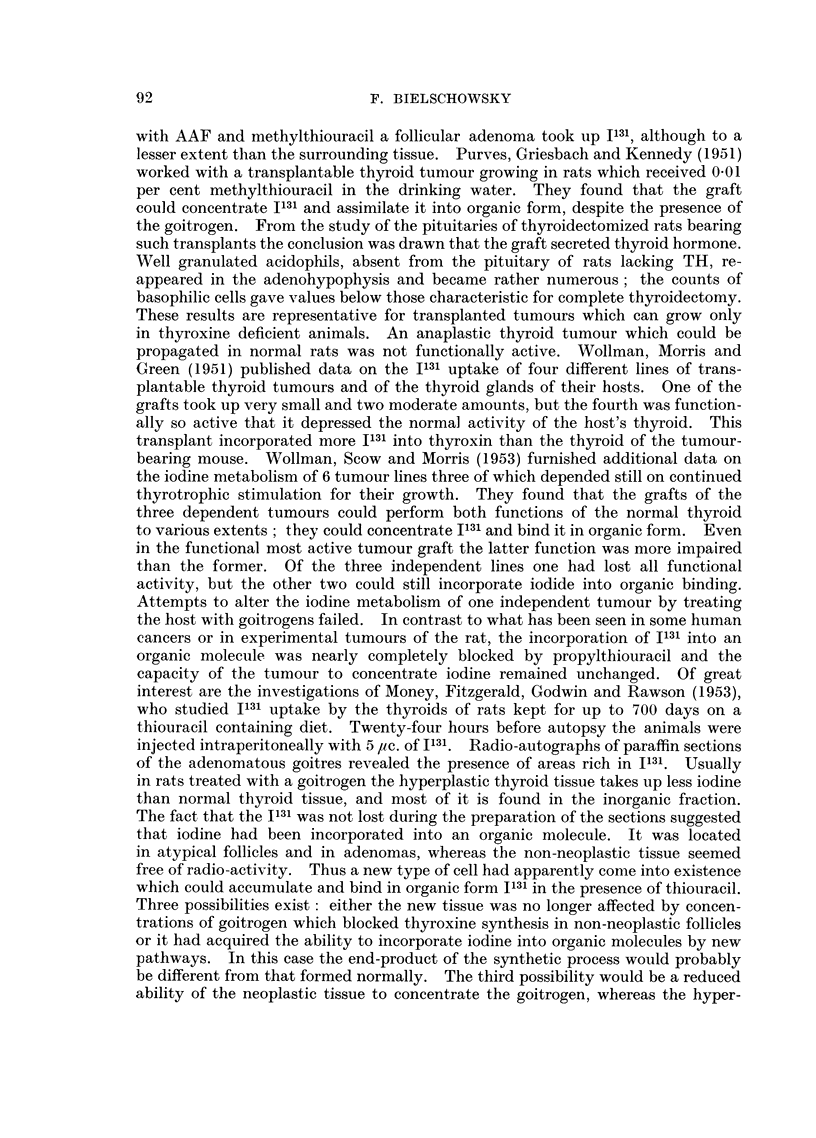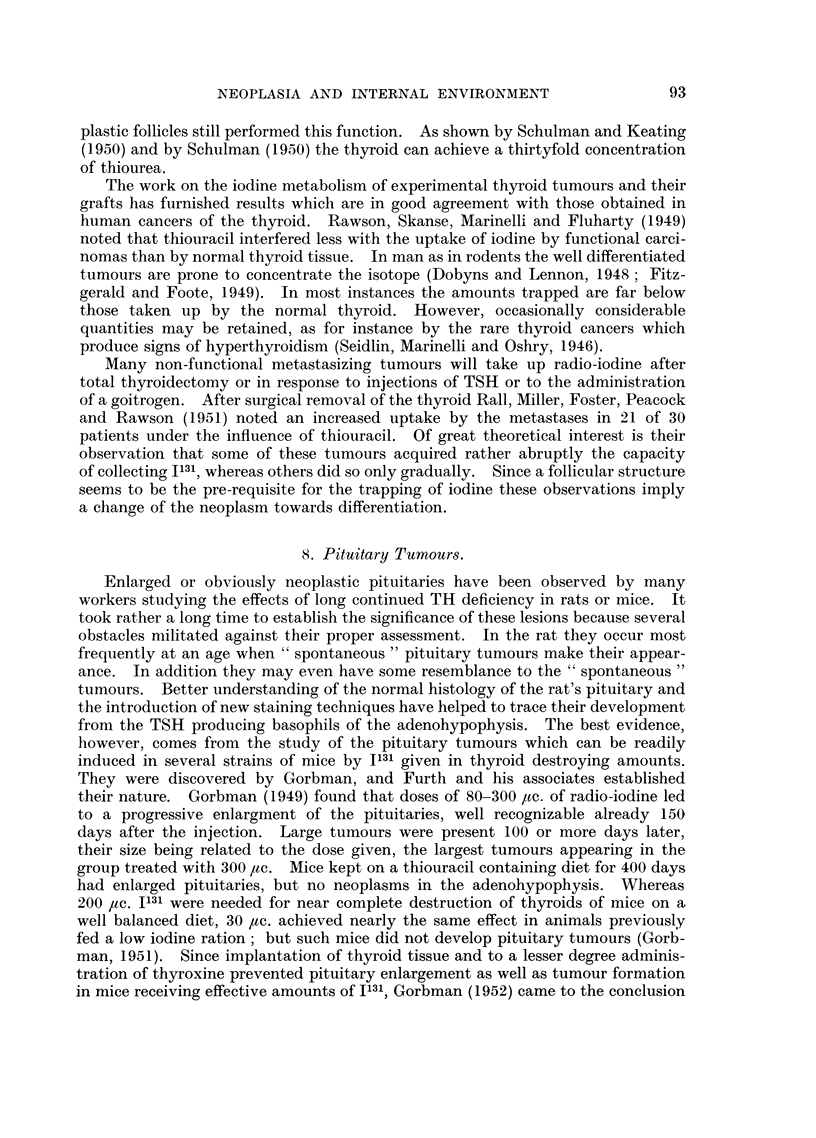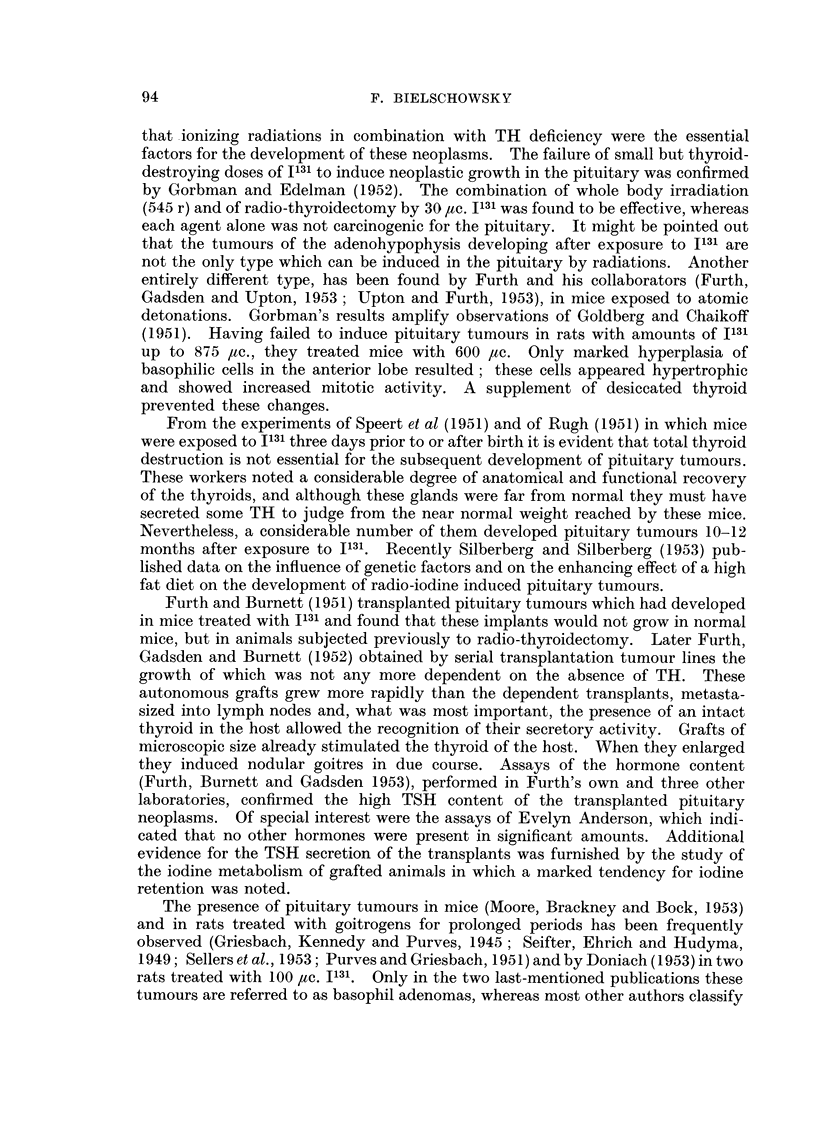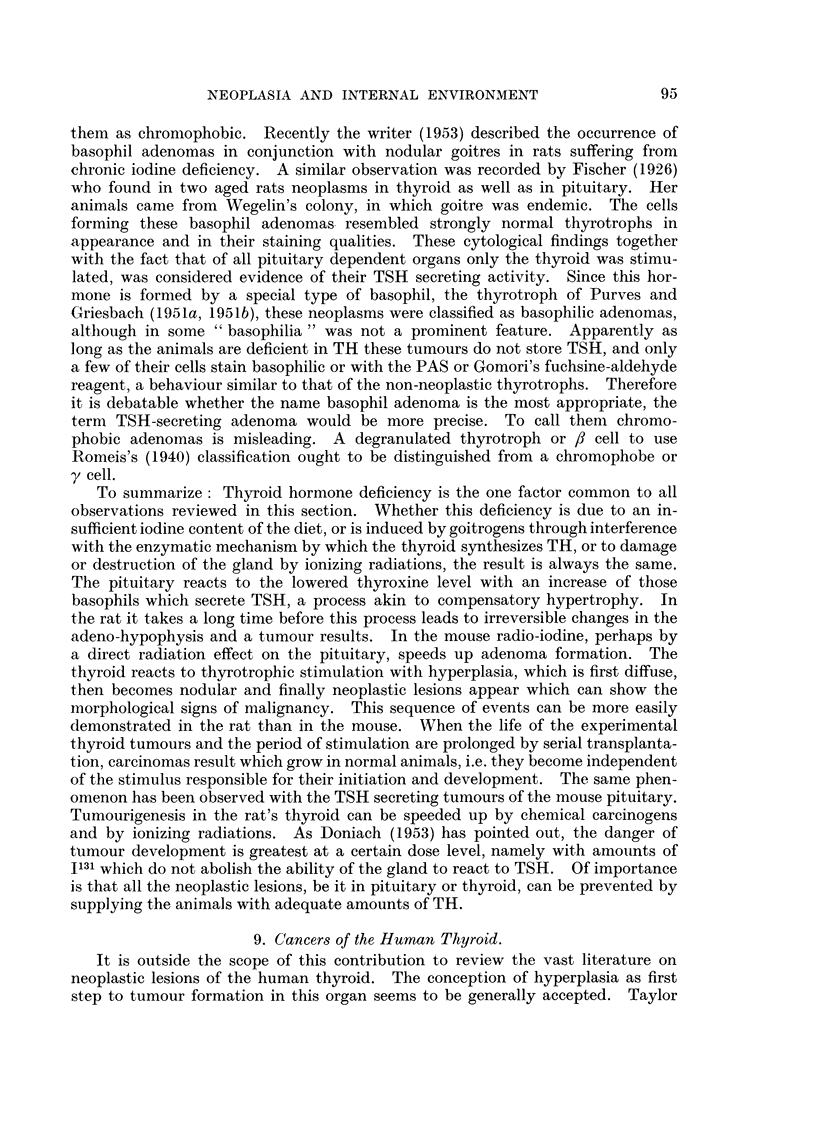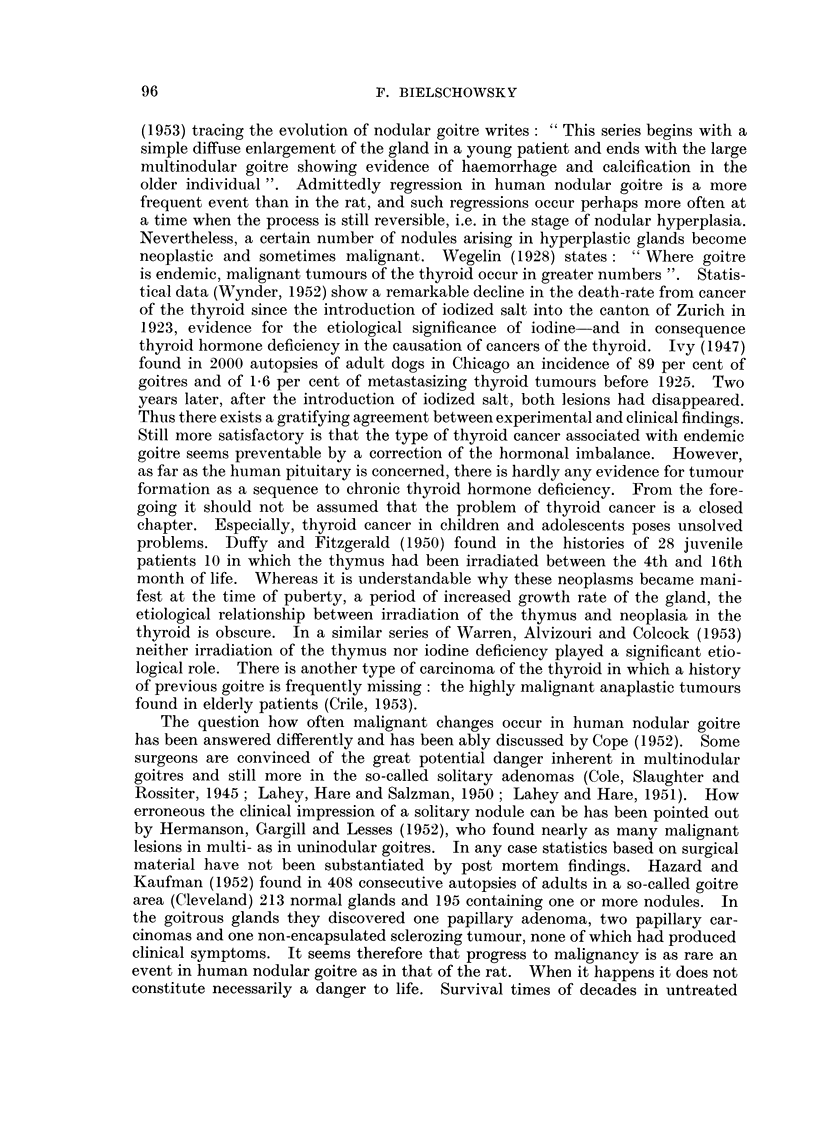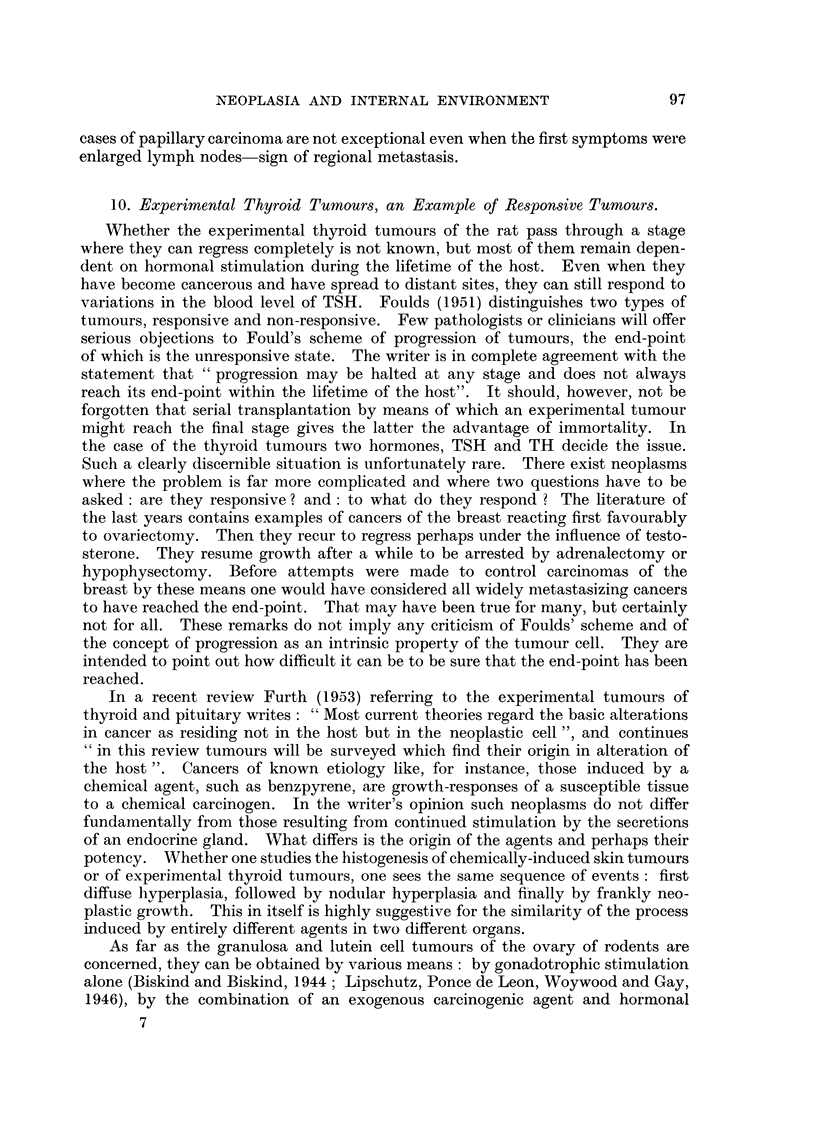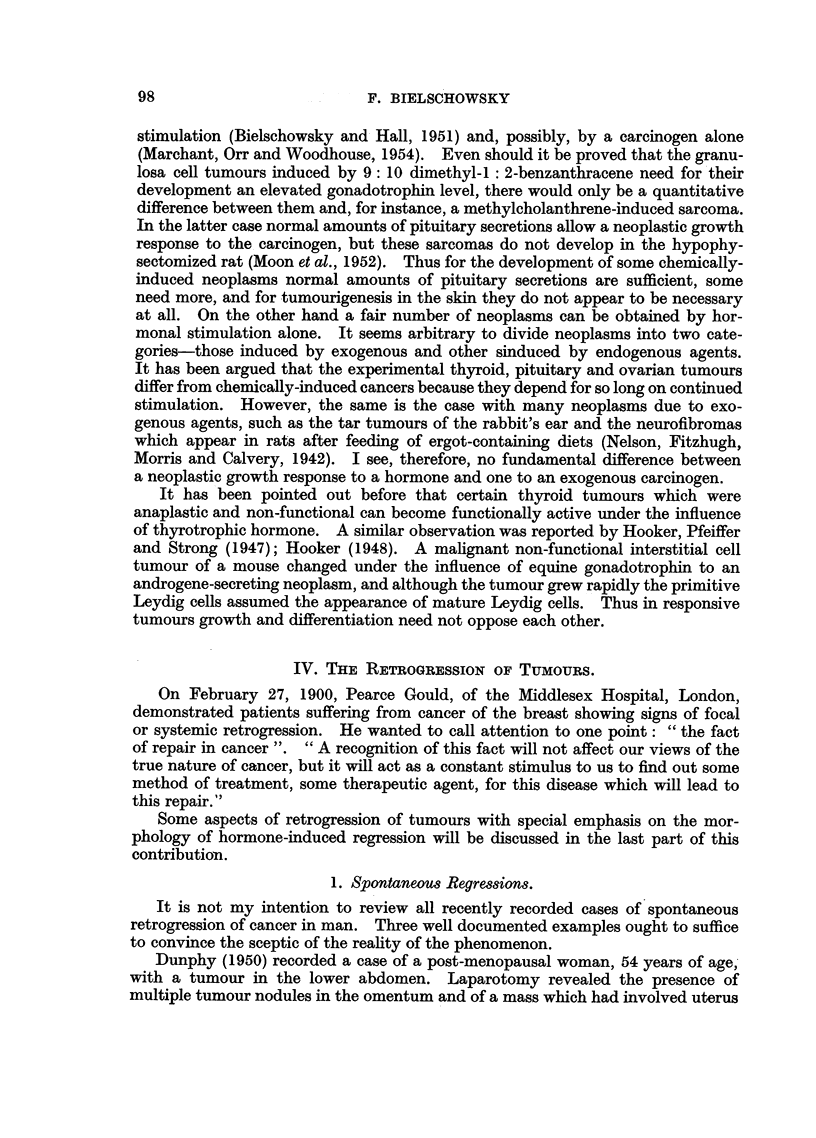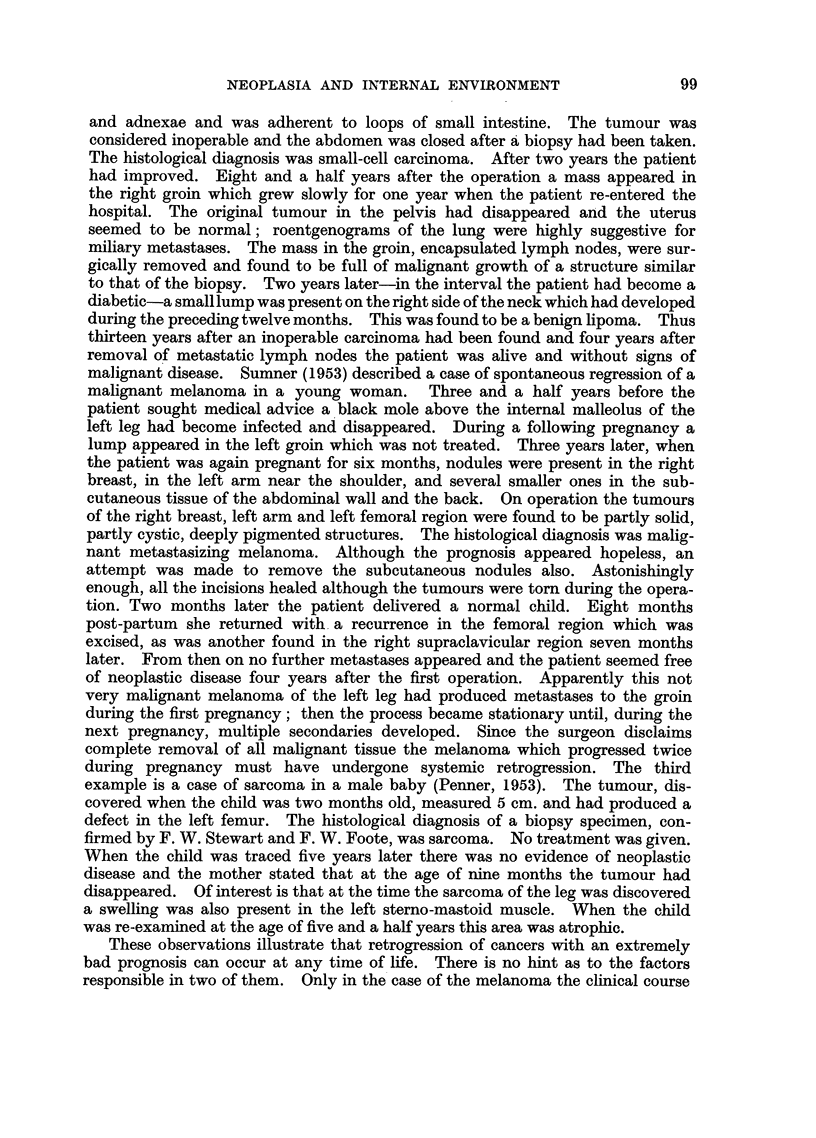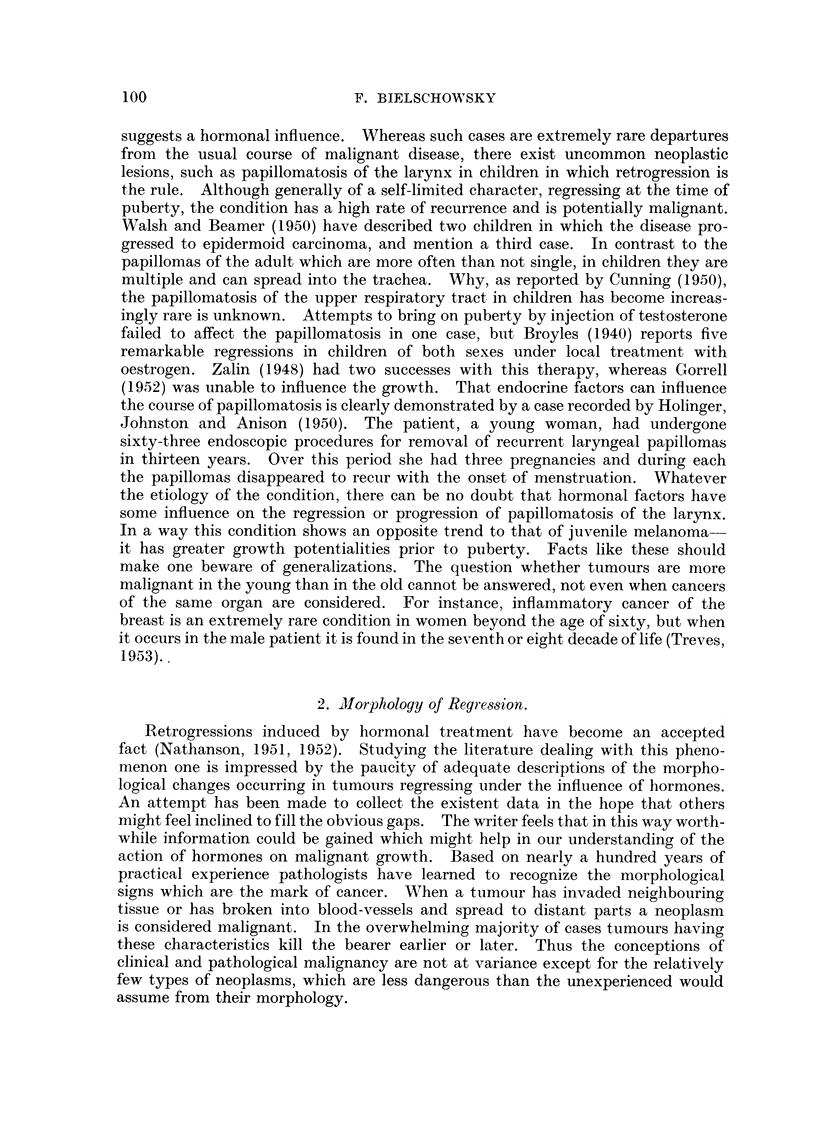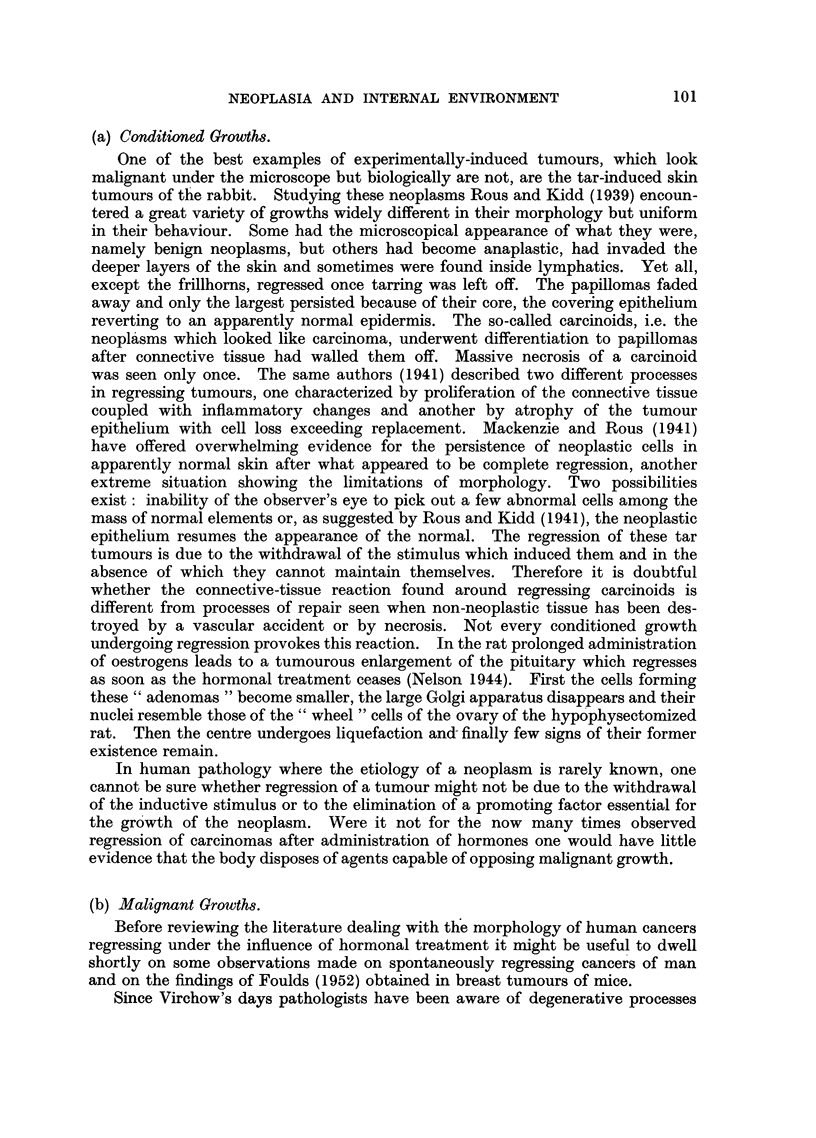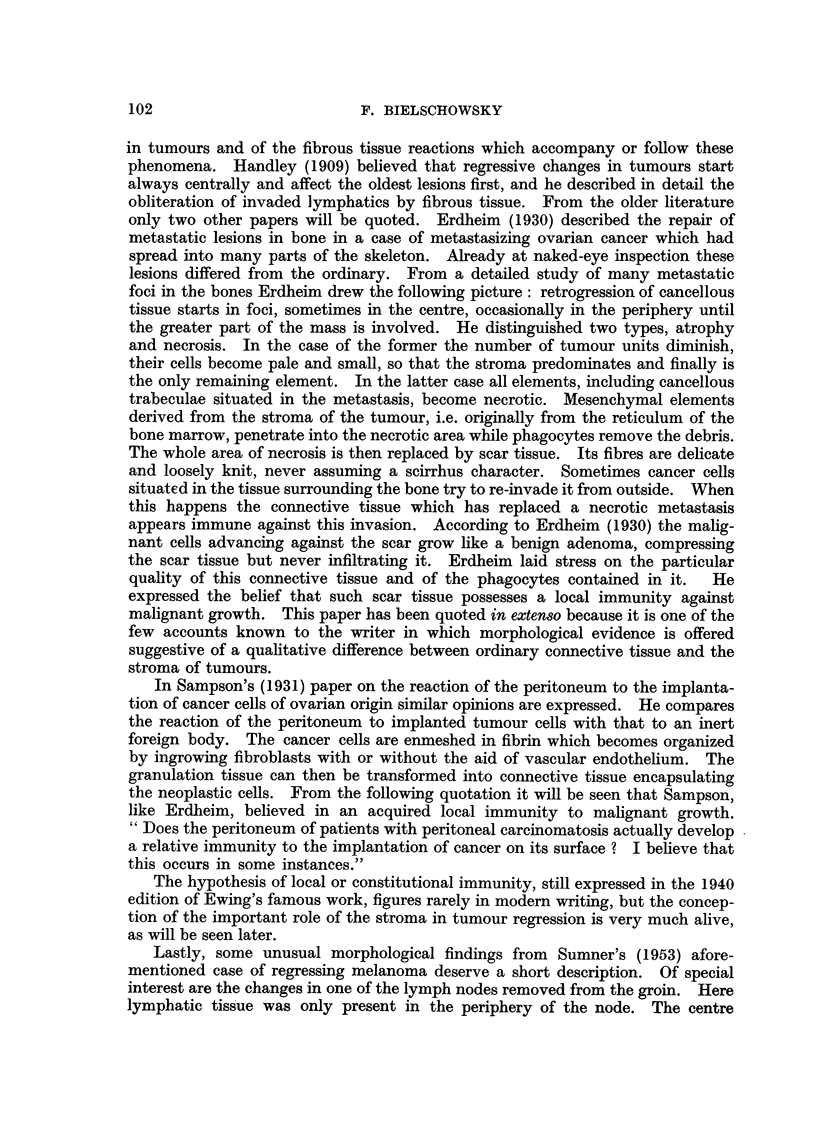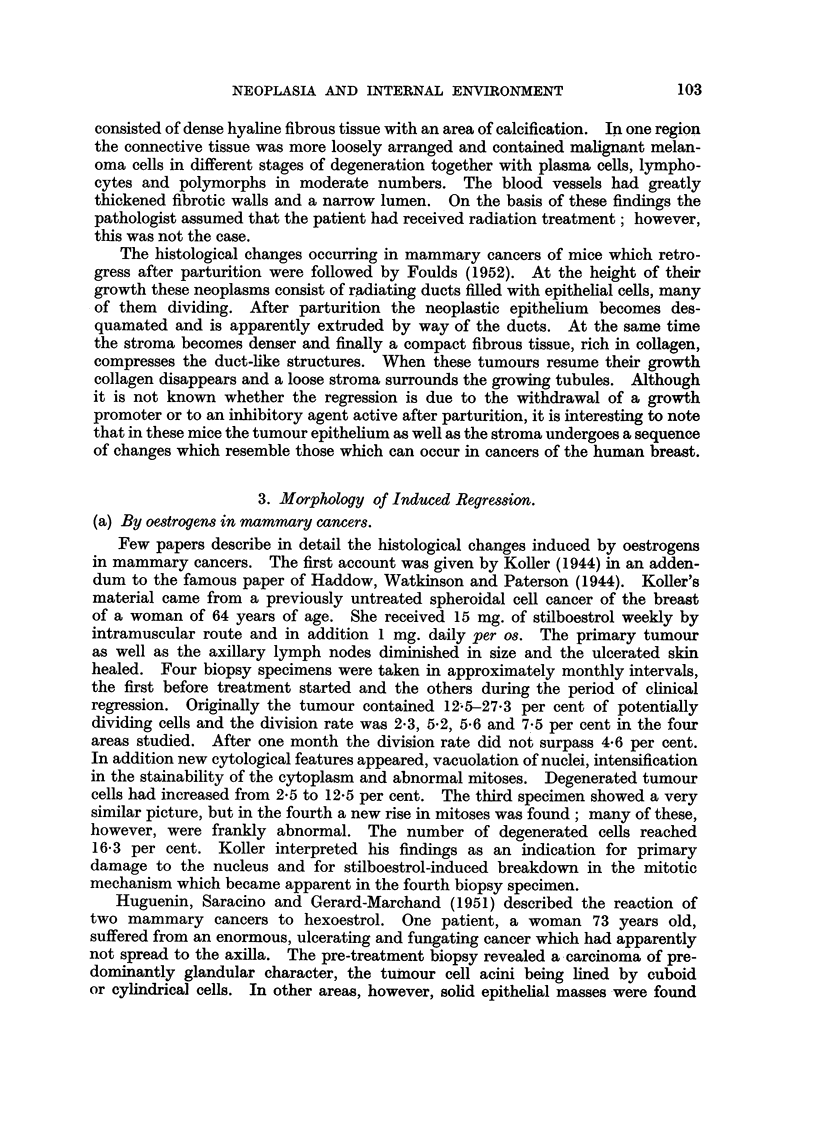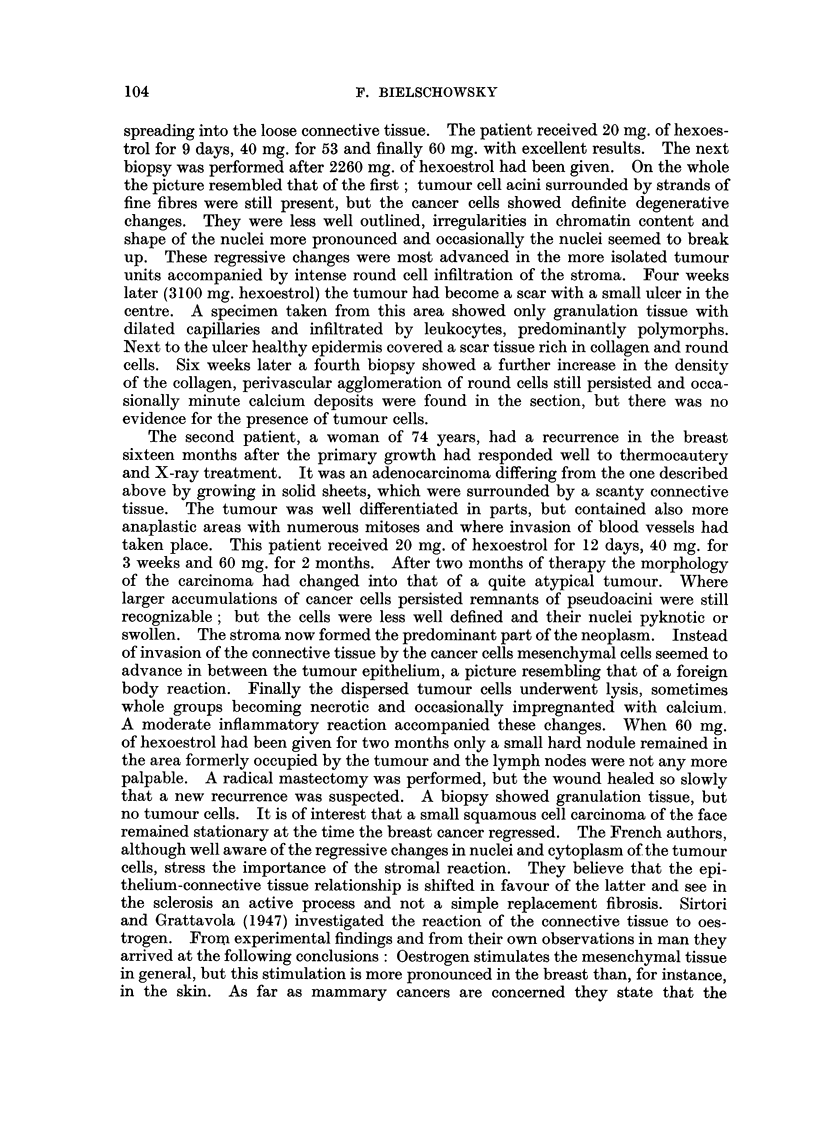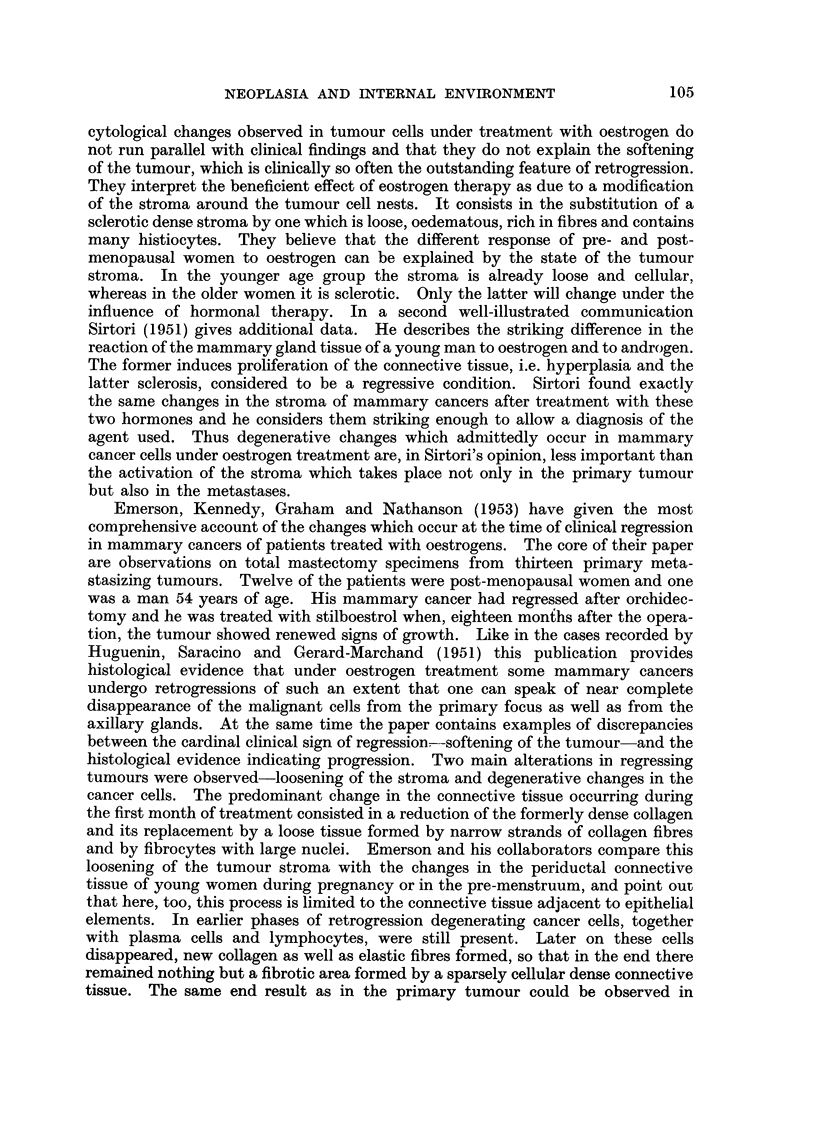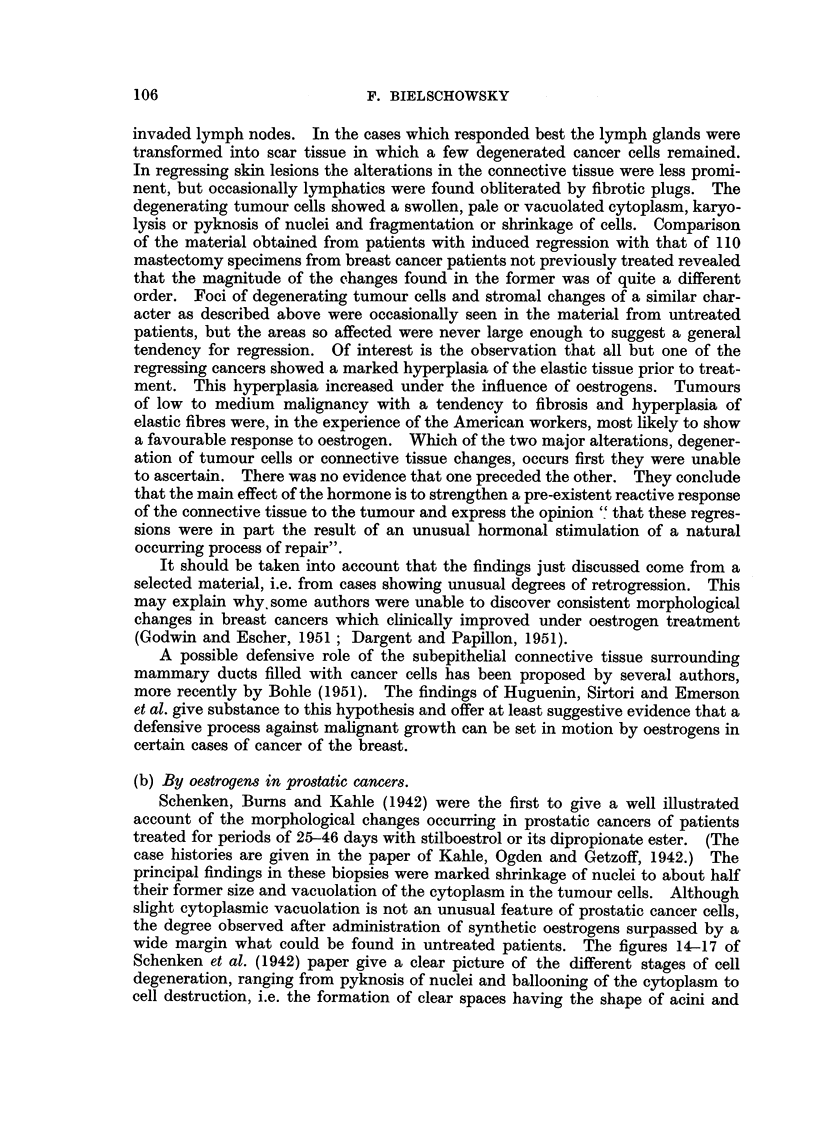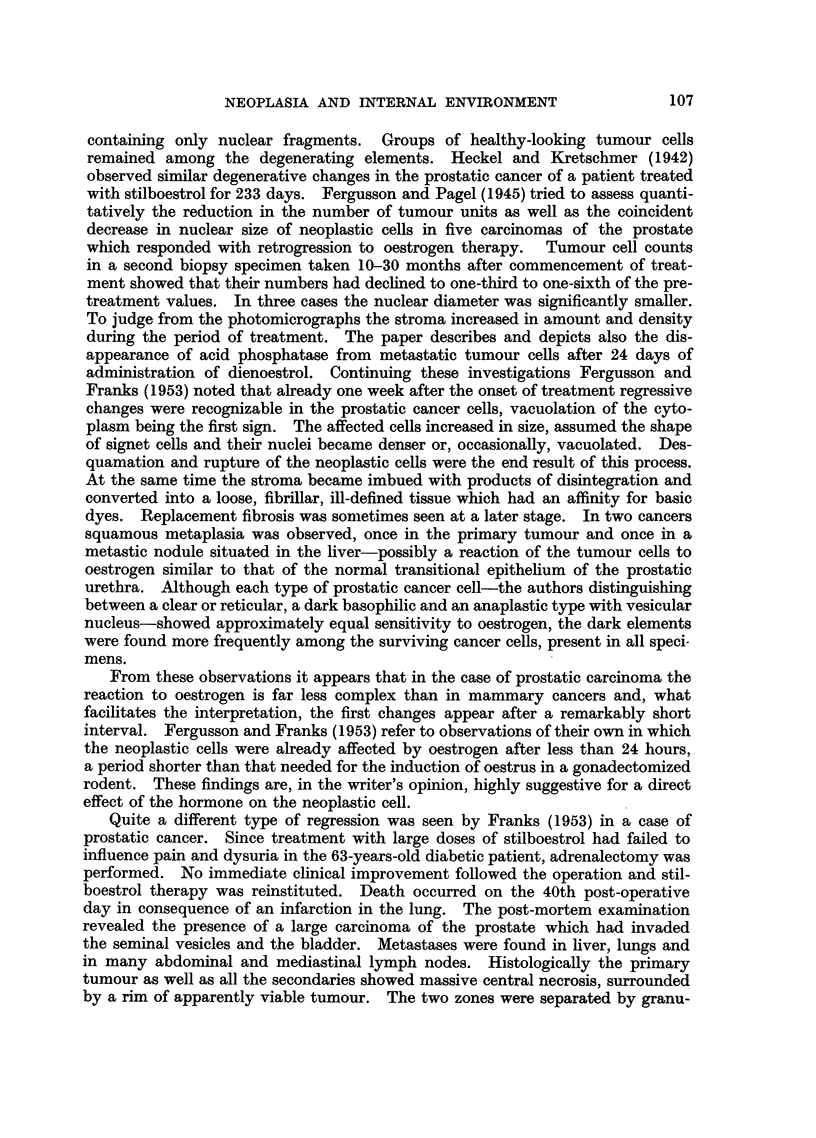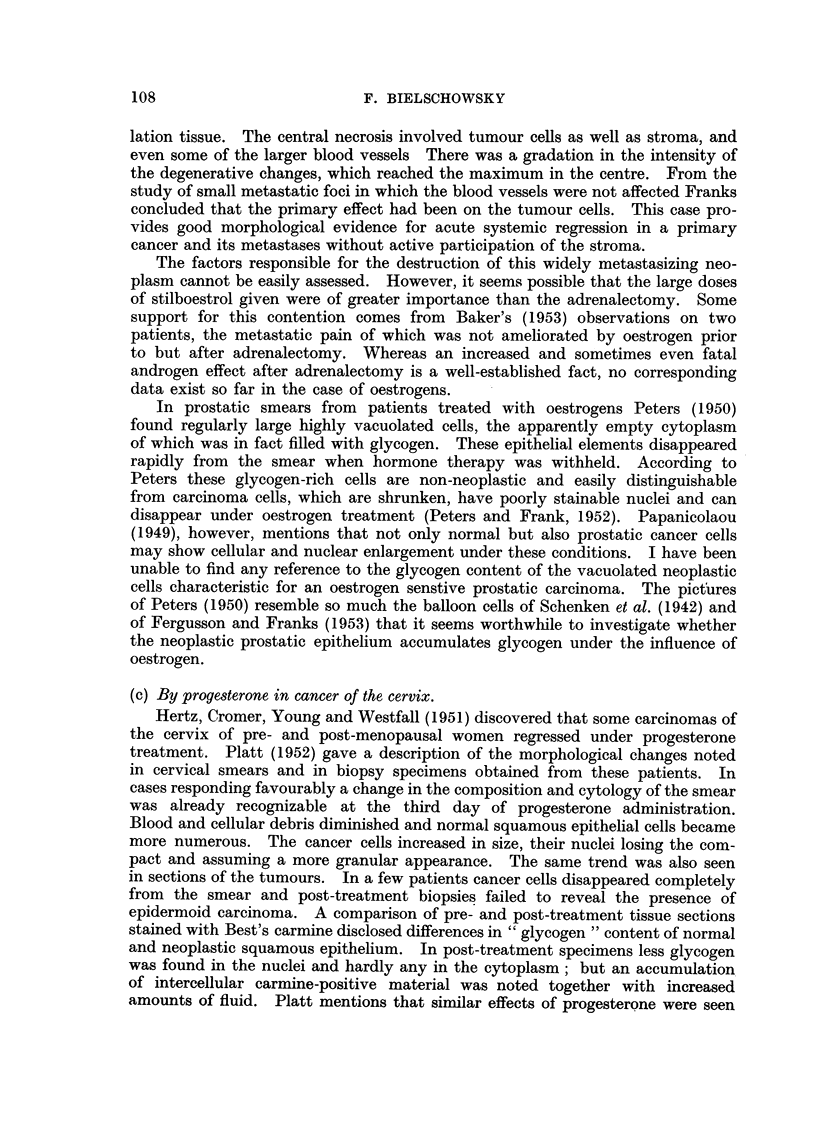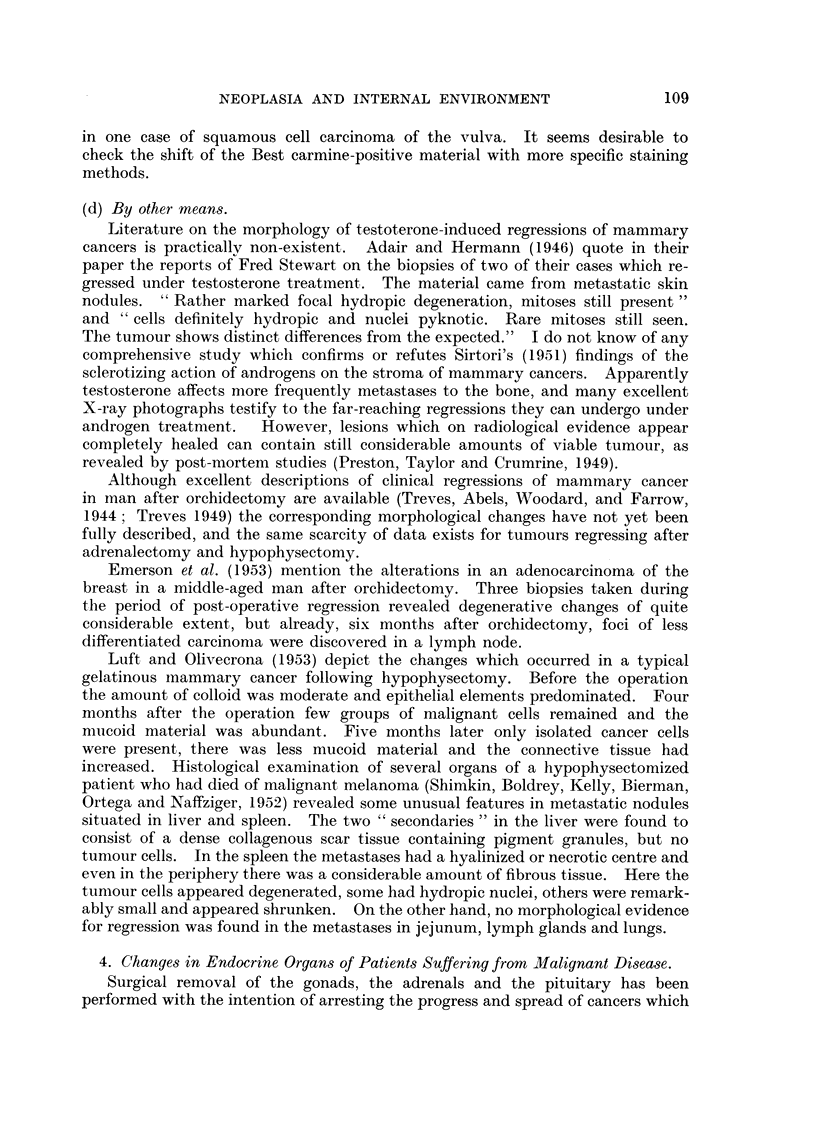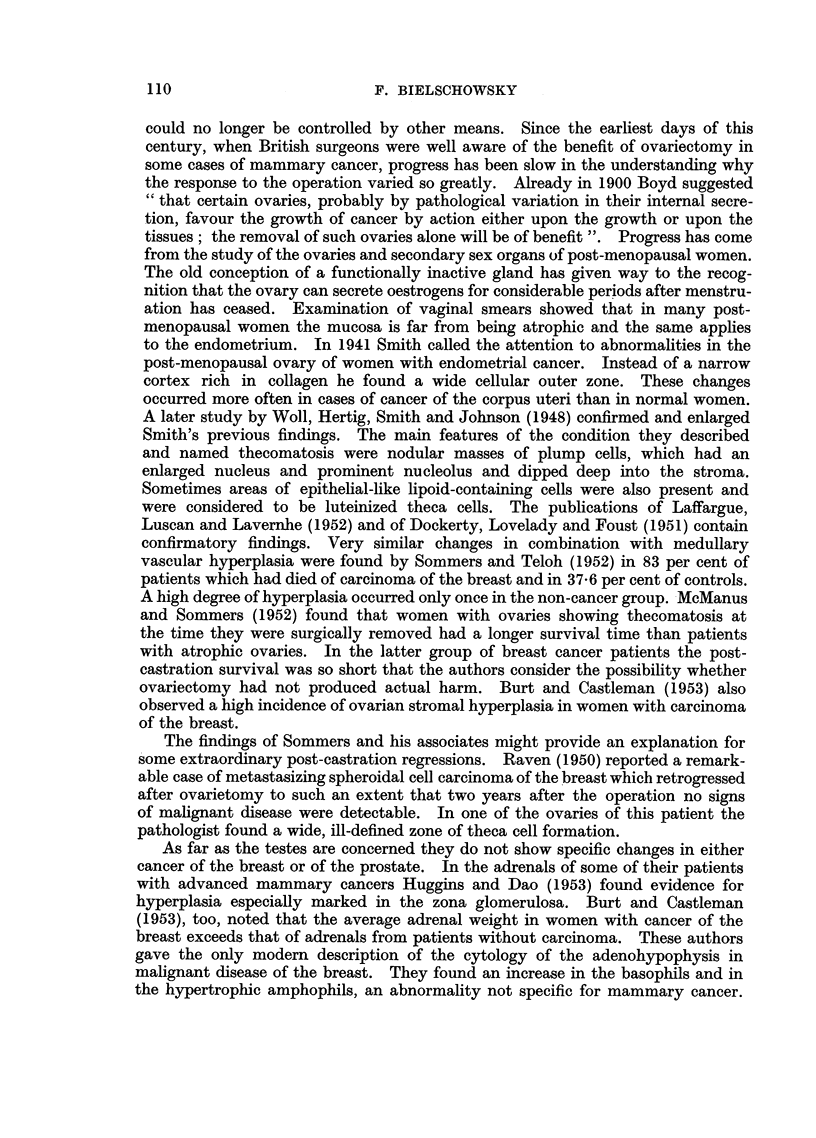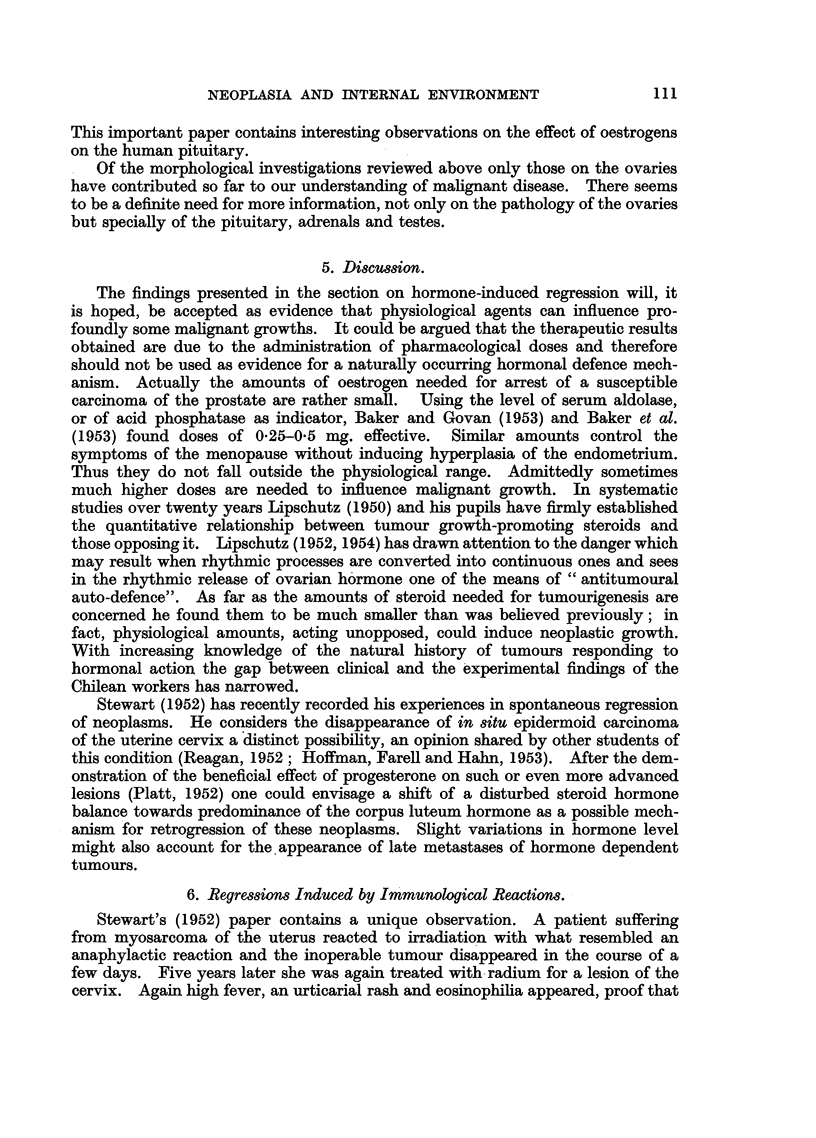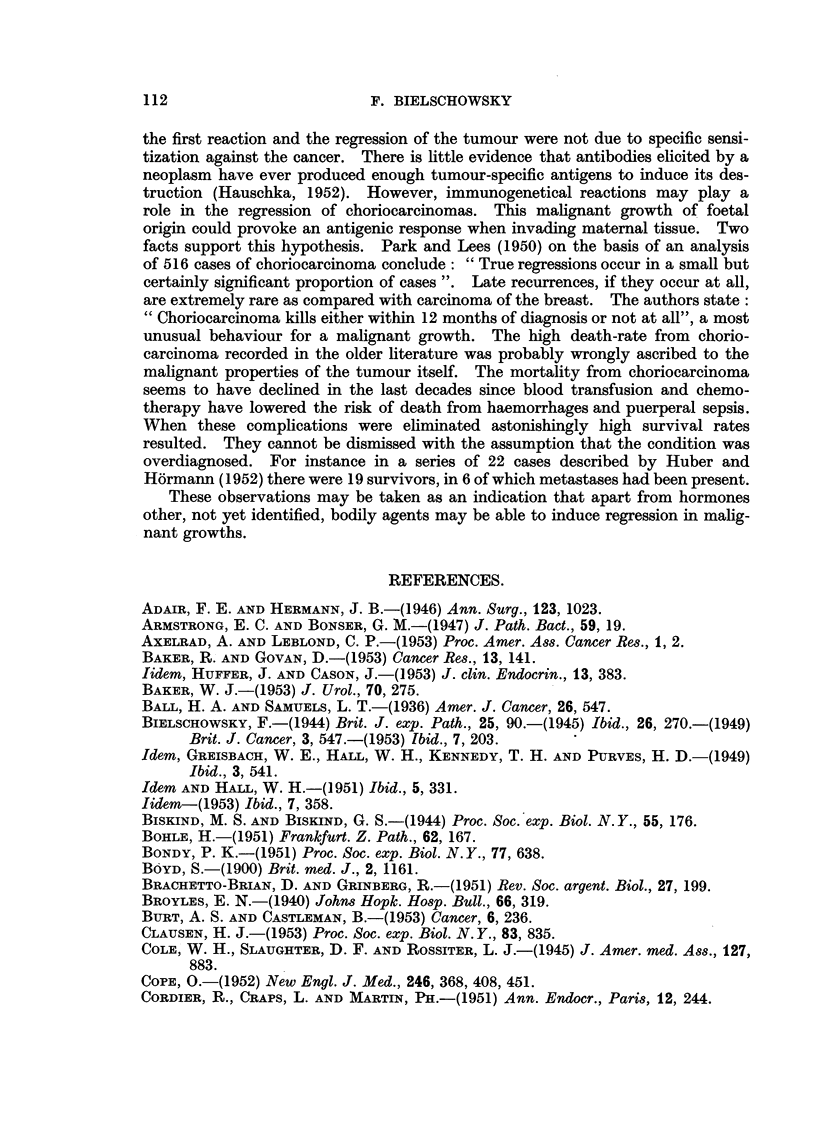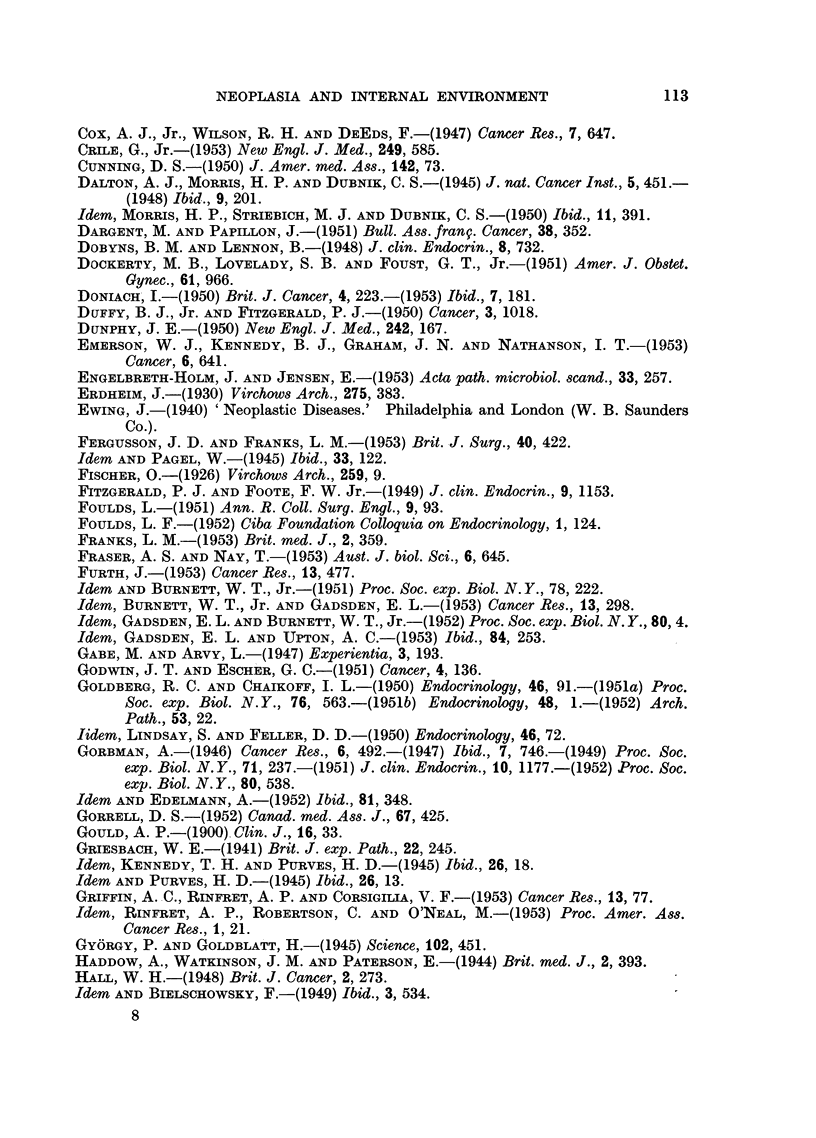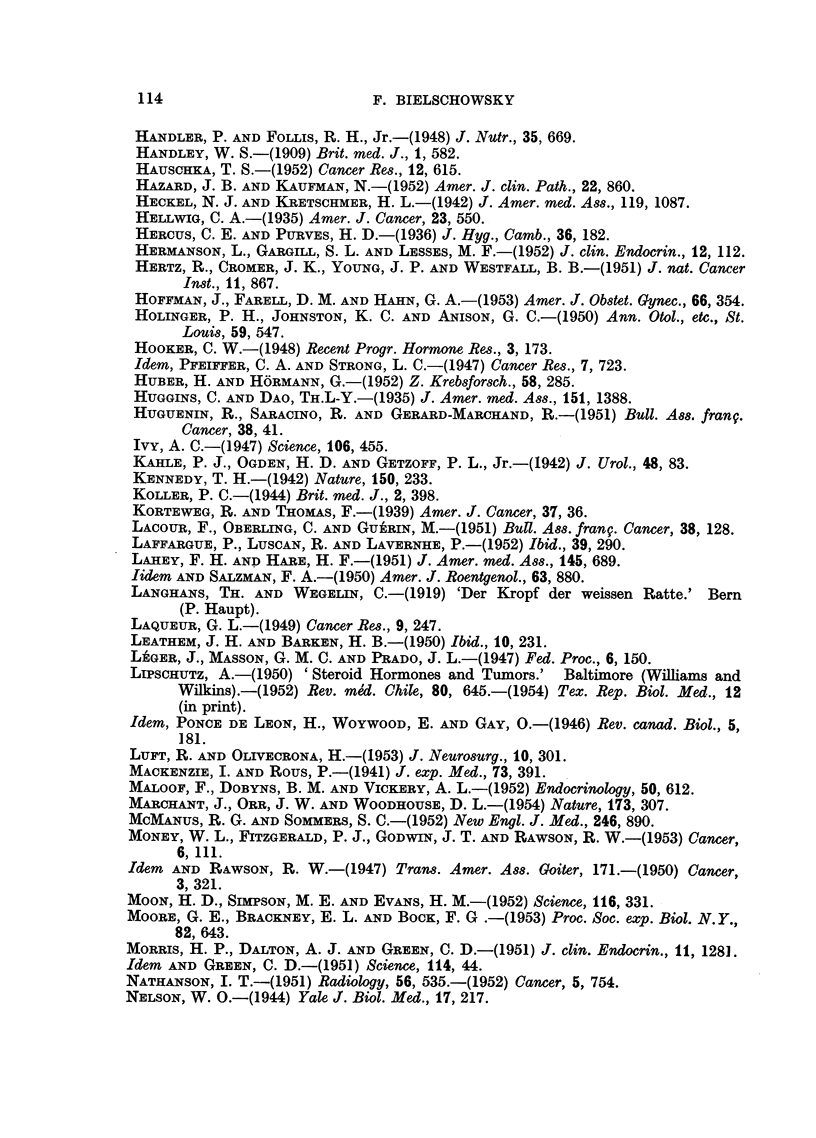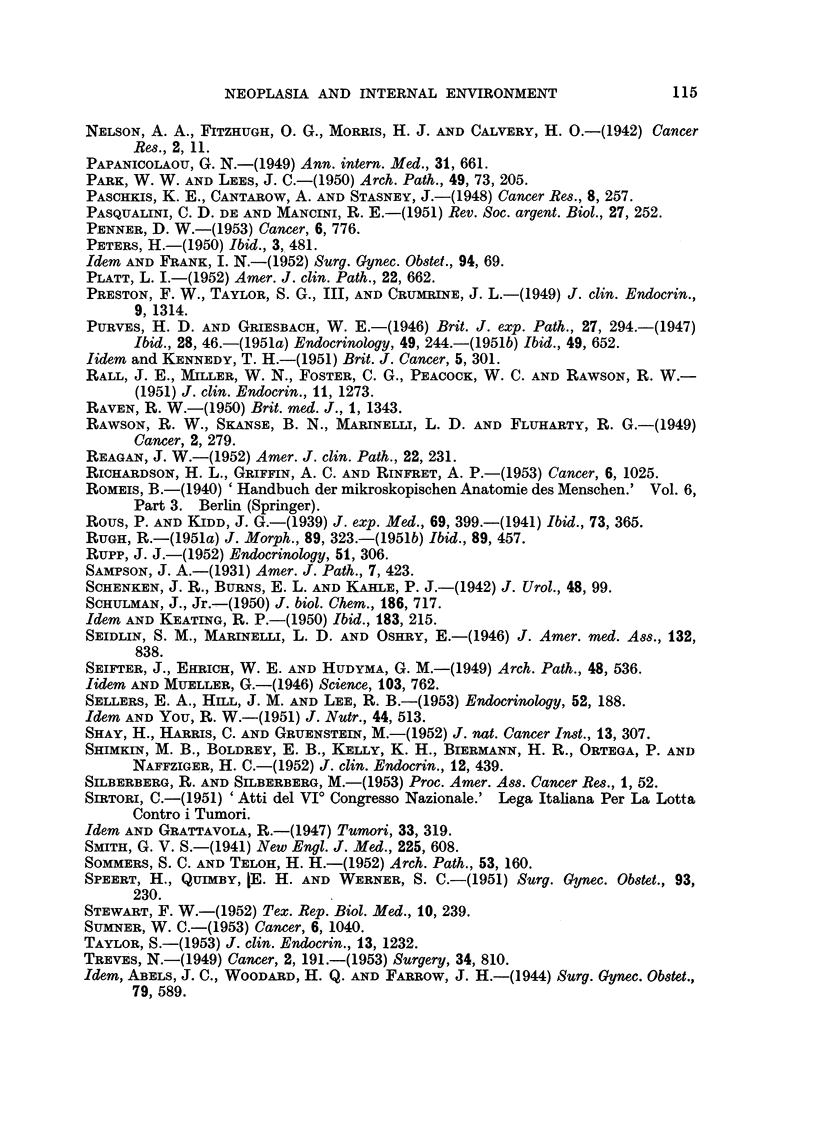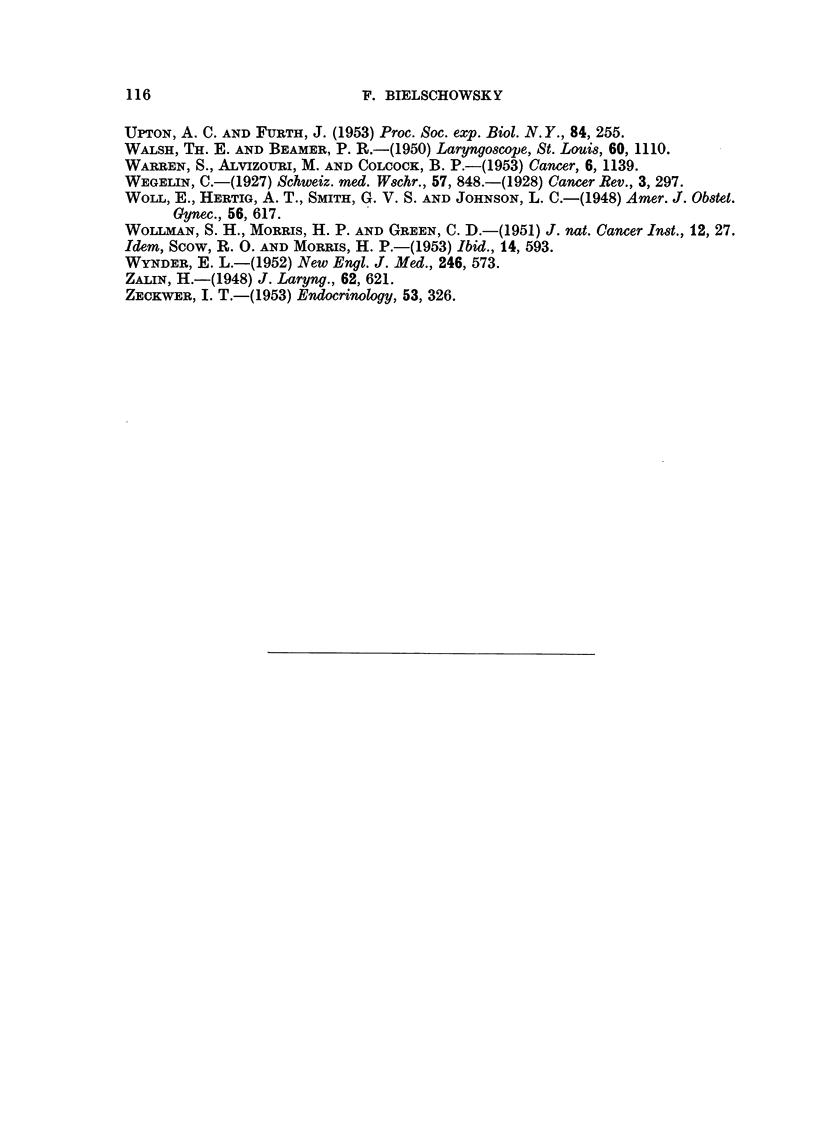# Neoplasia and Internal Environment

**DOI:** 10.1038/bjc.1955.7

**Published:** 1955-03

**Authors:** F. Bielschowsky


### NEOPLASIA AND INTERNAL ENVIRONMENT.

F. BIELSCHOWSKY.

*Hugh Adam Cancer Research Department of the Medical School and the New Zealand Branch of the British Empire Cancer Campaign, University of Otago, Dunedin, New Zealand.*

Received for publication January 1, 1955.

CONTENTS.

I. INTRODUCTION

II. THE ROLE OF THYROID AND PITUITARY HORMONES IN THE INITIATION OF CHEMICALLY INDUCED TUMOURS

III. THE ROLE OF THYROID HORMONE DEFICIENCY IN TUMOURIGENESIS IN THYROID AND PITUITARY
1. Thyroid Tumours Induced by Goitrogen in Rats
2. Thyroid Tumours Induced by Goitrogen in Mice
3. Thyroid Tumours Induced by $I^{131}$ in Rats
4. Thyroid Tumours Induced by $I^{131}$ in Mice
5. Thyroid Tumours Due to Iodine Deficiency
6. Thyroid Tumours in Intraspleenic Grafts
7. Functional Activity of Thyroid Tumours
8. Pituitary Tumours
9. Cancers of the Human Thyroid
10. Experimental Thyroid Tumours, an Example for Responsive Tumours

IV. RETROGRESSION OF TUMOURS
1. Spontaneous Regressions
2. Morphology of Spontaneous Regression
   (*a*) Conditioned Growths
   (*b*) Malignant Growths
3. Morphology of Induced Regression
   (*a*) By Oestrogens in Mammary Cancers
   (*b*) By Oestrogens in Prostatic Cancers
   (*c*) By Progesterone in Cancer of the Cervix
   (*d*) By Other Means
4. Changes in Endocrine Organs of Patients Suffering from Malignant Disease
5. Discussion
6. Regression Induced by Immunological Reactions

REFERENCES

### I. INTRODUCTION.

DURING the last fifteen years it has been generally recognised that the growth of tumours depends not only on the intrinsic properties of neoplastic cells but also on the environment in which they develop. Of the morphogenetic factors active in post-embryonic life only the secretions of endocrine glands have been identified so far. When speaking of internal environment in connection with normal or neoplastic growth one refers to variations in hormone levels. As will be shown, the reaction to an exogenous carcinogenic agent can be profoundly altered by a hormonal deficiency. Variations in hormone level can be the essential factor for the development of some tumours and such changes can, in certain circumstances, lead to retrogression of a cancer. In Section II the recent investigations into the response of hypophysectomized or thyroidectomized rodents to chemical carcinogens will be discussed. In the third section the neoplasms found in thyroid hormone deficient rats and mice will be reviewed and their relationship to human thyroid cancers discussed. Section IV deals with retrogression of malignant growths with special emphasis on the morphology of human cancers regressing under the influence of changes in the hormonal environment.

### II. The Role of Hormones in the Initiation of Chemically Induced Tumours.

Many organs cannot maintain their size in the absence of hormones on which they depend for their normal development and function. Generally it is difficult to induce neoplastic growths in atrophic tissues such as the breast of a rodent ovariectomized early in life. Only with the aid of massive doses of some of the most powerful carcinogenic hydrocarbons a pathological growth response can be occasionally obtained (Shay, Harris and Gruenstein, 1952). In the experiments to be discussed this type of atrophy can be ruled out as a major factor responsible for the failure of the carcinogen to act in its usual manner.

Moon, Simpson and Evans (1952) implanted pellets of methycholanthrene into the gastrocnemius of 15 hypophysectomized rats and saw only 1 sarcoma during the 316 days of observation. Twelve rapidly progressing cancers developed in the 15 intact controls. Griffin, Rinfret and Corsigilia (1953) fed a diet containing 3-methyl-4-dimethylaminoazobenzene to 18 hypophysectomized rats of two different strains for periods up to 19 weeks. The only pathological change observed was a mild cirrhosis in the liver of one of them. Multiple hepatomas appeared already during the 14th week in the intact controls. It is astonishing that two animals in which approximately 25 per cent of the pituitary were still present showed no signs of neoplasia in the liver but only cirrhosis. In a later communication, Richardson, Griffin and Rinfret (1953) described the histology of 24 completely and 2 incompletely hypophysectomized rats treated with the same carcinogenic azo dye. The livers of the former were normal in every respect but those of the latter showed the usual cirrhosis, proliferation of bile ducts, fatty changes and beginning neoplastic growth. Study of the adrenal glands of the experimental animals led the authors to suggest that "some factor elaborated by the pituitary is essential for adrenal-lipoid maintenance and subsequent cancer formation". However, their preliminary results with crude gonadotrophin fractions are difficult to reconcile with this contention (Griffin, Rinfret, Robertson and O'Neal, 1953).

Bielschowsky and Hall (1953) gave an account of the tumours which can be induced in thyroidectomized rats with 2-aminofluorene (AF) or its monoacetyl derivative (AAF). Only the results obtained with the amine will be quoted in detail. No liver tumour was found in 20 completely thyroidectomized rats, whereas 6 of the 10 partially thyroidectomized animals and 16 of the 18 intact controls had hepatomas. Reduction in the incidence of liver tumours induced by AAF in rats treated with thiouracil had been reported by Paschkis, Cantarow and Stasney (1948) and by Leathem and Barken (1950). The Philadelphia group found that under the influence of the goitrogen the percentage of hepatomas in males dropped from 83·6 to 20·2 and in the females from 46 per cent to zero. Leatham and Barken repeated this experiment with pair-fed rats and thus eliminated the possible effect of reduced food intake. After 8 months 50 per cent of the animals receiving the carcinogen alone had liver cancers and the rest benign cystic cholangiomas. Only one of the 8 males receiving AAF and thiouracil developed a hepatoma, but 6 had cystic lesions in the liver. Paschkis, Cantarow and Stasney (1948) included in their paper an experiment in which para-dimethylaminoazobenzene was used as carcinogen. In the males thiouracil did not modify the action of the azo dye, but it lowered the incidence of liver tumours in the females from 88 to 46 per cent. There exists therefore evidence that thyroid hormone deficiency modifies the response of the liver cells against AF and AAF and, perhaps, against butter yellow. In the writer's experience the degree of thyroxine deficiency is crucial for the outcome of the experiment. Animals, the pituitary of which still contained acidophilic cells in appreciable numbers, always showed the typical liver lesions which follow the administration of the aromatic amine. Rats with pituitaries void of acidophils, i.e. animals in which only traces of thyroxine could still be present in the body, had livers free of neoplastic changes. It should be kept in mind that a severe degree of thyroxine deficiency implies lack of growth hormone. The endocrine imbalance is rather complex since adrenals and gonads too are affected by complete thyroidectomy. However, gonadectomy gives only a very slight protection against the hepatoma-inducing action of AAF. From the studies on tumour induction by AF and AAF in thyroxine deficient animals it became evident that the protection given by thyroidectomy was restricted to the liver, whereas the susceptibility of the sebaceous glands of the meatus acousticus externus was not affected and that of the retrobulbar glands was even increased.

Richardson, Griffin and Rinfret (1953), as well as the writer, were impressed by the absence of the expected signs of liver damage in the hypophysectomized or thyroidectomized rats treated with carcinogens which have a predelection for the liver. The amounts of the carcinogenic agents given in these experiments always induce liver damage in intact animals. In my experiments the only abnormality noted was the occasional occurrence of highly vacuolated liver cells. It is known that thyroxine deficiency modifies the reaction of the liver to dietary injury (György and Goldblatt, 1945 ; Handler and Follis, 1948 ; Sellers and You, 1951). Léger, Masson and Prado (1947) observed that removal of the thyroid increased the resistance of the rat to the egg-white reaction. No satisfactory explanation can be offered at present as to the mechanism by which thyroxine deficiency protects the liver against injury of widely different etiology. The results obtained with carcinogenic agents are in line with the old conception of a causal connection between liver cell damage and hepatoma formation, which is further strengthened by the observation that thyroidectomy performed once AAF has produced liver cell damage, does not arrest the development of neoplastic growth.

The skin seems to be an organ in which hormone deficiency does not greatly modify the reaction to chemical carcinogens. Korteweg and Thomas (1939) studied the action of 3,4-benzpyrene on the skin of hypophysectomized mice. In one experiment the interval between operation and the start of painting the skin was 4 weeks, in another 5–7-months-old animals were used hypophysectomized at the age of 6 weeks. Skin tumours developed in all the control animals, papillomas appearing 44–100 days and carcinomas 93–104 days after onset of treatment. All of the 14 hypophysectomized mice still alive when the first papilloma was found on the 105th day of the experiment developed tumours of the skin; ten of them became cancerous between the 121st and 190th day. Although carcinogenesis was retarded in the hypophysectomized mice, the response of their skin to benzpyrene was qualitatively the same. Hall and Bielschowsky (unpublished results) repeated the work of Korteweg and Thomas (1939) using methylcholanthrene as carcinogen and restricting the application of this compound to 23 paintings. Our results are in excellent agreement with those of the Dutch workers. Benign and malignant tumours of the epidermis appeared after the administration of methylcholanthrene had been terminated in completely hypophysectomized mice showing the typical atrophy of the gonads and all the other morphological signs of pituitary deficiency. The neoplasms, however, developed slower than in the controls. One melanoma was found in a completely hypophysectomized female.

The histology of the reaction of muscle and connective tissue to methylcholanthrene in the hypophysectomized rat has not yet been described. The few data available indicate that once a hydrocarbon has induced a sarcoma, hypophysectomy will only retard its growth (Ball and Samuels, 1936). In the case of the liver of thyroidectomized rats the carcinogen apparently fails to change normal into neoplastic cells, i.e. the initiation of the neoplastic process is inhibited.

There is some suggestive evidence that other factors may be involved. Hypophysectomy seemingly does not affect the skin and its appendages to judge from the good condition of the fur and the rapid regrowth of clipped hairs in mice. Zeckwer (1953) noted that in the adrenalectomized rat regrowth of hair was even accelerated. Fraser and Nay (1953) observed a similar effect in ovariectomized mice, but inhibition of hair-growth towards the end of pregnancy, confirming earlier findings of the inhibitory action of elevated doses of oestrogen. In this connection recent results of Engelbreth-Holm and Jensen (1953) are relevant. The injection of growth hormone into mice failed to alter the incidence of skin tumours induced by a single application of 9,10-dimethyl, 1,2-benzanthracene. The only effect noted was a delayed appearance of neoplasms in the injected animals. From such observations and those of Korteweg and Thomas (1939) it seems that hormones have but little influence on the normal and neoplastic growth processes in the epidermis. On the other hand there is ample evidence that pituitary secretions can influence the growth of liver and of subcutaneous tissues as seen in acromegaly or after the injection of crude pituitary extracts.

### III. The Role of Thyroid Hormone Deficiency in Tumourigenesis in Thyroid and Pituitary.

During the last decade benign and malignant neoplasms of the thyroid of rats and mice have been obtained by various means. The conditions under which they occur are : (1) After administration of goitrogenic substances alone or in combination with carcinogens. (2) After administration of radio-iodine alone or in combination with goitrogens or acetlyaminofluorene. (3) After feeding an iodine deficient diet, and (4), possibly, after transplantation of thyroid grafts into the spleen.

### 1. *Thyroid Tumours Induced by Goitrogens in Rats.*

In the course of their systematic studies on experimental goitre Griesbach, Kennedy and Purves (1945) encountered neoplastic lesions in the thyroids of rats which had been kept for prolonged periods on regimens containing 45 per cent rape seed, the goitrogenic action of which had been discovered by Hercus and Purves (1936). After uninterrupted feeding of such diets single or multiple nodules of macroscopic size were present in the hyperplastic, hyperaemic glands, the first adenoma appearing in a rat receiving an iodine-poor rape seed ration for 8 months. The incidence of tumours rose to 80 per cent after 18 and to 100 per cent after 24 months. A supplement of iodine given to one group reduced the degree of hyperplasia in the thyroid, but had no influence on the number of thyroid tumours present in rats kept for 15 or more months on this regimen. None of the neoplastic lesions discovered in the thyroids of rats on the brassica-seed rations showed the morphological signs of malignancy, but one carcinoma of the thyroid developed in one of 10 animals receiving an iodine-poor control diet. The rats of the local colony were found to be more susceptible to the goitrogenic effect of rape seed than Wistar rats obtained from outside. Repeating this experiment with thiourea as goitrogenic agent Purves and Griesbach (1946) found 2 carcinomas in 30 rats, all of which had adenomatous thyroids. The diagnosis adenocarcinoma was based on the presence of multiple metastases in the lungs. In their next publication Purves and Griesbach (1947) amplified these findings and recorded the presence of 7 adenocarcinomas in 13 rats treated for 20 months with thiourea. Being familiar with the morphological changes occurring in the pituitary after administration of goitrogens, correctly interpreted by the New Zealand workers (Griesbach, 1941 ; Griesbach and Purves, 1945) as indicating stimulation of the thyrotrophic hormone (TSH) secreting basophils in response to thyroid hormone (TH) deficiency, Griesbach, Kennedy and Purves (1945) concluded that the thyroid tumours were the result of prolonged stimulation by TSH.

In 1944 the writer described the induction of tumours of the thyroid of rats by the simultaneous administration of a goitrogen, allylthiourea (Kennedy, 1942) and of a carcinogen, 2-acetylaminofluorene (AAF). The earliest neoplastic changes were seen in the thyroid of an animal killed on the 197th day of the experiment and one month later all rats had multiple adenomas. Two of the lesions were considered malignant because the neoplasms had invaded neighbouring structures. No adenomas were found in the control group treated with allylthiourea alone, nor have I ever observed thyroid tumours in rats with non-stimulated thyroids after treatment with AAF. However Armstrong and Bonser (1947) found two thyroid cancers and one colloid goitre in mice treated with this carcinogen and Cox, Wilson and DeEds (1947) 11 thyroid tumours in 84 rats. In continuation of his studies the writer (1945) tested the action of allylthiourea on the thyroid of rats treated previously for 25 weeks with AAF. After ten weeks of goitrogenic stimulation multiple adenomas were already present in the thyroids.

Of the 11 controls treated with allylthiourea alone for 6–14 weeks, 4 had minute adenomas, discovered by serial sectioning of the glands. Repetition of the experiment using piebald rats and reducing the administration of the carcinogen to 15 weeks, led again to the formation of multiple thyroid adenomas in most of the animals after 10 or more weeks of treatment with the goitrogen. No adenomas were found in the controls—proof that this strain was less sensitive to goitrogens than the Wistar rats used in previous experiments. In the same paper the fate of the single adenomas induced by allylthiourea in Wistar rats was described, the animals being killed 2–8 weeks after withdrawal of the goitrogen. The changes were identical with those observed by Griesbach *et al.* (1945) after the administration of physiological doses of thyroxine for 3 weeks. The tumour epithelium changed from tall cylindrical to a low cuboid type and colloid accumulated in the nodules. It is of interest that Purves and Griesbach (1946) could reproduce the thyroxine effect in a thyroid cancer and its metastases. When doses of thyroxine exceeding the daily requirement of the rat were administered, the thyroid tumours underwent the same involutionary changes which could be reversed by renewed administration of thiourea. Purves and Griesbach (1947) noted that under thyroid treatment colloid accumulated only in those benign or malignant neoplasms which showed a follicular structure.

Paschkis, Cantarow, and Stasney (1948) found in 93·6 per cent of their animals, treated with AAF and thiouracil, adenomata and in 14·8 per cent carcinomas of the thyroid. Of the rats on thiouracil alone 55 per cent developed benign thyroid tumours and only one neoplasm showed malignant changes. Hall (1948) repeated Bielschowsky's (1945) experiment giving 6 doses of AAF, a total of 15 mg., to each rat before methylthiouracil administration was started. Whether the stimulation of the thyroid by the goitrogen commenced immediately after the withdrawal of the carcinogen, or after an interval of 4–18 weeks, all pre-treated animals developed multiple adenomas which appeared earlier than the few single nodules found in the controls. Hall and Bielschowsky (1949) inquired whether such small amounts of AAF given prior to methylthiouracil administration played any role in the malignant change which can occur ultimately in glands after prolonged stimulation by a goitrogen. In the early stage of the experiment more numerous and larger adenomas were noted in the rats pre-treated with AAF, but all differences between them and the controls receiving methylthiouracil only disappeared after 18 months. Morphological signs of malignancy became recognizable in both groups at about the same time. On the other hand, adenomas induced by allylthiourea were not transformed into cancers when AAF was given subsequently to the withdrawal of the goitrogen. Doniach's (1950) findings are in agreement with Bielschowsky's (1945) and Hall's (1948) observations. In order to demonstrate that lack of thyroid hormone was the essential factor in the pathogenesis of these thyroid tumours Bielschowsky (1949) administered AAF to partially thyroidectomized rats. In the hyperplastic isthmus of these animals adenomas developed in the course of 18–37 weeks. Such lesions do not arise in 8 months in the isthmus of untreated partially thyroidectomized rats.

Thus AAF, even in small amounts, speeds up the development of adenomas of the thyroid of rats treated with goitrogens. At the same time it increases the frequency of the nodules. Large amounts of the carcinogen favour the rapid development of carcinomas.

Money and Rawson (1947) obtained only benign neoplasms of the thyroid by the administration of thiouracil to male rats for periods up to 18 months. Similar observations were recorded by Laqueur (1949).

In a second communication Money and Rawson (1950) give a detailed, well illustrated account of the histogenesis of these adenomata. They found no differences in the morphology of the tumours obtained in rats treated with thiouracil alone or in combination with dibenzanthracene. In one animal, hemithyroidectomized after 16 months of thiouracil treatment, and kept without goitrogen for an additional 9 months, thyroid adenomas were still recognizable in the remaining lobe which in all other respects had reassumed its normal appearance.

Sellers, Hill and Lee (1953) treated groups of 70 Wistar rats of both sexes for 15 months with propylthiouracil. Of the three experimental groups one received propylthiouracil alone, the two others the goitrogen supplemented by either NaI or dried thyroid powder. The body weight of rats receiving the goitrogen alone or in combination with iodide remained stationary, but the animals provided with thyroid powder gained weight. The latter had the largest thyroids and those receiving the iodide supplement the smallest. Neoplastic thyroid lesions developed in all groups. Four metastasizing tumours were found, one in the propylthiouracil group and two or three in the rats receiving additional thyroid powder. It is obvious from the iodine content of the thyroid glands at the time of post mortem as well as from the plasma iodine concentration that the amounts of thyroxine provided by the dried thyroid powder were insufficient to abolish completely the thyroid hormone deficiency induced by the goitrogen. This is confirmed by the morphology of the pituitaries which showed obvious signs of thyroxine deficiency in all three groups. In this connection some unpublished results of Hall might be mentioned. He succeeded in suppressing the appearance of hyper- or neoplastic changes in the thyroids of rats treated with thiouracil by supplying thyroid hormone in amounts corresponding to the daily requirements, i.e. 2·5 $\mu$g. of d-l thyroxine per 100 g. body weight. The maximal degree of hyperplasia found by Sellers, Hill and Lee (1953) in rats which received propylthiouracil and thyroid powder is best explained by the state of health of these animals. In my experience a high degree of thyroxine deficiency which does prevent body growth is not necessarily optimal for the induction of hyperplasia or neoplasia in the thyroid. Clausen (1953) in a long term experiment with thiourea found, apart from the usual adenomas, marked degenerative changes combined with extensive lymphocytic infiltration of the gland, a picture which bore some resemblance to struma lymphomatosa.

*bis*-4-Acetamino-phenyl-selenium dihydroxide seems to be a very potent goitrogen to judge from a report of Seifter, Ehrich, Hudyma and Mueller (1946). They fed diets containing 0·05 per cent of this compound to rats and already at the 105th day of the experiment multiple adenomas were present in the thyroids. When smaller amounts were fed over periods of 1–2 years the degree of hyperplasia observed was rather mild and limited to the interior of the gland ; only one adenoma was found. Benign liver tumours appeared in these rats, suggestive of a direct carcinogenic action of the compound (Seifter, Ehrich and Hudyma, 1949).

In the foregoing the terms adenoma and carcinoma have been used. That we are dealing not with simple nodular hypertrophy but with neoplasms is obvious from the behaviour of the nodules. They persist when the stimulus which induced them is no longer active. Their morphology, however, changes when the goitrogen is withdrawn or when thyroxine is injected in adequate amounts to suppress further stimulation by TSH. The epithelium of benign as well as of metastasizing tumours tends to assume under these conditions a shape akin to that of a resting thyroid. At the same time colloid accumulates in these structures as it does in the non-neoplastic part of the gland. These neoplasms vary considerably in size and morphology—some are minute structures comprising only a few acini, others are macroscopically visible nodules considerably larger than the thyroid of a normal rat. There exist two main types : solid and cystic ones. The solid micro- or macro-follicular adenomas stand out from the surrounding tissue by their densely packed cells with hyperchromatic nuclei ; they can contain some colloid when the rest of the gland contains none. In others the cells are arranged in the form of tubuli and in some the tumour epithelium grows in solid sheets, divided by strands of connective tissue, and forms acini only occasionally. The other type is characterized by papillomatous formations growing into cystic, colloid filled spaces. The degree of hyperaemia in these adenomas is remarkable, exceeding that of the rest of the gland. Large, blood-filled sinuses are found not only inside the nodules, but also often where they border the non-neoplastic tissue, and only a single layer of endothelium may separate the tumour tissue from the large vascular spaces. Regressive changes are not rare. Signs of recent or past haemorrhages are, perhaps, not as common as in human goitres of long standing. Thus one finds areas where haemosiderin-filled macrophages are lying in a dense connective tissue. Calcification is rarely seen. The great majority of the benign thyroid tumours have well-defined borders without possessing a true capsula. Once they have reached a certain size they compress invariably the surrounding tissue.

Most workers in this field have used the criteria of classical morbid anatomy for the diagnosis of malignancy : invasion of neighbouring structures, penetration of the capsule of the thyroid and metastases, most of which are blood-borne and therefore located in the lungs ; but spread to regional lymph nodes has also been observed. The incidence of metastases is not related to the structure of the primary tumour ; they occur with well-differentiated as well as with anaplastic growths. However, both the scirrhous cancers I have seen were invasive.

These morphological criteria of malignancy do not imply that the so-called carcinomas no longer need TSH for further growth. When transplanted they do not grow in normal animals, but they take in thyroxine-deficient rats, where they reach considerable size without killing the animal (Bielschowsky, Griesbach, Hall, Kennedy and Purves, 1949). When serially transplanted they can change into highly malignant neoplasms, which may kill normal rats within three weeks (Purves, Griesbach, Kennedy, 1951). Money and Rawson (1950), however, succeeded in transplanting into a normal recipient neoplastic tissue obtained from the thyroid of a rat which had been treated with thiouracil and dibenzanthracene. After a lag of 10 months the implant started to grow rapidly and invaded the surrounding tissue.

### 2. *Thyroid Tumours Induced by Goitrogens in Mice.*

At the same time as these investigations were pursued in the rat, workers in the National Cancer Institute Bethesda and elsewhere attacked the problem in mice. Dalton, Morris and Dubnik (1945) at first failed to find evidence for neoplasia in the hyperplastic thyroids of mice treated with thiourea, but later it became obvious that no fundamental difference existed between mice and rats in their response to goitrogens. In 1948 the same authors gave an account of the morphological changes seen in the organs of female C3H mice after long-term ingestion of thiourea and thiouracil. Both compounds induced a remarkable hyperplasia of the thyroid. After approximately three months pale staining " parafollicular " cells became increasingly numerous, but obvious neoplastic lesions were absent. Thyroid tissue which had broken through the gland's capsule or, in another case, was found between trachealis muscle and the tracheal epithelium, was considered non-neoplastic and its unusual location due to pressure and not to invasiveness. After twelve months of thiouracil treatment colloid-containing follicles increased in numbers and the nuclei of the cells lining them were smaller and more basophilic. The lungs of approximately 40 per cent of animals sacrificed after 362–464 days contained nodules of thyroid-tissue, remarkably like the acini of the hyperplastic thyroid gland. When C mice were treated with thiouracil (Dalton, Morris, Striebich and Dubnik, 1950) for ten or more months nodular structures appeared in the thyroid. They were of two types, one formed by large cells with vesicular nuclei and the other by smaller, well defined elements with more basophilic nuclei. Only one pulmonary metastasis was seen in this experiment. In view of the poor cytological evidence for a neoplastic change, Morris and Green (1951) studied the behaviour of serially transplanted " hyperplastic " thyroid tissue. The recipients were mice maintained on a thiouracil-containing diet. In one line, the original graft coming from a mouse treated with AAF and the goitrogen, autonomous behaviour, i.e. ability to grow in a normal host, appeared in the third generation, another line acquired independence in the ninth. These experiments prove that in the mouse the same sequence of changes occurs as in the rat and that ultimately " autonomous " cancers result. Morris, Dalton and Green (1951) have given a detailed, beautifully illustrated account of these transplantation experiments. Starting from a small adenomatous area found in the hyperplastic gland of a C3H mouse treated with thiouracil for 18 months, they obtained two tumour lines which differed morphologically and in their iodine metabolism ; but both reached the capacity to grow in normal hosts after the seventh transfer. One tumour was characterized by a follicular structure, in the other the cells grew in tortuous cords. Gorbman (1946, 1947) administered thiourea or thiouracil to mice of several inbred strains and to some hybrids. He found nodular structures in the thyroid after treatment for 220–300 days ; they developed from the ingrowth of tiny follicles or cellular cords into colloid cysts which were most prominent during the 180–200th day of the experiment. Thyroid tissue noted inside blood vessels and in the lungs was considered to be metastatic but not neoplastic. It is of interest that, in contrast to what has been observed in the rat, all these lesions disappeared rapidly after the withdrawal of the goitrogen and that neither AAF nor benzpyrene did enhance the action of the goitrogen.

### 3. *Thyroid Tumours Induced by Radio-iodine in Rats.*

In a series of papers Goldberg and Chaikoff and their associates have given a description of the changes in the rat's thyroid which follow treatment with radioactive iodine. Goldberg, Chaikoff, Lindsay and Feller (1950) tested four dose-levels of $I^{131}$, namely 18, 300, 525 and 875 microcuries. The injected animals were sacrificed after intervals ranging from 6 hours to 8 months. Only the long-term effects will be discussed, the two lower doses did not induce permanent changes. With the largest dose thyroid destruction was nearly complete and only in one animal glandular epithelium could be recognized 8 months later. With 525 $\mu$c. recovery from radiation damage took place to a certain extent and at the end of the experiment the thyroids of two animals were hyperplastic and resembled those after prolonged thiouracil treatment. Cells with ample eosinophilic cytoplasm and large irregular nuclei, likened to the so-called Hürthle cells of human pathology, were noted, the only atypical type of thyroid cells seen in this investigation. Money *et al.* (1953) found also " Hürthle cells " in the thyroids of rats treated with methylthiouracil, evidence that they owe their existence not necessarily to irradiation but probably to continued stimulation of the thyroid. That the presence of bizarre cells in irradiated thyroids is not interconnected with adenoma formation is evident from the findings of Maloof, Dobyns and Vickery (1952). They discovered only one adenoma in 500 rats treated with $I^{131}$ although gross cytological abnormalities were noted in many. In a second study Goldberg and Chaikoff (1951) administered doses of 400 $\mu$c. of $I^{131}$ and extended the period of observation to 18 months. They found in the thyroids of 2 rats multiple adenomas. Most were sharply defined, but two of the nodules blended with the surrounding tissue, were more cellular and richer in mitoses, and therefore suspect of beginning malignancy. In 1952 the same authors described the thyroid cancers present in rats treated with 400 $\mu$c. of $I^{131}$ and sacrificed 18–24 months later. Nine of 25 animals had neoplastic lesions in the thyroid. Seven of these tumours were considered malignant because five had metastasized and the other two had invaded adjacent tissue and blood vessels. In the strain of rats used by Goldberg and Chaikoff propylthiouracil failed to induce thyroid cancers, although 24 of 125 animals developed benign adenomas.

Doniach (1950) studied the effect of radio-iodine alone or in combination with methylthiouracil and/or AAF upon the thyroid. He worked with rats of the Lister strain, a colony in which single adenomas of the thyroid occur in 25 per cent of the animals at the age of 13 or more months. Two injections of 16 $\mu$c. each were given $5\frac{1}{2}$ months apart and the rats sacrificed $12–15\frac{1}{2}$ months after the administration of the first dose. Their thyroid glands were smaller than normally, but the majority contained two or more follicular adenomas. When the $I^{131}$ treatment was followed by administration of methylthiouracil multiple thyroid tumours developed and in one animal most of the tissue of the gland was replaced by adenomas, one of which had metastasized to one of the adrenals. The control group treated with methylthiouracil alone had large hyperplastic thyroids containing up to three benign neoplasms. The number of animals having tumours in their thyroids was the same in the two groups treated with radio-iodine or with methylthiouracil. Exposure to $I^{131}$ followed by goitrogenic stimulation led to the formation of neoplasms which were larger and more frequent than those induced by either agent, and on one occasion to malignant changes. Rats treated with AAF alone had normal or slightly stimulated glands and in 50 per cent of them 1–3 adenomas were present. Additional treatment with radioactive iodine did not increase tumour incidence in the thyroid. However, the simultaneous administration of the carcinogen and of methylthiouracil led to the formation of large nodular goitres and in two rats to an adenomatous replacement of most of the gland. The majority of the animals injected with $I^{131}$ and receiving in their drinking water AAF and methylthiouracil had moderately enlarged thyroids. In all of them adenomas were extremely numerous and one of these thyroid tumours had become malignant.

There is a striking parallelism between the results obtained in rats treated with $I^{131}$ and methylthiouracil and those obtained by the simultaneous administration of AAF and the goitrogen in so far as prolonged stimulation intensifies the carcinogenic effect of both $I^{131}$ and AAF.

In a more recent publication Doniach (1953) recorded the results of a second experiment in which the action of 30 $\mu$c. of $I^{131}$ alone or in combination with methylthiouracil was investigated and compared with the effects obtained with 5 $\mu$c. and 100 $\mu$c. respectively. The duration of the experiment was 15 months and the age of the rats at start 10 weeks. Of the three dose levels of $I^{131}$ tested 5 $\mu$c. and 30 $\mu$c. seemed equally effective as far as tumour formation was concerned. Both induced thyroid adenomas in 50 per cent of the injected animals, whereas the glands of the rats treated with 100 $\mu$c. did not contain neoplastic lesions. Still more impressive were the differences in the thyroids exposed to 5, 30 or 100 $\mu$c. when the glands were subsequently subjected to goitrogenic stimulation. All rats pretreated with 5 $\mu$c. had multiple benign adenomas of a size never reached by the neoplasms found in the thyroids of animals treated with methylthiouracil alone. In rats injected with 30 $\mu$c. and treated with the goitrogen afterwards the thyroid tumours were still larger and 5 of them obviously malignant. The thyroids of rats injected with 100 $\mu$c. failed to respond to goitrogenic stimulation and relatively few adenomas of moderate size developed.

The work of Doniach and of Goldberg and Chaikoff has established that amounts of $I^{131}$ which damage the thyroids but still allow them to respond to stimulation by TSH can be expected to induce neoplastic lesions in this organ. The tumours observed under these experimental conditions did not differ essentially from those seen after prolonged administration of goitrogens despite the presence of many bizarre elements in the irradiated glands. The accompanying changes in the pituitaries have been well described by Goldberg and Chaikoff (1950) and by Doniach (1953), and have been interpreted as being due to impaired thyroid function.

### 4. *Thryoid Tumours Induced by Radio-iodine in Mice.*

Gorbman (1949) using a wide range of doses of radio-iodine failed to detect any neoplastic changes in thyroids of mice injected at the age of 6–9 weeks. Speert, Quimby and Werner (1951) injected 200 $\mu$c. of $I^{131}$ into pregnant mice during the last three days of gestation and studied its effect on the offspring. There was evidence of thyroid damage early in life, but when the animals were 5 months old the glands had recovered and took up $I^{131}$ in normal quantities. Four to seven months later the thyroids were found to have a fibrotic centre, and to contain hyperplastic nodules with areas showing papillary ingrowth into follicles as well as colloid-filled cysts lined by a flat epithelium. Rugh (1951*a*) established that a major proportion of radio-iodine injected into a nursing mouse is excreted with the milk and therefore affects the suckling offspring. In a second communication (1951*b*) he described the changes which occur in the thyroids of mice nursed by mothers which had been injected on the 3rd day of lactation with doses of 3–20 $\mu$c. of $I^{131}$ per g. body weight. No tumours were found in the thyroid of the offspring 10 months later.

### 5. *Thyroid Tumours Due to Iodine Deficiency.*

The occurrence of nodular goitres in rat colonies kept in localities where goitre is endemic was first noted in Switzerland by Langhans and Wegelin (1919), and Wegelin (1927) recorded later the presence of malignant lesions in such enlarged thyroids. A similar observation was recently made in New Zealand, another country with endemic goitre. In spring 1951 enlarged, hyperaemic thyroids were seen in a fair number of experimental and of untreated rats of our colony, a phenomenon not observed by the writer in a large number of autopsies carried our during the previous three years. The fact that all cases of " spontaneous " thyroid enlargment were found at the same time and the disappearance of these goitres after providing the animals with additional iodide in the drinking water suggested iodine deficiency as the aetiological factor (Bielschowsky, 1953). Only the tumours found in untreated rats will be considered. Although the tumours seen during the active phase of the disease were of fair size, only one was recognizable at naked-eye inspection. Some were macrofollicular, others tubular and some were undifferentiated. All but one were well-defined benign neoplasms, the exception being one of the less differentiated, solid tumours which seemed to invade the surrounding hyperplastic thyroid tissue. The neoplastic lesions discovered in the resting glands of two aged animals 3–7 months after supplementary iodide had been provided, were rich in colloid and resembled closely the cyst adenomas seen in thyroids of rats after cessation of goitrogen treatment. Axelrad and Leblond (1953) studied the induction of thyroid tumours in rats fed a low iodine diet alone or with the addition of AAF. After one year a high percentage of animals in both groups were found to have thyroid tumours, some of which showed histological evidence of malignancy. These observations are in line with those of Hellwig (1935), one of the earliest workers to obtain thyroid tumours by means of an iodine deficient diet.

### 6. *Thyroid Tumours in Intrasplenic Grafts.*

According to some European and South American workers intrasplenic transplantation of thyroid tissue after complete removal of the gland from the trachea leads to a hyperplasia of the graft (Gabe and Arvy, 1947). Brachetto-Brian and Grinberg (1951) as well as Lacour, Oberling and Guérin (1951) observed nodular hyperplasia in intrasplenic thyroid grafts 14–15 months after the operation, and Cordier, Craps and Martin (1951) recorded the appearance of one trabecular adenoma, the only observation of this kind. Neither Bondy (1951) nor Rupp (1952) found evidence for thyroid hormone deficiency in rats bearing thyroid grafts in their spleen, nor did they find histological signs of increased activity in the transplanted thyroid epithelium. Thus the Biskind technique, so successful in the case of ovary and testis, hardly ever leads to the development of thyroid tumours, a result to be expected because this method more often than not fails to induce a thyroid hormone deficiency. However, Pasqualini and Mancini (1951) discovered in the pituitary of rats bearing thyroid grafts in the spleen changes characteristic of partial thyroidectomy.

### 7. *Functional Activity of Thyroid Tumours.*

Doniach (1950) was the first to obtain evidence of functional activity in an experimental thyroid tumour. He found that 12 days after cessation of treatment with AAF and methylthiouracil a follicular adenoma took up $I^{131}$, although to a lesser extent than the surrounding tissue. Purves, Griesbach and Kennedy (1951) worked with a transplantable thyroid tumour growing in rats which received 0·01 per cent methylthiouracil in the drinking water. They found that the graft could concentrate $I^{131}$ and assimilate it into organic form, despite the presence of the goitrogen. From the study of the pituitaries of thyroidectomized rats bearing such transplants the conclusion was drawn that the graft secreted thyroid hormone. Well granulated acidophils, absent from the pituitary of rats lacking TH, reappeared in the adenohypophysis and became rather numerous ; the counts of basophilic cells gave values below those characteristic for complete thyroidectomy. These results are representative for transplanted tumours which can grow only in thyroxine deficient animals. An anaplastic thyroid tumour which could be propagated in normal rats was not functionally active. Wollman, Morris and Green (1951) published data on the $I^{131}$ uptake of four different lines of transplantable thyroid tumours and of the thyroid glands of their hosts. One of the grafts took up very small and two moderate amounts, but the fourth was functionally so active that it depressed the normal activity of the host's thyroid. This transplant incorporated more $I^{131}$ into thyroxin than the thyroid of the tumour-bearing mouse. Wollman, Scow and Morris (1953) furnished additional data on the iodine metabolism of 6 tumour lines three of which depended still on continued thyrotrophic stimulation for their growth. They found that the grafts of the three dependent tumours could perform both functions of the normal thyroid to various extents ; they could concentrate $I^{131}$ and bind it in organic form. Even in the functional most active tumour graft the latter function was more impaired than the former. Of the three independent lines one had lost all functional activity, but the other two could still incorporate iodide into organic binding. Attempts to alter the iodine metabolism of one independent tumour by treating the host with goitrogens failed. In contrast to what has been seen in some human cancers or in experimental tumours of the rat, the incorporation of $I^{131}$ into an organic molecule was nearly completely blocked by propylthiouracil and the capacity of the tumour to concentrate iodine remained unchanged. Of great interest are the investigations of Money, Fitzgerald, Godwin and Rawson (1953), who studied $I^{131}$ uptake by the thyroids of rats kept for up to 700 days on a thiouracil containing diet. Twenty-four hours before autopsy the animals were injected intraperitoneally with 5 $\mu$c. of $I^{131}$. Radio-autographs of paraffin sections of the adenomatous goitres revealed the presence of areas rich in $I^{131}$. Usually in rats treated with a goitrogen the hyperplastic thyroid tissue takes up less iodine than normal thyroid tissue, and most of it is found in the inorganic fraction. The fact that the $I^{131}$ was not lost during the preparation of the sections suggested that iodine had been incorporated into an organic molecule. It was located in atypical follicles and in adenomas, whereas the non-neoplastic tissue seemed free of radio-activity. Thus a new type of cell had apparently come into existence which could accumulate and bind in organic form $I^{131}$ in the presence of thiouracil. Three possibilities exist : either the new tissue was no longer affected by concentrations of goitrogen which blocked thyroxine synthesis in non-neoplastic follicles or it had acquired the ability to incorporate iodine into organic molecules by new pathways. In this case the end-product of the synthetic process would probably be different from that formed normally. The third possibility would be a reduced ability of the neoplastic tissue to concentrate the goitrogen, whereas the hyperplastic follicles still performed this function. As shown by Schulman and Keating (1950) and by Schulman (1950) the thyroid can achieve a thirtyfold concentration of thiourea.

The work on the iodine metabolism of experimental thyroid tumours and their grafts has furnished results which are in good agreement with those obtained in human cancers of the thyroid. Rawson, Skanse, Marinelli and Fluharty (1949) noted that thiouracil interfered less with the uptake of iodine by functional carcinomas than by normal thyroid tissue. In man as in rodents the well differentiated tumours are prone to concentrate the isotope (Dobyns and Lennon, 1948 ; Fitzgerald and Foote, 1949). In most instances the amounts trapped are far below those taken up by the normal thyroid. However, occasionally considerable quantities may be retained, as for instance by the rare thyroid cancers which produce signs of hyperthyroidism (Seidlin, Marinelli and Oshry, 1946).

Many non-functional metastasizing tumours will take up radio-iodine after total thyroidectomy or in response to injections of TSH or to the administration of a goitrogen. After surgical removal of the thyroid Rall, Miller, Foster, Peacock and Rawson (1951) noted an increased uptake by the metastases in 21 of 30 patients under the influence of thiouracil. Of great theoretical interest is their observation that some of these tumours acquired rather abruptly the capacity of collecting $I^{131}$, whereas others did so only gradually. Since a follicular structure seems to be the pre-requisite for the trapping of iodine these observations imply a change of the neoplasm towards differentiation.

### 8. *Pituitary Tumours.*

Enlarged or obviously neoplastic pituitaries have been observed by many workers studying the effects of long continued TH deficiency in rats or mice. It took rather a long time to establish the significance of these lesions because several obstacles militated against their proper assessment. In the rat they occur most frequently at an age when " spontaneous " pituitary tumours make their appearance. In addition they may even have some resemblance to the " spontaneous " tumours. Better understanding of the normal histology of the rat's pituitary and the introduction of new staining techniques have helped to trace their development from the TSH producing basophils of the adenohypophysis. The best evidence, however, comes from the study of the pituitary tumours which can be readily induced in several strains of mice by $I^{131}$ given in thyroid destroying amounts. They were discovered by Gorbman, and Furth and his associates established their nature. Gorbman (1949) found that doses of 80–300 $\mu$c. of radio-iodine led to a progressive enlargment of the pituitaries, well recognizable already 150 days after the injection. Large tumours were present 100 or more days later, their size being related to the dose given, the largest tumours appearing in the group treated with 300 $\mu$c. Mice kept on a thiouracil containing diet for 400 days had enlarged pituitaries, but no neoplasms in the adenohypophysis. Whereas 200 $\mu$c. $I^{131}$ were needed for near complete destruction of thyroids of mice on a well balanced diet, 30 $\mu$c. achieved nearly the same effect in animals previously fed a low iodine ration ; but such mice did not develop pituitary tumours (Gorbman, 1951). Since implantation of thyroid tissue and to a lesser degree administration of thyroxine prevented pituitary enlargement as well as tumour formation in mice receiving effective amounts of $I^{131}$, Gorbman (1952) came to the conclusion that ionizing radiations in combination with TH deficiency were the essential factors for the development of these neoplasms. The failure of small but thyroid-destroying doses of $I^{131}$ to induce neoplastic growth in the pituitary was confirmed by Gorbman and Edelman (1952). The combination of whole body irradiation (545 r) and of radio-thyroidectomy by 30 $\mu$c. $I^{131}$ was found to be effective, whereas each agent alone was not carcinogenic for the pituitary. It might be pointed out that the tumours of the adenohypophysis developing after exposure to $I^{131}$ are not the only type which can be induced in the pituitary by radiations. Another entirely different type, has been found by Furth and his collaborators (Furth, Gadsden and Upton, 1953 ; Upton and Furth, 1953), in mice exposed to atomic detonations. Gorbman's results amplify observations of Goldberg and Chaikoff (1951). Having failed to induce pituitary tumours in rats with amounts of $I^{131}$ up to 875 $\mu$c., they treated mice with 600 $\mu$c. Only marked hyperplasia of basophilic cells in the anterior lobe resulted ; these cells appeared hypertrophic and showed increased mitotic activity. A supplement of desiccated thyroid prevented these changes.

From the experiments of Speert *et al* (1951) and of Rugh (1951) in which mice were exposed to $I^{131}$ three days prior to or after birth it is evident that total thyroid destruction is not essential for the subsequent development of pituitary tumours. These workers noted a considerable degree of anatomical and functional recovery of the thyroids, and although these glands were far from normal they must have secreted some TH to judge from the near normal weight reached by these mice. Nevertheless, a considerable number of them developed pituitary tumours 10–12 months after exposure to $I^{131}$. Recently Silberberg and Silberberg (1953) published data on the influence of genetic factors and on the enhancing effect of a high fat diet on the development of radio-iodine induced pituitary tumours.

Furth and Burnett (1951) transplanted pituitary tumours which had developed in mice treated with $I^{131}$ and found that these implants would not grow in normal mice, but in animals subjected previously to radio-thyroidectomy. Later Furth, Gadsden and Burnett (1952) obtained by serial transplantation tumour lines the growth of which was not any more dependent on the absence of TH. These autonomous grafts grew more rapidly than the dependent transplants, metastasized into lymph nodes and, what was most important, the presence of an intact thyroid in the host allowed the recognition of their secretory activity. Grafts of microscopic size already stimulated the thyroid of the host. When they enlarged they induced nodular goitres in due course. Assays of the hormone content (Furth, Burnett and Gadsden 1953), performed in Furth's own and three other laboratories, confirmed the high TSH content of the transplanted pituitary neoplasms. Of special interest were the assays of Evelyn Anderson, which indicated that no other hormones were present in significant amounts. Additional evidence for the TSH secretion of the transplants was furnished by the study of the iodine metabolism of grafted animals in which a marked tendency for iodine retention was noted.

The presence of pituitary tumours in mice (Moore, Brackney and Bock, 1953) and in rats treated with goitrogens for prolonged periods has been frequently observed (Griesbach, Kennedy and Purves, 1945 ; Seifter, Ehrich and Hudyma, 1949 ; Sellers *et al.*, 1953 ; Purves and Griesbach, 1951) and by Doniach (1953) in two rats treated with 100 $\mu$c. $I^{131}$. Only in the two last-mentioned publications these tumours are referred to as basophil adenomas, whereas most other authors classify them as chromophobic. Recently the writer (1953) described the occurrence of basophil adenomas in conjunction with nodular goitres in rats suffering from chronic iodine deficiency. A similar observation was recorded by Fischer (1926) who found in two aged rats neoplasms in thyroid as well as in pituitary. Her animals came from Wegelin's colony, in which goitre was endemic. The cells forming these basophil adenomas resembled strongly normal thyrotrophs in appearance and in their staining qualities. These cytological findings together with the fact that of all pituitary dependent organs only the thyroid was stimulated, was considered evidence of their TSH secreting activity. Since this hormone is formed by a special type of basophil, the thyrotroph of Purves and Griesbach (1951*a*, 1951*b*), these neoplasms were classified as basophilic adenomas, although in some " basophilia " was not a prominent feature. Apparently as long as the animals are deficient in TH these tumours do not store TSH, and only a few of their cells stain basophilic or with the PAS or Gomori's fuchsine-aldehyde reagent, a behaviour similar to that of the non-neoplastic thyrotrophs. Therefore it is debatable whether the name basophil adenoma is the most appropriate, the term TSH-secreting adenoma would be more precise. To call them chromophobic adenomas is misleading. A degranulated thyrotroph or $\beta$ cell to use Romeis's (1940) classification ought to be distinguished from a chromophobe or $\gamma$ cell.

To summarize : Thyroid hormone deficiency is the one factor common to all observations reviewed in this section. Whether this deficiency is due to an insufficient iodine content of the diet, or is induced by goitrogens through interference with the enzymatic mechanism by which the thyroid synthesizes TH, or to damage or destruction of the gland by ionizing radiations, the result is always the same. The pituitary reacts to the lowered thyroxine level with an increase of those basophils which secrete TSH, a process akin to compensatory hypertrophy. In the rat it takes a long time before this process leads to irreversible changes in the adeno-hypophysis and a tumour results. In the mouse radio-iodine, perhaps by a direct radiation effect on the pituitary, speeds up adenoma formation. The thyroid reacts to thyrotrophic stimulation with hyperplasia, which is first diffuse, then becomes nodular and finally neoplastic lesions appear which can show the morphological signs of malignancy. This sequence of events can be more easily demonstrated in the rat than in the mouse. When the life of the experimental thyroid tumours and the period of stimulation are prolonged by serial transplantation, carcinomas result which grow in normal animals, i.e. they become independent of the stimulus responsible for their initiation and development. The same phenomenon has been observed with the TSH secreting tumours of the mouse pituitary. Tumourigenesis in the rat's thyroid can be speeded up by chemical carcinogens and by ionizing radiations. As Doniach (1953) has pointed out, the danger of tumour development is greatest at a certain dose level, namely with amounts of $I^{131}$ which do not abolish the ability of the gland to react to TSH. Of importance is that all the neoplastic lesions, be it in pituitary or thyroid, can be prevented by supplying the animals with adequate amounts of TH.

### 9. *Cancers of the Human Thyroid.*

It is outside the scope of this contribution to review the vast literature on neoplastic lesions of the human thyroid. The conception of hyperplasia as first step to tumour formation in this organ seems to be generally accepted. Taylor

(1953) tracing the evolution of nodular goitre writes : " This series begins with a simple diffuse enlargement of the gland in a young patient and ends with the large multinodular goitre showing evidence of haemorrhage and calcification in the older individual ". Admittedly regression in human nodular goitre is a more frequent event than in the rat, and such regressions occur perhaps more often at a time when the process is still reversible, i.e. in the stage of nodular hyperplasia. Nevertheless, a certain number of nodules arising in hyperplastic glands become neoplastic and sometimes malignant. Wegelin (1928) states : " Where goitre is endemic, malignant tumours of the thyroid occur in greater numbers ". Statistical data (Wynder, 1952) show a remarkable decline in the death-rate from cancer of the thyroid since the introduction of iodized salt into the canton of Zurich in 1923, evidence for the etiological significance of iodine—and in consequence thyroid hormone deficiency in the causation of cancers of the thyroid. Ivy (1947) found in 2000 autopsies of adult dogs in Chicago an incidence of 89 per cent of goitres and of 1·6 per cent of metastasizing thyroid tumours before 1925. Two years later, after the introduction of iodized salt, both lesions had disappeared. Thus there exists a gratifying agreement between experimental and clinical findings. Still more satisfactory is that the type of thyroid cancer associated with endemic goitre seems preventable by a correction of the hormonal imbalance. However, as far as the human pituitary is concerned, there is hardly any evidence for tumour formation as a sequence to chronic thyroid hormone deficiency. From the foregoing it should not be assumed that the problem of thyroid cancer is a closed chapter. Especially, thyroid cancer in children and adolescents poses unsolved problems. Duffy and Fitzgerald (1950) found in the histories of 28 juvenile patients 10 in which the thymus had been irradiated between the 4th and 16th month of life. Whereas it is understandable why these neoplasms became manifest at the time of puberty, a period of increased growth rate of the gland, the etiological relationship between irradiation of the thymus and neoplasia in the thyroid is obscure. In a similar series of Warren, Alvizouri and Colcock (1953) neither irradiation of the thymus nor iodine deficiency played a significant etiological role. There is another type of carcinoma of the thyroid in which a history of previous goitre is frequently missing : the highly malignant anaplastic tumours found in elderly patients (Crile, 1953).

The question how often malignant changes occur in human nodular goitre has been answered differently and has been ably discussed by Cope (1952). Some surgeons are convinced of the great potential danger inherent in multinodular goitres and still more in the so-called solitary adenomas (Cole, Slaughter and Rossiter, 1945 ; Lahey, Hare and Salzman, 1950 ; Lahey and Hare, 1951). How erroneous the clinical impression of a solitary nodule can be has been pointed out by Hermanson, Gargill and Lesses (1952), who found nearly as many malignant lesions in multi- as in uninodular goitres. In any case statistics based on surgical material have not been substantiated by post mortem findings. Hazard and Kaufman (1952) found in 408 consecutive autopsies of adults in a so-called goitre area (Cleveland) 213 normal glands and 195 containing one or more nodules. In the goitrous glands they discovered one papillary adenoma, two papillary carcinomas and one non-encapsulated sclerozing tumour, none of which had produced clinical symptoms. It seems therefore that progress to malignancy is as rare an event in human nodular goitre as in that of the rat. When it happens it does not constitute necessarily a danger to life. Survival times of decades in untreated cases of papillary carcinoma are not exceptional even when the first symptoms were enlarged lymph nodes—sign of regional metastasis.

10. *Experimental Thyroid Tumours, an Example of Responsive Tumours.*

Whether the experimental thyroid tumours of the rat pass through a stage where they can regress completely is not known, but most of them remain dependent on hormonal stimulation during the lifetime of the host. Even when they have become cancerous and have spread to distant sites, they can still respond to variations in the blood level of TSH. Foulds (1951) distinguishes two types of tumours, responsive and non-responsive. Few pathologists or clinicians will offer serious objections to Fould's scheme of progression of tumours, the end-point of which is the unresponsive state. The writer is in complete agreement with the statement that " progression may be halted at any stage and does not always reach its end-point within the lifetime of the host". It should, however, not be forgotten that serial transplantation by means of which an experimental tumour might reach the final stage gives the latter the advantage of immortality. In the case of the thyroid tumours two hormones, TSH and TH decide the issue. Such a clearly discernible situation is unfortunately rare. There exist neoplasms where the problem is far more complicated and where two questions have to be asked : are they responsive ? and : to what do they respond ? The literature of the last years contains examples of cancers of the breast reacting first favourably to ovariectomy. Then they recur to regress perhaps under the influence of testosterone. They resume growth after a while to be arrested by adrenalectomy or hypophysectomy. Before attempts were made to control carcinomas of the breast by these means one would have considered all widely metastasizing cancers to have reached the end-point. That may have been true for many, but certainly not for all. These remarks do not imply any criticism of Foulds' scheme and of the concept of progression as an intrinsic property of the tumour cell. They are intended to point out how difficult it can be to be sure that the end-point has been reached.

In a recent review Furth (1953) referring to the experimental tumours of thyroid and pituitary writes : " Most current theories regard the basic alterations in cancer as residing not in the host but in the neoplastic cell ", and continues " in this review tumours will be surveyed which find their origin in alteration of the host ". Cancers of known etiology like, for instance, those induced by a chemical agent, such as benzpyrene, are growth-responses of a susceptible tissue to a chemical carcinogen. In the writer's opinion such neoplasms do not differ fundamentally from those resulting from continued stimulation by the secretions of an endocrine gland. What differs is the origin of the agents and perhaps their potency. Whether one studies the histogenesis of chemically-induced skin tumours or of experimental thyroid tumours, one sees the same sequence of events : first diffuse hyperplasia, followed by nodular hyperplasia and finally by frankly neoplastic growth. This in itself is highly suggestive for the similarity of the process induced by entirely different agents in two different organs.

As far as the granulosa and lutein cell tumours of the ovary of rodents are concerned, they can be obtained by various means : by gonadotrophic stimulation alone (Biskind and Biskind, 1944 ; Lipschutz, Ponce de Leon, Woywood and Gay, 1946), by the combination of an exogenous carcinogenic agent and hormonal stimulation (Bielschowsky and Hall, 1951) and, possibly, by a carcinogen alone (Marchant, Orr and Woodhouse, 1954). Even should it be proved that the granulosa cell tumours induced by 9 : 10 dimethyl-1 : 2-benzanthracene need for their development an elevated gonadotrophin level, there would only be a quantitative difference between them and, for instance, a methylcholanthrene-induced sarcoma. In the latter case normal amounts of pituitary secretions allow a neoplastic growth response to the carcinogen, but these sarcomas do not develop in the hypophysectomized rat (Moon *et al.*, 1952). Thus for the development of some chemically-induced neoplasms normal amounts of pituitary secretions are sufficient, some need more, and for tumourigenesis in the skin they do not appear to be necessary at all. On the other hand a fair number of neoplasms can be obtained by hormonal stimulation alone. It seems arbitrary to divide neoplasms into two categories—those induced by exogenous and other sinduced by endogenous agents. It has been argued that the experimental thyroid, pituitary and ovarian tumours differ from chemically-induced cancers because they depend for so long on continued stimulation. However, the same is the case with many neoplasms due to exogenous agents, such as the tar tumours of the rabbit's ear and the neurofibromas which appear in rats after feeding of ergot-containing diets (Nelson, Fitzhugh, Morris and Calvery, 1942). I see, therefore, no fundamental difference between a neoplastic growth response to a hormone and one to an exogenous carcinogen.

It has been pointed out before that certain thyroid tumours which were anaplastic and non-functional can become functionally active under the influence of thyrotrophic hormone. A similar observation was reported by Hooker, Pfeiffer and Strong (1947); Hooker (1948). A malignant non-functional interstitial cell tumour of a mouse changed under the influence of equine gonadotrophin to an androgene-secreting neoplasm, and although the tumour grew rapidly the primitive Leydig cells assumed the appearance of mature Leydig cells. Thus in responsive tumours growth and differentiation need not oppose each other.

### IV. The Retrogression of Tumours.

On February 27, 1900, Pearce Gould, of the Middlesex Hospital, London, demonstrated patients suffering from cancer of the breast showing signs of focal or systemic retrogression. He wanted to call attention to one point: " the fact of repair in cancer ". " A recognition of this fact will not affect our views of the true nature of cancer, but it will act as a constant stimulus to us to find out some method of treatment, some therapeutic agent, for this disease which will lead to this repair."

Some aspects of retrogression of tumours with special emphasis on the morphology of hormone-induced regression will be discussed in the last part of this contribution.

### 1. *Spontaneous Regressions.*

It is not my intention to review all recently recorded cases of spontaneous retrogression of cancer in man. Three well documented examples ought to suffice to convince the sceptic of the reality of the phenomenon.

Dunphy (1950) recorded a case of a post-menopausal woman, 54 years of age, with a tumour in the lower abdomen. Laparotomy revealed the presence of multiple tumour nodules in the omentum and of a mass which had involved uterus and adnexae and was adherent to loops of small intestine. The tumour was considered inoperable and the abdomen was closed after a biopsy had been taken. The histological diagnosis was small-cell carcinoma. After two years the patient had improved. Eight and a half years after the operation a mass appeared in the right groin which grew slowly for one year when the patient re-entered the hospital. The original tumour in the pelvis had disappeared and the uterus seemed to be normal; roentgenograms of the lung were highly suggestive for miliary metastases. The mass in the groin, encapsulated lymph nodes, were surgically removed and found to be full of malignant growth of a structure similar to that of the biopsy. Two years later—in the interval the patient had become a diabetic—a small lump was present on the right side of the neck which had developed during the preceding twelve months. This was found to be a benign lipoma. Thus thirteen years after an inoperable carcinoma had been found and four years after removal of metastatic lymph nodes the patient was alive and without signs of malignant disease. Sumner (1953) described a case of spontaneous regression of a malignant melanoma in a young woman. Three and a half years before the patient sought medical advice a black mole above the internal malleolus of the left leg had become infected and disappeared. During a following pregnancy a lump appeared in the left groin which was not treated. Three years later, when the patient was again pregnant for six months, nodules were present in the right breast, in the left arm near the shoulder, and several smaller ones in the subcutaneous tissue of the abdominal wall and the back. On operation the tumours of the right breast, left arm and left femoral region were found to be partly solid, partly cystic, deeply pigmented structures. The histological diagnosis was malignant metastasizing melanoma. Although the prognosis appeared hopeless, an attempt was made to remove the subcutaneous nodules also. Astonishingly enough, all the incisions healed although the tumours were torn during the operation. Two months later the patient delivered a normal child. Eight months post-partum she returned with a recurrence in the femoral region which was excised, as was another found in the right supraclavicular region seven months later. From then on no further metastases appeared and the patient seemed free of neoplastic disease four years after the first operation. Apparently this not very malignant melanoma of the left leg had produced metastases to the groin during the first pregnancy; then the process became stationary until, during the next pregnancy, multiple secondaries developed. Since the surgeon disclaims complete removal of all malignant tissue the melanoma which progressed twice during pregnancy must have undergone systemic retrogression. The third example is a case of sarcoma in a male baby (Penner, 1953). The tumour, discovered when the child was two months old, measured 5 cm. and had produced a defect in the left femur. The histological diagnosis of a biopsy specimen, confirmed by F. W. Stewart and F. W. Foote, was sarcoma. No treatment was given. When the child was traced five years later there was no evidence of neoplastic disease and the mother stated that at the age of nine months the tumour had disappeared. Of interest is that at the time the sarcoma of the leg was discovered a swelling was also present in the left sterno-mastoid muscle. When the child was re-examined at the age of five and a half years this area was atrophic.

These observations illustrate that retrogression of cancers with an extremely bad prognosis can occur at any time of life. There is no hint as to the factors responsible in two of them. Only in the case of the melanoma the clinical course suggests a hormonal influence. Whereas such cases are extremely rare departures from the usual course of malignant disease, there exist uncommon neoplastic lesions, such as papillomatosis of the larynx in children in which retrogression is the rule. Although generally of a self-limited character, regressing at the time of puberty, the condition has a high rate of recurrence and is potentially malignant. Walsh and Beamer (1950) have described two children in which the disease progressed to epidermoid carcinoma, and mention a third case. In contrast to the papillomas of the adult which are more often than not single, in children they are multiple and can spread into the trachea. Why, as reported by Cunning (1950), the papillomatosis of the upper respiratory tract in children has become increasingly rare is unknown. Attempts to bring on puberty by injection of testosterone failed to affect the papillomatosis in one case, but Broyles (1940) reports five remarkable regressions in children of both sexes under local treatment with oestrogen. Zalin (1948) had two successes with this therapy, whereas Gorrell (1952) was unable to influence the growth. That endocrine factors can influence the course of papillomatosis is clearly demonstrated by a case recorded by Holinger, Johnston and Anison (1950). The patient, a young woman, had undergone sixty-three endoscopic procedures for removal of recurrent laryngeal papillomas in thirteen years. Over this period she had three pregnancies and during each the papillomas disappeared to recur with the onset of menstruation. Whatever the etiology of the condition, there can be no doubt that hormonal factors have some influence on the regression or progression of papillomatosis of the larynx. In a way this condition shows an opposite trend to that of juvenile melanoma—it has greater growth potentialities prior to puberty. Facts like these should make one beware of generalizations. The question whether tumours are more malignant in the young than in the old cannot be answered, not even when cancers of the same organ are considered. For instance, inflammatory cancer of the breast is an extremely rare condition in women beyond the age of sixty, but when it occurs in the male patient it is found in the seventh or eight decade of life (Treves, 1953)..

### 2. *Morphology of Regression.*

Retrogressions induced by hormonal treatment have become an accepted fact (Nathanson, 1951, 1952). Studying the literature dealing with this phenomenon one is impressed by the paucity of adequate descriptions of the morphological changes occurring in tumours regressing under the influence of hormones. An attempt has been made to collect the existent data in the hope that others might feel inclined to fill the obvious gaps. The writer feels that in this way worthwhile information could be gained which might help in our understanding of the action of hormones on malignant growth. Based on nearly a hundred years of practical experience pathologists have learned to recognize the morphological signs which are the mark of cancer. When a tumour has invaded neighbouring tissue or has broken into blood-vessels and spread to distant parts a neoplasm is considered malignant. In the overwhelming majority of cases tumours having these characteristics kill the bearer earlier or later. Thus the conceptions of clinical and pathological malignancy are not at variance except for the relatively few types of neoplasms, which are less dangerous than the unexperienced would assume from their morphology.

(a) *Conditioned Growths.*

One of the best examples of experimentally-induced tumours, which look malignant under the microscope but biologically are not, are the tar-induced skin tumours of the rabbit. Studying these neoplasms Rous and Kidd (1939) encountered a great variety of growths widely different in their morphology but uniform in their behaviour. Some had the microscopical appearance of what they were, namely benign neoplasms, but others had become anaplastic, had invaded the deeper layers of the skin and sometimes were found inside lymphatics. Yet all, except the frillhorns, regressed once tarring was left off. The papillomas faded away and only the largest persisted because of their core, the covering epithelium reverting to an apparently normal epidermis. The so-called carcinoids, i.e. the neoplasms which looked like carcinoma, underwent differentiation to papillomas after connective tissue had walled them off. Massive necrosis of a carcinoid was seen only once. The same authors (1941) described two different processes in regressing tumours, one characterized by proliferation of the connective tissue coupled with inflammatory changes and another by atrophy of the tumour epithelium with cell loss exceeding replacement. Mackenzie and Rous (1941) have offered overwhelming evidence for the persistence of neoplastic cells in apparently normal skin after what appeared to be complete regression, another extreme situation showing the limitations of morphology. Two possibilities exist : inability of the observer's eye to pick out a few abnormal cells among the mass of normal elements or, as suggested by Rous and Kidd (1941), the neoplastic epithelium resumes the appearance of the normal. The regression of these tar tumours is due to the withdrawal of the stimulus which induced them and in the absence of which they cannot maintain themselves. Therefore it is doubtful whether the connective-tissue reaction found around regressing carcinoids is different from processes of repair seen when non-neoplastic tissue has been destroyed by a vascular accident or by necrosis. Not every conditioned growth undergoing regression provokes this reaction. In the rat prolonged administration of oestrogens leads to a tumourous enlargement of the pituitary which regresses as soon as the hormonal treatment ceases (Nelson 1944). First the cells forming these " adenomas " become smaller, the large Golgi apparatus disappears and their nuclei resemble those of the " wheel " cells of the ovary of the hypophysectomized rat. Then the centre undergoes liquefaction and finally few signs of their former existence remain.

In human pathology where the etiology of a neoplasm is rarely known, one cannot be sure whether regression of a tumour might not be due to the withdrawal of the inductive stimulus or to the elimination of a promoting factor essential for the growth of the neoplasm. Were it not for the now many times observed regression of carcinomas after administration of hormones one would have little evidence that the body disposes of agents capable of opposing malignant growth.

(b) *Malignant Growths.*

Before reviewing the literature dealing with the morphology of human cancers regressing under the influence of hormonal treatment it might be useful to dwell shortly on some observations made on spontaneously regressing cancers of man and on the findings of Foulds (1952) obtained in breast tumours of mice.

Since Virchow's days pathologists have been aware of degenerative processes in tumours and of the fibrous tissue reactions which accompany or follow these phenomena. Handley (1909) believed that regressive changes in tumours start always centrally and affect the oldest lesions first, and he described in detail the obliteration of invaded lymphatics by fibrous tissue. From the older literature only two other papers will be quoted. Erdheim (1930) described the repair of metastatic lesions in bone in a case of metastasizing ovarian cancer which had spread into many parts of the skeleton. Already at naked-eye inspection these lesions differed from the ordinary. From a detailed study of many metastatic foci in the bones Erdheim drew the following picture : retrogression of cancellous tissue starts in foci, sometimes in the centre, occasionally in the periphery until the greater part of the mass is involved. He distinguished two types, atrophy and necrosis. In the case of the former the number of tumour units diminish, their cells become pale and small, so that the stroma predominates and finally is the only remaining element. In the latter case all elements, including cancellous trabeculae situated in the metastasis, become necrotic. Mesenchymal elements derived from the stroma of the tumour, i.e. originally from the reticulum of the bone marrow, penetrate into the necrotic area while phagocytes remove the debris. The whole area of necrosis is then replaced by scar tissue. Its fibres are delicate and loosely knit, never assuming a scirrhus character. Sometimes cancer cells situated in the tissue surrounding the bone try to re-invade it from outside. When this happens the connective tissue which has replaced a necrotic metastasis appears immune against this invasion. According to Erdheim (1930) the malignant cells advancing against the scar grow like a benign adenoma, compressing the scar tissue but never infiltrating it. Erdheim laid stress on the particular quality of this connective tissue and of the phagocytes contained in it. He expressed the belief that such scar tissue possesses a local immunity against malignant growth. This paper has been quoted *in extenso* because it is one of the few accounts known to the writer in which morphological evidence is offered suggestive of a qualitative difference between ordinary connective tissue and the stroma of tumours.

In Sampson's (1931) paper on the reaction of the peritoneum to the implantation of cancer cells of ovarian origin similar opinions are expressed. He compares the reaction of the peritoneum to implanted tumour cells with that to an inert foreign body. The cancer cells are enmeshed in fibrin which becomes organized by ingrowing fibroblasts with or without the aid of vascular endothelium. The granulation tissue can then be transformed into connective tissue encapsulating the neoplastic cells. From the following quotation it will be seen that Sampson, like Erdheim, believed in an acquired local immunity to malignant growth. " Does the peritoneum of patients with peritoneal carcinomatosis actually develop a relative immunity to the implantation of cancer on its surface ? I believe that this occurs in some instances."

The hypothesis of local or constitutional immunity, still expressed in the 1940 edition of Ewing's famous work, figures rarely in modern writing, but the conception of the important role of the stroma in tumour regression is very much alive, as will be seen later.

Lastly, some unusual morphological findings from Sumner's (1953) aforementioned case of regressing melanoma deserve a short description. Of special interest are the changes in one of the lymph nodes removed from the groin. Here lymphatic tissue was only present in the periphery of the node. The centre consisted of dense hyaline fibrous tissue with an area of calcification. In one region the connective tissue was more loosely arranged and contained malignant melanoma cells in different stages of degeneration together with plasma cells, lymphocytes and polymorphs in moderate numbers. The blood vessels had greatly thickened fibrotic walls and a narrow lumen. On the basis of these findings the pathologist assumed that the patient had received radiation treatment ; however, this was not the case.

The histological changes occurring in mammary cancers of mice which retrogress after parturition were followed by Foulds (1952). At the height of their growth these neoplasms consist of radiating ducts filled with epithelial cells, many of them dividing. After parturition the neoplastic epithelium becomes desquamated and is apparently extruded by way of the ducts. At the same time the stroma becomes denser and finally a compact fibrous tissue, rich in collagen, compresses the duct-like structures. When these tumours resume their growth collagen disappears and a loose stroma surrounds the growing tubules. Although it is not known whether the regression is due to the withdrawal of a growth promoter or to an inhibitory agent active after parturition, it is interesting to note that in these mice the tumour epithelium as well as the stroma undergoes a sequence of changes which resemble those which can occur in cancers of the human breast.

### 3. *Morphology of Induced Regression.*

(a) *By oestrogens in mammary cancers.*

Few papers describe in detail the histological changes induced by oestrogens in mammary cancers. The first account was given by Koller (1944) in an addendum to the famous paper of Haddow, Watkinson and Paterson (1944). Koller's material came from a previously untreated spheroidal cell cancer of the breast of a woman of 64 years of age. She received 15 mg. of stilboestrol weekly by intramuscular route and in addition 1 mg. daily *per os*. The primary tumour as well as the axillary lymph nodes diminished in size and the ulcerated skin healed. Four biopsy specimens were taken in approximately monthly intervals, the first before treatment started and the others during the period of clinical regression. Originally the tumour contained 12·5–27·3 per cent of potentially dividing cells and the division rate was 2·3, 5·2, 5·6 and 7·5 per cent in the four areas studied. After one month the division rate did not surpass 4·6 per cent. In addition new cytological features appeared, vacuolation of nuclei, intensification in the stainability of the cytoplasm and abnormal mitoses. Degenerated tumour cells had increased from 2·5 to 12·5 per cent. The third specimen showed a very similar picture, but in the fourth a new rise in mitoses was found ; many of these, however, were frankly abnormal. The number of degenerated cells reached 16·3 per cent. Koller interpreted his findings as an indication for primary damage to the nucleus and for stilboestrol-induced breakdown in the mitotic mechanism which became apparent in the fourth biopsy specimen.

Huguenin, Saracino and Gerard-Marchand (1951) described the reaction of two mammary cancers to hexoestrol. One patient, a woman 73 years old, suffered from an enormous, ulcerating and fungating cancer which had apparently not spread to the axilla. The pre-treatment biopsy revealed a carcinoma of predominantly glandular character, the tumour cell acini being lined by cuboid or cylindrical cells. In other areas, however, solid epithelial masses were found spreading into the loose connective tissue. The patient received 20 mg. of hexoestrol for 9 days, 40 mg. for 53 and finally 60 mg. with excellent results. The next biopsy was performed after 2260 mg. of hexoestrol had been given. On the whole the picture resembled that of the first ; tumour cell acini surrounded by strands of fine fibres were still present, but the cancer cells showed definite degenerative changes. They were less well outlined, irregularities in chromatin content and shape of the nuclei more pronounced and occasionally the nuclei seemed to break up. These regressive changes were most advanced in the more isolated tumour units accompanied by intense round cell infiltration of the stroma. Four weeks later (3100 mg. hexoestrol) the tumour had become a scar with a small ulcer in the centre. A specimen taken from this area showed only granulation tissue with dilated capillaries and infiltrated by leukocytes, predominantly polymorphs. Next to the ulcer healthy epidermis covered a scar tissue rich in collagen and round cells. Six weeks later a fourth biopsy showed a further increase in the density of the collagen, perivascular agglomeration of round cells still persisted and occasionally minute calcium deposits were found in the section, but there was no evidence for the presence of tumour cells.

The second patient, a woman of 74 years, had a recurrence in the breast sixteen months after the primary growth had responded well to thermocautery and X-ray treatment. It was an adenocarcinoma differing from the one described above by growing in solid sheets, which were surrounded by a scanty connective tissue. The tumour was well differentiated in parts, but contained also more anaplastic areas with numerous mitoses and where invasion of blood vessels had taken place. This patient received 20 mg. of hexoestrol for 12 days, 40 mg. for 3 weeks and 60 mg. for 2 months. After two months of therapy the morphology of the carcinoma had changed into that of a quite atypical tumour. Where larger accumulations of cancer cells persisted remnants of pseudoacini were still recognizable ; but the cells were less well defined and their nuclei pyknotic or swollen. The stroma now formed the predominant part of the neoplasm. Instead of invasion of the connective tissue by the cancer cells mesenchymal cells seemed to advance in between the tumour epithelium, a picture resembling that of a foreign body reaction. Finally the dispersed tumour cells underwent lysis, sometimes whole groups becoming necrotic and occasionally impregnanted with calcium. A moderate inflammatory reaction accompanied these changes. When 60 mg. of hexoestrol had been given for two months only a small hard nodule remained in the area formerly occupied by the tumour and the lymph nodes were not any more palpable. A radical mastectomy was performed, but the wound healed so slowly that a new recurrence was suspected. A biopsy showed granulation tissue, but no tumour cells. It is of interest that a small squamous cell carcinoma of the face remained stationary at the time the breast cancer regressed. The French authors, although well aware of the regressive changes in nuclei and cytoplasm of the tumour cells, stress the importance of the stromal reaction. They believe that the epithelium-connective tissue relationship is shifted in favour of the latter and see in the sclerosis an active process and not a simple replacement fibrosis. Sirtori and Grattavola (1947) investigated the reaction of the connective tissue to oestrogen. From experimental findings and from their own observations in man they arrived at the following conclusions : Oestrogen stimulates the mesenchymal tissue in general, but this stimulation is more pronounced in the breast than, for instance, in the skin. As far as mammary cancers are concerned they state that the cytological changes observed in tumour cells under treatment with oestrogen do not run parallel with clinical findings and that they do not explain the softening of the tumour, which is clinically so often the outstanding feature of retrogression. They interpret the beneficient effect of eostrogen therapy as due to a modification of the stroma around the tumour cell nests. It consists in the substitution of a sclerotic dense stroma by one which is loose, oedematous, rich in fibres and contains many histiocytes. They believe that the different response of pre- and post-menopausal women to oestrogen can be explained by the state of the tumour stroma. In the younger age group the stroma is already loose and cellular, whereas in the older women it is sclerotic. Only the latter will change under the influence of hormonal therapy. In a second well-illustrated communication Sirtori (1951) gives additional data. He describes the striking difference in the reaction of the mammary gland tissue of a young man to oestrogen and to androgen. The former induces proliferation of the connective tissue, i.e. hyperplasia and the latter sclerosis, considered to be a regressive condition. Sirtori found exactly the same changes in the stroma of mammary cancers after treatment with these two hormones and he considers them striking enough to allow a diagnosis of the agent used. Thus degenerative changes which admittedly occur in mammary cancer cells under oestrogen treatment are, in Sirtori's opinion, less important than the activation of the stroma which takes place not only in the primary tumour but also in the metastases.

Emerson, Kennedy, Graham and Nathanson (1953) have given the most comprehensive account of the changes which occur at the time of clinical regression in mammary cancers of patients treated with oestrogens. The core of their paper are observations on total mastectomy specimens from thirteen primary metastasizing tumours. Twelve of the patients were post-menopausal women and one was a man 54 years of age. His mammary cancer had regressed after orchidectomy and he was treated with stilboestrol when, eighteen months after the operation, the tumour showed renewed signs of growth. Like in the cases recorded by Huguenin, Saracino and Gerard-Marchand (1951) this publication provides histological evidence that under oestrogen treatment some mammary cancers undergo retrogressions of such an extent that one can speak of near complete disappearance of the malignant cells from the primary focus as well as from the axillary glands. At the same time the paper contains examples of discrepancies between the cardinal clinical sign of regression—softening of the tumour—and the histological evidence indicating progression. Two main alterations in regressing tumours were observed—loosening of the stroma and degenerative changes in the cancer cells. The predominant change in the connective tissue occurring during the first month of treatment consisted in a reduction of the formerly dense collagen and its replacement by a loose tissue formed by narrow strands of collagen fibres and by fibrocytes with large nuclei. Emerson and his collaborators compare this loosening of the tumour stroma with the changes in the periductal connective tissue of young women during pregnancy or in the pre-menstruum, and point out that here, too, this process is limited to the connective tissue adjacent to epithelial elements. In earlier phases of retrogression degenerating cancer cells, together with plasma cells and lymphocytes, were still present. Later on these cells disappeared, new collagen as well as elastic fibres formed, so that in the end there remained nothing but a fibrotic area formed by a sparsely cellular dense connective tissue. The same end result as in the primary tumour could be observed in invaded lymph nodes. In the cases which responded best the lymph glands were transformed into scar tissue in which a few degenerated cancer cells remained. In regressing skin lesions the alterations in the connective tissue were less prominent, but occasionally lymphatics were found obliterated by fibrotic plugs. The degenerating tumour cells showed a swollen, pale or vacuolated cytoplasm, karyolysis or pyknosis of nuclei and fragmentation or shrinkage of cells. Comparison of the material obtained from patients with induced regression with that of 110 mastectomy specimens from breast cancer patients not previously treated revealed that the magnitude of the changes found in the former was of quite a different order. Foci of degenerating tumour cells and stromal changes of a similar character as described above were occasionally seen in the material from untreated patients, but the areas so affected were never large enough to suggest a general tendency for regression. Of interest is the observation that all but one of the regressing cancers showed a marked hyperplasia of the elastic tissue prior to treatment. This hyperplasia increased under the influence of oestrogens. Tumours of low to medium malignancy with a tendency to fibrosis and hyperplasia of elastic fibres were, in the experience of the American workers, most likely to show a favourable response to oestrogen. Which of the two major alterations, degeneration of tumour cells or connective tissue changes, occurs first they were unable to ascertain. There was no evidence that one preceded the other. They conclude that the main effect of the hormone is to strengthen a pre-existent reactive response of the connective tissue to the tumour and express the opinion " that these regressions were in part the result of an unusual hormonal stimulation of a natural occurring process of repair".

It should be taken into account that the findings just discussed come from a selected material, i.e. from cases showing unusual degrees of retrogression. This may explain why some authors were unable to discover consistent morphological changes in breast cancers which clinically improved under oestrogen treatment (Godwin and Escher, 1951 ; Dargent and Papillon, 1951).

A possible defensive role of the subepithelial connective tissue surrounding mammary ducts filled with cancer cells has been proposed by several authors, more recently by Bohle (1951). The findings of Huguenin, Sirtori and Emerson *et al.* give substance to this hypothesis and offer at least suggestive evidence that a defensive process against malignant growth can be set in motion by oestrogens in certain cases of cancer of the breast.

(b) *By oestrogens in prostatic cancers.*

Schenken, Burns and Kahle (1942) were the first to give a well illustrated account of the morphological changes occurring in prostatic cancers of patients treated for periods of 25–46 days with stilboestrol or its dipropionate ester. (The case histories are given in the paper of Kahle, Ogden and Getzoff, 1942.) The principal findings in these biopsies were marked shrinkage of nuclei to about half their former size and vacuolation of the cytoplasm in the tumour cells. Although slight cytoplasmic vacuolation is not an unusual feature of prostatic cancer cells, the degree observed after administration of synthetic oestrogens surpassed by a wide margin what could be found in untreated patients. The figures 14–17 of Schenken *et al.* (1942) paper give a clear picture of the different stages of cell degeneration, ranging from pyknosis of nuclei and ballooning of the cytoplasm to cell destruction, i.e. the formation of clear spaces having the shape of acini and containing only nuclear fragments. Groups of healthy-looking tumour cells remained among the degenerating elements. Heckel and Kretschmer (1942) observed similar degenerative changes in the prostatic cancer of a patient treated with stilboestrol for 233 days. Fergusson and Pagel (1945) tried to assess quantitatively the reduction in the number of tumour units as well as the coincident decrease in nuclear size of neoplastic cells in five carcinomas of the prostate which responded with retrogression to oestrogen therapy. Tumour cell counts in a second biopsy specimen taken 10–30 months after commencement of treatment showed that their numbers had declined to one-third to one-sixth of the pretreatment values. In three cases the nuclear diameter was significantly smaller. To judge from the photomicrographs the stroma increased in amount and density during the period of treatment. The paper describes and depicts also the disappearance of acid phosphatase from metastatic tumour cells after 24 days of administration of dienoestrol. Continuing these investigations Fergusson and Franks (1953) noted that already one week after the onset of treatment regressive changes were recognizable in the prostatic cancer cells, vacuolation of the cytoplasm being the first sign. The affected cells increased in size, assumed the shape of signet cells and their nuclei became denser or, occasionally, vacuolated. Desquamation and rupture of the neoplastic cells were the end result of this process. At the same time the stroma became imbued with products of disintegration and converted into a loose, fibrillar, ill-defined tissue which had an affinity for basic dyes. Replacement fibrosis was sometimes seen at a later stage. In two cancers squamous metaplasia was observed, once in the primary tumour and once in a metastic nodule situated in the liver—possibly a reaction of the tumour cells to oestrogen similar to that of the normal transitional epithelium of the prostatic urethra. Although each type of prostatic cancer cell—the authors distinguishing between a clear or reticular, a dark basophilic and an anaplastic type with vesicular nucleus—showed approximately equal sensitivity to oestrogen, the dark elements were found more frequently among the surviving cancer cells, present in all specimens.

From these observations it appears that in the case of prostatic carcinoma the reaction to oestrogen is far less complex than in mammary cancers and, what facilitates the interpretation, the first changes appear after a remarkably short interval. Fergusson and Franks (1953) refer to observations of their own in which the neoplastic cells were already affected by oestrogen after less than 24 hours, a period shorter than that needed for the induction of oestrus in a gonadectomized rodent. These findings are, in the writer's opinion, highly suggestive for a direct effect of the hormone on the neoplastic cell.

Quite a different type of regression was seen by Franks (1953) in a case of prostatic cancer. Since treatment with large doses of stilboestrol had failed to influence pain and dysuria in the 63-years-old diabetic patient, adrenalectomy was performed. No immediate clinical improvement followed the operation and stilboestrol therapy was reinstituted. Death occurred on the 40th post-operative day in consequence of an infarction in the lung. The post-mortem examination revealed the presence of a large carcinoma of the prostate which had invaded the seminal vesicles and the bladder. Metastases were found in liver, lungs and in many abdominal and mediastinal lymph nodes. Histologically the primary tumour as well as all the secondaries showed massive central necrosis, surrounded by a rim of apparently viable tumour. The two zones were separated by granulation tissue. The central necrosis involved tumour cells as well as stroma, and even some of the larger blood vessels There was a gradation in the intensity of the degenerative changes, which reached the maximum in the centre. From the study of small metastatic foci in which the blood vessels were not affected Franks concluded that the primary effect had been on the tumour cells. This case provides good morphological evidence for acute systemic regression in a primary cancer and its metastases without active participation of the stroma.

The factors responsible for the destruction of this widely metastasizing neoplasm cannot be easily assessed. However, it seems possible that the large doses of stilboestrol given were of greater importance than the adrenalectomy. Some support for this contention comes from Baker's (1953) observations on two patients, the metastatic pain of which was not ameliorated by oestrogen prior to but after adrenalectomy. Whereas an increased and sometimes even fatal androgen effect after adrenalectomy is a well-established fact, no corresponding data exist so far in the case of oestrogens.

In prostatic smears from patients treated with oestrogens Peters (1950) found regularly large highly vacuolated cells, the apparently empty cytoplasm of which was in fact filled with glycogen. These epithelial elements disappeared rapidly from the smear when hormone therapy was withheld. According to Peters these glycogen-rich cells are non-neoplastic and easily distinguishable from carcinoma cells, which are shrunken, have poorly stainable nuclei and can disappear under oestrogen treatment (Peters and Frank, 1952). Papanicolaou (1949), however, mentions that not only normal but also prostatic cancer cells may show cellular and nuclear enlargement under these conditions. I have been unable to find any reference to the glycogen content of the vacuolated neoplastic cells characteristic for an oestrogen senstive prostatic carcinoma. The pictures of Peters (1950) resemble so much the balloon cells of Schenken *et al.* (1942) and of Fergusson and Franks (1953) that it seems worthwhile to investigate whether the neoplastic prostatic epithelium accumulates glycogen under the influence of oestrogen.

(c) *By progesterone in cancer of the cervix.*

Hertz, Cromer, Young and Westfall (1951) discovered that some carcinomas of the cervix of pre- and post-menopausal women regressed under progesterone treatment. Platt (1952) gave a description of the morphological changes noted in cervical smears and in biopsy specimens obtained from these patients. In cases responding favourably a change in the composition and cytology of the smear was already recognizable at the third day of progesterone administration. Blood and cellular debris diminished and normal squamous epithelial cells became more numerous. The cancer cells increased in size, their nuclei losing the compact and assuming a more granular appearance. The same trend was also seen in sections of the tumours. In a few patients cancer cells disappeared completely from the smear and post-treatment biopsies failed to reveal the presence of epidermoid carcinoma. A comparison of pre- and post-treatment tissue sections stained with Best's carmine disclosed differences in " glycogen " content of normal and neoplastic squamous epithelium. In post-treatment specimens less glycogen was found in the nuclei and hardly any in the cytoplasm ; but an accumulation of intercellular carmine-positive material was noted together with increased amounts of fluid. Platt mentions that similar effects of progesterone were seen in one case of squamous cell carcinoma of the vulva. It seems desirable to check the shift of the Best carmine-positive material with more specific staining methods.

(d) *By other means.*

Literature on the morphology of testoterone-induced regressions of mammary cancers is practically non-existent. Adair and Hermann (1946) quote in their paper the reports of Fred Stewart on the biopsies of two of their cases which regressed under testosterone treatment. The material came from metastatic skin nodules. " Rather marked focal hydropic degeneration, mitoses still present " and " cells definitely hydropic and nuclei pyknotic. Rare mitoses still seen. The tumour shows distinct differences from the expected." I do not know of any comprehensive study which confirms or refutes Sirtori's (1951) findings of the sclerotizing action of androgens on the stroma of mammary cancers. Apparently testosterone affects more frequently metastases to the bone, and many excellent X-ray photographs testify to the far-reaching regressions they can undergo under androgen treatment. However, lesions which on radiological evidence appear completely healed can contain still considerable amounts of viable tumour, as revealed by post-mortem studies (Preston, Taylor and Crumrine, 1949).

Although excellent descriptions of clinical regressions of mammary cancer in man after orchidectomy are available (Treves, Abels, Woodard, and Farrow, 1944 ; Treves 1949) the corresponding morphological changes have not yet been fully described, and the same scarcity of data exists for tumours regressing after adrenalectomy and hypophysectomy.

Emerson *et al.* (1953) mention the alterations in an adenocarcinoma of the breast in a middle-aged man after orchidectomy. Three biopsies taken during the period of post-operative regression revealed degenerative changes of quite considerable extent, but already, six months after orchidectomy, foci of less differentiated carcinoma were discovered in a lymph node.

Luft and Olivecrona (1953) depict the changes which occurred in a typical gelatinous mammary cancer following hypophysectomy. Before the operation the amount of colloid was moderate and epithelial elements predominated. Four months after the operation few groups of malignant cells remained and the mucoid material was abundant. Five months later only isolated cancer cells were present, there was less mucoid material and the connective tissue had increased. Histological examination of several organs of a hypophysectomized patient who had died of malignant melanoma (Shimkin, Boldrey, Kelly, Bierman, Ortega and Naffziger, 1952) revealed some unusual features in metastatic nodules situated in liver and spleen. The two " secondaries " in the liver were found to consist of a dense collagenous scar tissue containing pigment granules, but no tumour cells. In the spleen the metastases had a hyalinized or necrotic centre and even in the periphery there was a considerable amount of fibrous tissue. Here the tumour cells appeared degenerated, some had hydropic nuclei, others were remarkably small and appeared shrunken. On the other hand, no morphological evidence for regression was found in the metastases in jejunum, lymph glands and lungs.

4. *Changes in Endocrine Organs of Patients Suffering from Malignant Disease.*

Surgical removal of the gonads, the adrenals and the pituitary has been performed with the intention of arresting the progress and spread of cancers which could no longer be controlled by other means. Since the earliest days of this century, when British surgeons were well aware of the benefit of ovariectomy in some cases of mammary cancer, progress has been slow in the understanding why the response to the operation varied so greatly. Already in 1900 Boyd suggested " that certain ovaries, probably by pathological variation in their internal secretion, favour the growth of cancer by action either upon the growth or upon the tissues ; the removal of such ovaries alone will be of benefit ". Progress has come from the study of the ovaries and secondary sex organs of post-menopausal women. The old conception of a functionally inactive gland has given way to the recognition that the ovary can secrete oestrogens for considerable periods after menstruation has ceased. Examination of vaginal smears showed that in many post-menopausal women the mucosa is far from being atrophic and the same applies to the endometrium. In 1941 Smith called the attention to abnormalities in the post-menopausal ovary of women with endometrial cancer. Instead of a narrow cortex rich in collagen he found a wide cellular outer zone. These changes occurred more often in cases of cancer of the corpus uteri than in normal women. A later study by Woll, Hertig, Smith and Johnson (1948) confirmed and enlarged Smith's previous findings. The main features of the condition they described and named thecomatosis were nodular masses of plump cells, which had an enlarged nucleus and prominent nucleolus and dipped deep into the stroma. Sometimes areas of epithelial-like lipoid-containing cells were also present and were considered to be luteinized theca cells. The publications of Laffargue, Luscan and Lavernhe (1952) and of Dockerty, Lovelady and Foust (1951) contain confirmatory findings. Very similar changes in combination with medullary vascular hyperplasia were found by Sommers and Teloh (1952) in 83 per cent of patients which had died of carcinoma of the breast and in 37·6 per cent of controls. A high degree of hyperplasia occurred only once in the non-cancer group. McManus and Sommers (1952) found that women with ovaries showing thecomatosis at the time they were surgically removed had a longer survival time than patients with atrophic ovaries. In the latter group of breast cancer patients the post-castration survival was so short that the authors consider the possibility whether ovariectomy had not produced actual harm. Burt and Castleman (1953) also observed a high incidence of ovarian stromal hyperplasia in women with carcinoma of the breast.

The findings of Sommers and his associates might provide an explanation for some extraordinary post-castration regressions. Raven (1950) reported a remarkable case of metastasizing spheroidal cell carcinoma of the breast which retrogressed after ovarietomy to such an extent that two years after the operation no signs of malignant disease were detectable. In one of the ovaries of this patient the pathologist found a wide, ill-defined zone of theca cell formation.

As far as the testes are concerned they do not show specific changes in either cancer of the breast or of the prostate. In the adrenals of some of their patients with advanced mammary cancers Huggins and Dao (1953) found evidence for hyperplasia especially marked in the zona glomerulosa. Burt and Castleman (1953), too, noted that the average adrenal weight in women with cancer of the breast exceeds that of adrenals from patients without carcinoma. These authors gave the only modern description of the cytology of the adenohypophysis in malignant disease of the breast. They found an increase in the basophils and in the hypertrophic amphophils, an abnormality not specific for mammary cancer.

This important paper contains interesting observations on the effect of oestrogens on the human pituitary.

Of the morphological investigations reviewed above only those on the ovaries have contributed so far to our understanding of malignant disease. There seems to be a definite need for more information, not only on the pathology of the ovaries but specially of the pituitary, adrenals and testes.

### 5. *Discussion.*

The findings presented in the section on hormone-induced regression will, it is hoped, be accepted as evidence that physiological agents can influence profoundly some malignant growths. It could be argued that the therapeutic results obtained are due to the administration of pharmacological doses and therefore should not be used as evidence for a naturally occurring hormonal defence mechanism. Actually the amounts of oestrogen needed for arrest of a susceptible carcinoma of the prostate are rather small. Using the level of serum aldolase, or of acid phosphatase as indicator, Baker and Govan (1953) and Baker *et al.* (1953) found doses of 0·25–0·5 mg. effective. Similar amounts control the symptoms of the menopause without inducing hyperplasia of the endometrium. Thus they do not fall outside the physiological range. Admittedly sometimes much higher doses are needed to influence malignant growth. In systematic studies over twenty years Lipschutz (1950) and his pupils have firmly established the quantitative relationship between tumour growth-promoting steroids and those opposing it. Lipschutz (1952, 1954) has drawn attention to the danger which may result when rhythmic processes are converted into continuous ones and sees in the rhythmic release of ovarian hormone one of the means of "antitumoural auto-defence". As far as the amounts of steroid needed for tumourigenesis are concerned he found them to be much smaller than was believed previously; in fact, physiological amounts, acting unopposed, could induce neoplastic growth. With increasing knowledge of the natural history of tumours responding to hormonal action the gap between clinical and the experimental findings of the Chilean workers has narrowed.

Stewart (1952) has recently recorded his experiences in spontaneous regression of neoplasms. He considers the disappearance of *in situ* epidermoid carcinoma of the uterine cervix a distinct possibility, an opinion shared by other students of this condition (Reagan, 1952; Hoffman, Farell and Hahn, 1953). After the demonstration of the beneficial effect of progesterone on such or even more advanced lesions (Platt, 1952) one could envisage a shift of a disturbed steroid hormone balance towards predominance of the corpus luteum hormone as a possible mechanism for retrogression of these neoplasms. Slight variations in hormone level might also account for the appearance of late metastases of hormone dependent tumours.

### 6. *Regressions Induced by Immunological Reactions.*

Stewart's (1952) paper contains a unique observation. A patient suffering from myosarcoma of the uterus reacted to irradiation with what resembled an anaphylactic reaction and the inoperable tumour disappeared in the course of a few days. Five years later she was again treated with radium for a lesion of the cervix. Again high fever, an urticarial rash and eosinophilia appeared, proof that the first reaction and the regression of the tumour were not due to specific sensitization against the cancer. There is little evidence that antibodies elicited by a neoplasm have ever produced enough tumour-specific antigens to induce its destruction (Hauschka, 1952). However, immunogenetical reactions may play a role in the regression of choriocarcinomas. This malignant growth of foetal origin could provoke an antigenic response when invading maternal tissue. Two facts support this hypothesis. Park and Lees (1950) on the basis of an analysis of 516 cases of choriocarcinoma conclude : " True regressions occur in a small but certainly significant proportion of cases ". Late recurrences, if they occur at all, are extremely rare as compared with carcinoma of the breast. The authors state : " Choriocarcinoma kills either within 12 months of diagnosis or not at all", a most unusual behaviour for a malignant growth. The high death-rate from choriocarcinoma recorded in the older literature was probably wrongly ascribed to the malignant properties of the tumour itself. The mortality from choriocarcinoma seems to have declined in the last decades since blood transfusion and chemotherapy have lowered the risk of death from haemorrhages and puerperal sepsis. When these complications were eliminated astonishingly high survival rates resulted. They cannot be dismissed with the assumption that the condition was overdiagnosed. For instance in a series of 22 cases described by Huber and Hörmann (1952) there were 19 survivors, in 6 of which metastases had been present.

These observations may be taken as an indication that apart from hormones other, not yet identified, bodily agents may be able to induce regression in malignant growths.

### REFERENCES.

Adair, F. E. and Hermann, J. B.—(1946) *Ann. Surg.*, **123**, 1023.
Armstrong, E. C. and Bonser, G. M.—(1947) *J. Path. Bact.*, **59**, 19.
Axelrad, A. and Leblond, C. P.—(1953) *Proc. Amer. Ass. Cancer Res.*, **1**, 2.
Baker, R. and Govan, D.—(1953) *Cancer Res.*, **13**, 141.
*Iidem*, Huffer, J. and Cason, J.—(1953) *J. clin. Endocrin.*, **13**, 383.
Baker, W. J.—(1953) *J. Urol.*, **70**, 275.
Ball, H. A. and Samuels, L. T.—(1936) *Amer. J. Cancer*, **26**, 547.
Bielschowsky, F.—(1944) *Brit. J. exp. Path.*, **25**, 90.—(1945) *Ibid.*, **26**, 270.—(1949) *Brit. J. Cancer*, **3**, 547.—(1953) *Ibid.*, **7**, 203.
*Idem*, Greisbach, W. E., Hall, W. H., Kennedy, T. H. and Purves, H. D.—(1949) *Ibid.*, **3**, 541.
*Idem* and Hall, W. H.—(1951) *Ibid.*, **5**, 331.
*Iidem*—(1953) *Ibid.*, **7**, 358.
Biskind, M. S. and Biskind, G. S.—(1944) *Proc. Soc. exp. Biol. N.Y.*, **55**, 176.
Bohle, H.—(1951) *Frankfurt. Z. Path.*, **62**, 167.
Bondy, P. K.—(1951) *Proc. Soc. exp. Biol. N.Y.*, **77**, 638.
Boyd, S.—(1900) *Brit. med. J.*, **2**, 1161.
Brachetto-Brian, D. and Grinberg, R.—(1951) *Rev. Soc. argent. Biol.*, **27**, 199.
Broyles, E. N.—(1940) *Johns Hopk. Hosp. Bull.*, **66**, 319.
Burt, A. S. and Castleman, B.—(1953) *Cancer*, **6**, 236.
Clausen, H. J.—(1953) *Proc. Soc. exp. Biol. N.Y.*, **83**, 835.
Cole, W. H., Slaughter, D. F. and Rossiter, L. J.—(1945) *J. Amer. med. Ass.*, **127**, 883.
Cope, O.—(1952) *New Engl. J. Med.*, **246**, 368, 408, 451.
Cordier, R., Craps, L. and Martin, Ph.—(1951) *Ann. Endocr., Paris*, **12**, 244.

Cox, A. J., Jr., Wilson, R. H. and DeEds, F.—(1947) *Cancer Res.*, **7**, 647.
Crile, G., Jr.—(1953) *New Engl. J. Med.*, **249**, 585.
Cunning, D. S.—(1950) *J. Amer. med. Ass.*, **142**, 73.
Dalton, A. J., Morris, H. P. and Dubnik, C. S.—(1945) *J. nat. Cancer Inst.*, **5**, 451.—(1948) *Ibid.*, **9**, 201.
*Idem*, Morris, H. P., Striebich, M. J. and Dubnik, C. S.—(1950) *Ibid.*, **11**, 391.
Dargent, M. and Papillon, J.—(1951) *Bull. Ass. franç. Cancer*, **38**, 352.
Dobyns, B. M. and Lennon, B.—(1948) *J. clin. Endocrin.*, **8**, 732.
Dockerty, M. B., Lovelady, S. B. and Foust, G. T., Jr.—(1951) *Amer. J. Obstet. Gynec.*, **61**, 966.
Doniach, I.—(1950) *Brit. J. Cancer*, **4**, 223.—(1953) *Ibid.*, **7**, 181.
Duffy, B. J., Jr. and Fitzgerald, P. J.—(1950) *Cancer*, **3**, 1018.
Dunphy, J. E.—(1950) *New Engl. J. Med.*, **242**, 167.
Emerson, W. J., Kennedy, B. J., Graham, J. N. and Nathanson, I. T.—(1953) *Cancer*, **6**, 641.
Engelbreth-Holm, J. and Jensen, E.—(1953) *Acta path. microbiol. scand.*, **33**, 257.
Erdheim, J.—(1930) *Virchows Arch.*, **275**, 383.
Ewing, J.—(1940) 'Neoplastic Diseases.' Philadelphia and London (W. B. Saunders Co.).
Fergusson, J. D. and Franks, L. M.—(1953) *Brit. J. Surg.*, **40**, 422.
*Idem* and Pagel, W.—(1945) *Ibid.*, **33**, 122.
Fischer, O.—(1926) *Virchows Arch.*, **259**, 9.
Fitzgerald, P. J. and Foote, F. W. Jr.—(1949) *J. clin. Endocrin.*, **9**, 1153.
Foulds, L.—(1951) *Ann. R. Coll. Surg. Engl.*, **9**, 93.
Foulds, L. F.—(1952) *Ciba Foundation Colloquia on Endocrinology*, **1**, 124.
Franks, L. M.—(1953) *Brit. med. J.*, **2**, 359.
Fraser, A. S. and Nay, T.—(1953) *Aust. J. biol. Sci.*, **6**, 645.
Furth, J.—(1953) *Cancer Res.*, **13**, 477.
*Idem* and Burnett, W. T., Jr.—(1951) *Proc. Soc. exp. Biol. N.Y.*, 78, 222.
*Idem*, Burnett, W. T., Jr. and Gadsden, E. L.—(1953) *Cancer Res.*, **13**, 298.
*Idem*, Gadsden, E. L. and Burnett, W. T., Jr.—(1952) *Proc. Soc. exp. Biol. N.Y.*, **80**, 4.
*Idem*, Gadsden, E. L. and Upton, A. C.—(1953) *Ibid.*, **84**, 253.
Gabe, M. and Arvy, L.—(1947) *Experientia*, **3**, 193.
Godwin, J. T. and Escher, G. C.—(1951) *Cancer*, **4**, 136.
Goldberg, R. C. and Chaikoff, I. L.—(1950) *Endocrinology*, **46**, 91.—(1951*a*) *Proc. Soc. exp. Biol. N.Y.*, **76**, 563.—(1951*b*) *Endocrinology*, **48**, 1.—(1952) *Arch. Path.*, **53**, 22.
*Iidem*, Lindsay, S. and Feller, D. D.—(1950) *Endocrinology*, **46**, 72.
Gorbman, A.—(1946) *Cancer Res.*, **6**, 492.—(1947) *Ibid.*, **7**, 746.—(1949) *Proc. Soc. exp. Biol. N.Y.*, **71**, 237.—(1951) *J. clin. Endocrin.*, **10**, 1177.—(1952) *Proc. Soc. exp. Biol. N.Y.*, **80**, 538.
*Idem* and Edelmann, A.—(1952) *Ibid.*, **81**, 348.
Gorrell, D. S.—(1952) *Canad. med. Ass. J.*, **67**, 425.
Gould, A. P.—(1900) *Clin. J.*, **16**, 33.
Griesbach, W. E.—(1941) *Brit. J. exp. Path.*, **22**, 245.
*Idem*, Kennedy, T. H. and Purves, H. D.—(1945) *Ibid.*, **26**, 18.
*Idem* and Purves, H. D.—(1945) *Ibid.*, **26**, 13.
Griffin, A. C., Rinfret, A. P. and Corsigilia, V. F.—(1953) *Cancer Res.*, **13**, 77.
*Idem*, Rinfret, A. P., Robertson, C. and O'Neal, M.—(1953) *Proc. Amer. Ass. Cancer Res.*, **1**, 21.
György, P. and Goldblatt, H.—(1945) *Science*, **102**, 451.
Haddow, A., Watkinson, J. M. and Paterson, E.—(1944) *Brit. med. J.*, **2**, 393.
Hall, W. H.—(1948) *Brit. J. Cancer*, **2**, 273.
*Idem* and Bielschowsky, F.—(1949) *Ibid.*, **3**, 534.

HANDLER, P. AND FOLLIS, R. H., Jr.—(1948) *J. Nutr.*, **35**, 669.
HANDLEY, W. S.—(1909) *Brit. med. J.*, **1**, 582.
HAUSCHKA, T. S.—(1952) *Cancer Res.*, **12**, 615.
HAZARD, J. B. AND KAUFMAN, N.—(1952) *Amer. J. clin. Path.*, **22**, 860.
HECKEL, N. J. AND KRETSCHMER, H. L.—(1942) *J. Amer. med. Ass.*, 119, 1087.
HELLWIG, C. A.—(1935) *Amer. J. Cancer*, **23**, 550.
HERCUS, C. E. AND PURVES, H. D.—(1936) *J. Hyg., Camb.*, **36**, 182.
HERMANSON, L., GARGILL, S. L. AND LESSES, M. F.—(1952) *J. clin. Endocrin.*, **12**, 112.
HERTZ, R., CROMER, J. K., YOUNG, J. P. AND WESTFALL, B. B.—(1951) *J. nat. Cancer Inst.*, **11**, 867.
HOFFMAN, J., FARELL, D. M. AND HAHN, G. A.—(1953) *Amer. J. Obstet. Gynec.*, **66**, 354.
HOLINGER, P. H., JOHNSTON, K. C. AND ANISON, G. C.—(1950) *Ann. Otol., etc., St. Louis*, **59**, 547.
HOOKER, C. W.—(1948) *Recent Progr. Hormone Res.*, **3**, 173.
*Idem*, PFEIFFER, C. A. AND STRONG, L. C.—(1947) *Cancer Res.*, **7**, 723.
HUBER, H. AND HÖRMANN, G.—(1952) *Z. Krebsforsch.*, **58**, 285.
HUGGINS, C. AND DAO, TH.L-Y.—(1935) *J. Amer. med. Ass.*, **151**, 1388.
HUGUENIN, R., SARACINO, R. AND GERARD-MARCHAND, R.—(1951) *Bull. Ass. franç. Cancer*, **38**, 41.
IVY, A. C.—(1947) *Science*, **106**, 455.
KAHLE, P. J., OGDEN, H. D. AND GETZOFF, P. L., Jr.—(1942) *J. Urol.*, **48**, 83.
KENNEDY, T. H.—(1942) *Nature*, **150**, 233.
KOLLER, P. C.—(1944) *Brit. med. J.*, **2**, 398.
KORTEWEG, R. AND THOMAS, F.—(1939) *Amer. J. Cancer*, **37**, 36.
LACOUR, F., OBERLING, C. AND GUÉRIN, M.—(1951) *Bull. Ass. franç. Cancer*, **38**, 128.
LAFFARGUE, P., LUSCAN, R. AND LAVERNHE, P.—(1952) *Ibid.*, **39**, 290.
LAHEY, F. H. AND HARE, H. F.—(1951) *J. Amer. med. Ass.*, **145**, 689.
*Iidem* AND SALZMAN, F. A.—(1950) *Amer. J. Roentgenol.*, **63**, 880.
LANGHANS, TH. AND WEGELIN, C.—(1919) 'Der Kropf der weissen Ratte.' Bern (P. Haupt).
LAQUEUR, G. L.—(1949) *Cancer Res.*, **9**, 247.
LEATHEM, J. H. AND BARKEN, H. B.—(1950) *Ibid.*, **10**, 231.
LÉGER, J., MASSON, G. M. C. AND PRADO, J. L.—(1947) *Fed. Proc.*, **6**, 150.
LIPSCHUTZ, A.—(1950) 'Steroid Hormones and Tumors.' Baltimore (Williams and Wilkins).—(1952) *Rev. méd. Chile*, **80**, 645.—(1954) *Tex. Rep. Biol. Med.*, **12** (in print).
*Idem*, PONCE DE LEON, H., WOYWOOD, E. AND GAY, O.—(1946) *Rev. canad. Biol.*, **5**, 181.
LUFT, R. AND OLIVECRONA, H.—(1953) *J. Neurosurg.*, **10**, 301.
MACKENZIE, I. AND ROUS, P.—(1941) *J. exp. Med.*, **73**, 391.
MALOOF, F., DOBYNS, B. M. AND VICKERY, A. L.—(1952) *Endocrinology*, **50**, 612.
MARCHANT, J., ORR, J. W. AND WOODHOUSE, D. L.—(1954) *Nature*, **173**, 307.
MCMANUS, R. G. AND SOMMERS, S. C.—(1952) *New Engl. J. Med.*, **246**, 890.
MONEY, W. L., FITZGERALD, P. J., GODWIN, J. T. AND RAWSON, R. W.—(1953) *Cancer*, **6**, 111.
*Idem* AND RAWSON, R. W.—(1947) *Trans. Amer. Ass. Goiter*, 171.—(1950) *Cancer*, **3**, 321.
MOON, H. D., SIMPSON, M. E. AND EVANS, H. M.—(1952) *Science*, **116**, 331.
MOORE, G. E., BRACKNEY, E. L. AND BOCK, F. G .—(1953) *Proc. Soc. exp. Biol. N.Y.*, **82**, 643.
MORRIS, H. P., DALTON, A. J. AND GREEN, C. D.—(1951) *J. clin. Endocrin.*, **11**, 1281.
*Idem* AND GREEN, C. D.—(1951) *Science*, **114**, 44.
NATHANSON, I. T.—(1951) *Radiology*, **56**, 535.—(1952) *Cancer*, **5**, 754.
NELSON, W. O.—(1944) *Yale J. Biol. Med.*, **17**, 217.

NELSON, A. A., FITZHUGH, O. G., MORRIS, H. J. AND CALVERY, H. O.—(1942) *Cancer Res.*, **2**, 11.
PAPANICOLAOU, G. N.—(1949) *Ann. intern. Med.*, **31**, 661.
PARK, W. W. AND LEES, J. C.—(1950) *Arch. Path.*, **49**, 73, 205.
PASCHKIS, K. E., CANTAROW, A. AND STASNEY, J.—(1948) *Cancer Res.*, **8**, 257.
PASQUALINI, C. D. DE AND MANCINI, R. E.—(1951) *Rev. Soc. argent. Biol.*, **27**, 252.
PENNER, D. W.—(1953) *Cancer*, **6**, 776.
PETERS, H.—(1950) *Ibid.*, **3**, 481.
*Idem* AND FRANK, I. N.—(1952) *Surg. Gynec. Obstet.*, **94**, 69.
PLATT, L. I.—(1952) *Amer. J. clin. Path.*, **22**, 662.
PRESTON, F. W., TAYLOR, S. G., III, AND CRUMRINE, J. L.—(1949) *J. clin. Endocrin.*, **9**, 1314.
PURVES, H. D. AND GRIESBACH, W. E.—(1946) *Brit. J. exp. Path.*, **27**, 294.—(1947) *Ibid.*, **28**, 46.—(1951*a*) *Endocrinology*, **49**, 244.—(1951*b*) *Ibid.*, **49**, 652.
*Iidem* and KENNEDY, T. H.—(1951) *Brit. J. Cancer*, **5**, 301.
RALL, J. E., MILLER, W. N., FOSTER, C. G., PEACOCK, W. C. AND RAWSON, R. W.—(1951) *J. clin. Endocrin.*, **11**, 1273.
RAVEN, R. W.—(1950) *Brit. med. J.*, **1**, 1343.
RAWSON, R. W., SKANSE, B. N., MARINELLI, L. D. AND FLUHARTY, R. G.—(1949) *Cancer*, **2**, 279.
REAGAN, J. W.—(1952) *Amer. J. clin. Path.*, **22**, 231.
RICHARDSON, H. L., GRIFFIN, A. C. AND RINFRET, A. P.—(1953) *Cancer*, **6**, 1025.
ROMEIS, B.—(1940) 'Handbuch der mikroskopischen Anatomie des Menschen.' Vol. 6, Part 3. Berlin (Springer).
ROUS, P. AND KIDD, J. G.—(1939) *J. exp. Med.*, **69**, 399.—(1941) *Ibid.*, **73**, 365.
RUGH, R.—(1951*a*) *J. Morph.*, **89**, 323.—(1951*b*) *Ibid.*, **89**, 457.
RUPP, J. J.—(1952) *Endocrinology*, **51**, 306.
SAMPSON, J. A.—(1931) *Amer. J. Path.*, **7**, 423.
SCHENKEN, J. R., BURNS, E. L. AND KAHLE, P. J.—(1942) *J. Urol.*, **48**, 99.
SCHULMAN, J., Jr.—(1950) *J. biol. Chem.*, **186**, 717.
*Idem* AND KEATING, R. P.—(1950) *Ibid.*, **183**, 215.
SEIDLIN, S. M., MARINELLI, L. D. AND OSHRY, E.—(1946) *J. Amer. med. Ass.*, **132**, 838.
SEIFTER, J., EHRICH, W. E. AND HUDYMA, G. M.—(1949) *Arch. Path.*, **48**, 536.
*Iidem* AND MUELLER, G.—(1946) *Science*, **103**, 762.
SELLERS, E. A., HILL, J. M. AND LEE, R. B.—(1953) *Endocrinology*, **52**, 188.
*Idem* AND YOU, R. W.—(1951) *J. Nutr.*, **44**, 513.
SHAY, H., HARRIS, C. AND GRUENSTEIN, M.—(1952) *J. nat. Cancer Inst.*, **13**, 307.
SHIMKIN, M. B., BOLDREY, E. B., KELLY, K. H., BIERMANN, H. R., ORTEGA, P. AND NAFFZIGER, H. C.—(1952) *J. clin. Endocrin.*, **12**, 439.
SILBERBERG, R. AND SILBERBERG, M.—(1953) *Proc. Amer. Ass. Cancer Res.*, **1**, 52.
SIRTORI, C.—(1951) 'Atti del VI° Congresso Nazionale.' Lega Italiana Per La Lotta Contro i Tumori.
*Idem* AND GRATTAVOLA, R.—(1947) *Tumori*, **33**, 319.
SMITH, G. V. S.—(1941) *New Engl. J. Med.*, **225**, 608.
SOMMERS, S. C. AND TELOH, H. H.—(1952) *Arch. Path.*, **53**, 160.
SPEERT, H., QUIMBY, E. H. AND WERNER, S. C.—(1951) *Surg. Gynec. Obstet.*, **93**, 230.
STEWART, F. W.—(1952) *Tex. Rep. Biol. Med.*, **10**, 239.
SUMNER, W. C.—(1953) *Cancer*, **6**, 1040.
TAYLOR, S.—(1953) *J. clin. Endocrin.*, **13**, 1232.
TREVES, N.—(1949) *Cancer*, **2**, 191.—(1953) *Surgery*, **34**, 810.
*Idem*, ABELS, J. C., WOODARD, H. Q. AND FARROW, J. H.—(1944) *Surg. Gynec. Obstet.*, **79**, 589.

Upton, A. C. and Furth, J. (1953) *Proc. Soc. exp. Biol. N.Y.*, **84**, 255.
Walsh, Th. E. and Beamer, P. R.—(1950) *Laryngoscope, St. Louis*, **60**, 1110.
Warren, S., Alvizouri, M. and Colcock, B. P.—(1953) *Cancer*, **6**, 1139.
Wegelin, C.—(1927) *Schweiz. med. Wschr.*, **57**, 848.—(1928) *Cancer Rev.*, **3**, 297.
Woll, E., Hertig, A. T., Smith, G. V. S. and Johnson, L. C.—(1948) *Amer. J. Obstet. Gynec.*, **56**, 617.
Wollman, S. H., Morris, H. P. and Green, C. D.—(1951) *J. nat. Cancer Inst.*, **12**, 27.
*Idem*, Scow, R. O. and Morris, H. P.—(1953) *Ibid.*, **14**, 593.
Wynder, E. L.—(1952) *New Engl. J. Med.*, **246**, 573.
Zalin, H.—(1948) *J. Laryng.*, **62**, 621.
Zeckwer, I. T.—(1953) *Endocrinology*, **53**, 326.